# The consequences of antipsychotic medication use for people living with dementia: a systematic review and meta-analysis

**DOI:** 10.3389/fpsyt.2026.1817609

**Published:** 2026-05-08

**Authors:** Poe Eindra Thant, Alina Zenker, Johan Jarl, Ulf-Göran Gerdtham, Cecilia Lenander, Sofie Persson, Sanjib Saha

**Affiliations:** 1 Health Economics Unit, Department of Clinical Science (Malmö), Lund University, Lund, Sweden; 2 Department of Economics, Lund University, Lund, Sweden; 3 Centre for Economic Demography, Lund University, Lund, Sweden; 4 Center for Primary Health Care Research, Department of Clinical Sciences Malmö, Lund University, Malmö, Sweden; 5 The Swedish Institute for Health Economics, Lund, Sweden

**Keywords:** antipsychotic drug use, consequences, dementia, meta-analysis, systematic review

## Abstract

Antipsychotic drugs (APD) are commonly prescribed to people living with dementia (PwD) to manage behavioral and psychological symptoms of dementia (BPSD), despite well-documented safety concerns. International guidelines recommend non-pharmacological interventions as first-line treatment, yet APDs remain widely used. This systematic review and meta-analysis synthesize current evidence on the consequences of APD use in PwD. Following PRISMA guidelines, we conducted a comprehensive search of PubMed, EMBASE, Cochrane Library, and Web of Science for studies published between January 2010 and August 2024. The protocol was registered in PROSPERO on 25 March 2022 (CRD42022312570). Eligible studies included observational and interventional designs reporting APD use in PwD. Risk of bias was assessed using the Cochrane tool for randomized trials and the Newcastle-Ottawa Scale for observational studies. Hazard ratios (HRs) were pooled using a random-effects meta-analysis. Subgroup analyses were performed by APD type (typical vs. atypical), study design, risk of bias, and patient setting. The certainty of the evidence was evaluated using GRADE. Forty-five studies comprising two million participants were included. Twenty-five studies reported mortality outcomes. Meta-analysis showed APD use was associated with a significantly increased mortality risk (pooled HR = 1.32; 95% CI 1.12, 1.56), with considerable heterogeneity (I2 = 98.86%). Subgroup analysis indicated similar risk elevation for both typical and atypical APDs and higher hazards among community-dwelling individuals. Evidence for other adverse outcomes, such as stroke, pneumonia, hip fractures, and hospitalization, was limited and heterogeneous, though several individual studies indicated elevated risks. Meta-analysis of cerebrovascular events showed an attenuated but non-significant association (pooled HR = 1.77; 95% CI 0.92-3.42). Conclusion: APD use in PwD is consistently associated with increased mortality and may even elevate risks for serious non-fatal events. These findings emphasize the necessity of cautious prescribing, regular medication review and close monitoring for PWD. Future research can explore drug-specific effects, dose-response relationships, and long-term outcomes, particularly in low-resource settings.

**Systematic review registration:**

https://www.crd.york.ac.uk/prospero/, identifier CRD42022312570.

## 1 Introduction

Dementia affects over 55 million people worldwide, with the majority residing in low- and middle-income countries (1). This number is projected to reach 78 million by 2030 and 139 million by 2050 (2). Dementia imposes a significant public health and economic burden, with global costs estimated at US$ 1.3 trillion and expected to surpass US$ 2.8 trillion by 2030, driven by both direct medical expenses and substantial indirect costs, in particular informal care (2).

Behavioral and psychological symptoms of dementia (BPSD), also termed neuropsychiatric symptoms (NPS) (3), affect most people living with dementia (PwD), with recent meta-analyses indicating a pooled prevalence of approximately 60% in acute hospital settings and up to 85% at some point during the disease trajectory (4). These symptoms—including aggression, agitation, psychosis, depression, apathy, and sleep disturbances—pose significant challenges for care quality and patient well-being (5). The US Food and Drug Administration issued a boxed warning in 2005 about the increased mortality risk associated with atypical antipsychotics use in older adults with dementia-related psychosis and extended the warning to all antipsychotics in 2008 due to the findings about typical antipsychotics having similar or higher mortality risk in older adults with dementia (6). The European Medicines Agency continues to restrict the use of antipsychotics, with risperidone as the only approved antipsychotics drug APD for short-term treatment of persistent aggression in moderate to severe Alzheimer’s dementia, and only when non-pharmacological interventions have failed (7). Although antipsychotic drugs may confer modest benefits for select behavioral and psychological symptoms of dementia—specifically aggression and psychosis—no available regimen is without significant safety concerns (8). This necessitates a careful, individualized risk–benefit evaluation, routine reassessment of ongoing therapy, and vigilant monitoring for potential adverse effects throughout treatment.

A large amount of systematic literature review and meta analyses demonstrate that APDs use in dementia are associated with significantly increased risks of adverse events, including cerebrovascular accidents/stroke, myocardial infarction, pneumonia, acute kidney injury, falls, fractures, hospitalizations, and death (9–17). These risks are most pronounced during the initial months of treatment but persist throughout the course of therapy (18). However, the previous reviews have had notable limitations: many restricted their population to only one or two types of dementia—commonly Alzheimer’s and vascular dementia (19, 20)—while failing to include other subtypes such as Lewy body or frontotemporal dementia. In addition, most focused exclusively on atypical antipsychotics (8, 9, 21–25), typically risperidone (24), olanzapine, brexpiprazole, and aripiprazole (8), rarely considering the risks and benefits of both typical and atypical antipsychotics. The measured outcomes were often limited to a narrow set, such as mortality (22, 23, 26), cerebrovascular events (21, 26–29) or reduction in aggression and psychosis (30), with inconsistent attention to broader impacts including quality of life, cognitive function, institutionalization, and adverse events (5). Only a single comprehensive review in 2005 addressed all dementia subtypes, all antipsychotic classes, and multiple relevant outcomes but without meta-analysis (31), meaning much of the evidence base is now outdated given changes in prescribing practice, the emergence of new drugs, regulatory updates, and publication of numerous new studies. Methodologically, many previous reviews have overly relied on randomized controlled trials (5, 8, 9, 22, 26, 32, 33), excluded observational data vital for understanding real-world effectiveness and safety, and lacked protocol registration or transparency, increasing potential for bias. Therefore, there is a clear need for a rigorous, up-to-date systematic literature review that comprehensively includes all dementia populations, diverse antipsychotics, and clinically meaningful outcomes, using robust contemporary methods to inform clinical practice and regulatory decisions.

## 2 Methods

This systematic review follows the PRISMA guidelines (34) and was registered in PROSPERO (ID: CRD42022312570) on 25 March 2022.

### 2.1 Literature search

A comprehensive search was conducted in PubMed/MEDLINE, CINAHL, Cochrane, EMBASE, and Web of Science for studies published between January 1, 2010, and August 14, 2024. A professional librarian assisted in developing the search strategy which is available in the **Supplementary Material**.

### 2.2 Study selection

References were imported into EndNote and then uploaded to Covidence. After removing duplicates, two reviewers (PT, AZ) independently screened titles and abstracts for eligibility using population, exposure, comparison, outcome, study design (PECOS) criteria (**Supplementary Table 1**); disagreements were resolved by discussion. Full texts were assessed similarly.

Studies focusing exclusively on Parkinson’s disease dementia were excluded due to the distinct neuropsychiatric symptom profile and frequent use of antipsychotics such as quetiapine and clozapine specifically for Parkinson’s psychosis (35). Studies were also excluded if antipsychotic drugs were used only as a comparator while other psychotropic medications were the intervention. However, studies examining other psychotropic agents (e.g. antidepressants, benzodiazepines) were eligible if antipsychotic effects were reported separately. Reference lists of included studies were screened for additional relevant studies.

### 2.3 Data extraction

Two reviewers (PT, AZ) independently extracted data; disagreements were resolved by a third reviewer (SS). Hazard ratio (HR) was the primary effect estimate for time-to-event outcomes (36). When HR was unavailable, other measures such as prevalence rate (%), incidence rate ratio (IRR), odds ratio (OR), relative risk (RR), and prior event rate ratio (PERR) were extracted. Adjusted estimates were prioritized.

### 2.4 Risk of bias assessment

Randomized trials were assessed using the Cochrane risk of bias tool (37), covering six domains: random sequence generation and allocation sequence concealment for selection bias, blinding of participants and personnel for performance bias, blinding of outcome assessment for detection bias, incomplete outcome data for attrition bias, selective outcome reporting for reporting bias, and ‘other’ potential sources of bias. Summary assessment of risk of bias for each study was judged as having “low risk of bias” if the trial is judged to be at low risk of bias for all domains, “unclear” if low or unclear risk of bias for all key domains, and “high” if risk of bias was high for one or more of key domains for the overall risk of bias (37).

For observational studies, we used the adapted Newcastle-Ottawa Scale (NOS) (38), which evaluates selection, comparability, and outcomes, plus exposure for case-control studies. Studies were rated as “good quality” (7–9 points), “fair quality” (4–6 points), or “poor quality” (0–3 points). Poor quality studies were considered high risk of bias; fair and good-quality studies were considered low risk. Two reviewers (PT, AZ) conducted assessments independently; discrepancies were resolved by consensus.

### 2.5 Data synthesis

Outcomes were categorized systematically. Meta-analyses utilized a random-effects DerSimonian-Laird method. Adjusted HRs from Cox models comparing APD use (any, typical, atypical) with non-use were pooled using inverse-variance weighting (39). When both typical and atypical HRs were reported, atypical HRs were prioritized for clinical relevance. HRs were log-transformed for analysis and back-transformed for interpretation (40). Heterogeneity was evaluated using I²; values greater than 50% prompted subgroup analyses by risk of bias/study quality, design, and APD type. Forest plots illustrated pooled and individual effects. Publication bias was evaluated via funnel plots and Egger’s test (41).

Analyses were performed using RevMan 5.4 (42) and STATA 17. Certainty of evidence was rated using GRADE (43) as high, moderate, or low based on risk of bias, consistency, directness, precision, and publication bias.

## 3 Results

### 3.1 Study selection and characteristics

**Figure 1:**
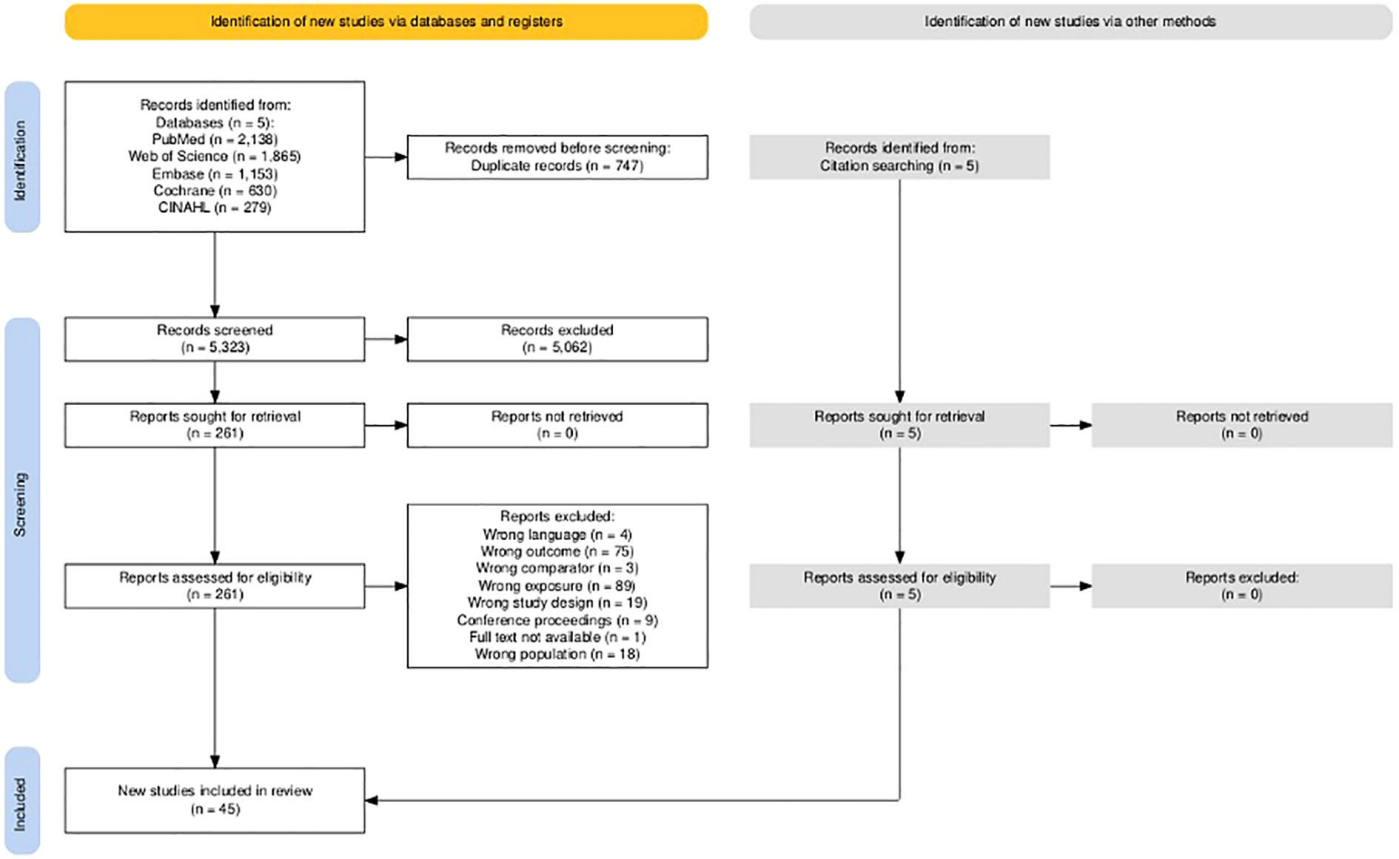
PRISMA flow diagram of study selection.

The PRISMA flow diagram (**Figure 1**) summarizes the identification and selection process. Forty-five studies were included in this review: 31 follow-up cohort studies (10, 12, 13, 18, 46–50,15,52-72), two population based studies (44, 45), two longitudinal observation studies (46, 47), a register study (48), two case-control studies (49, 50), one non-randomized intervention study, one randomized trial and one clinical trial (51–53), two cross-sectional studies (54, 55), and one retrospective analysis (56). Most studies (60%) did not specify dementia subtype (n = 27) (10, 13, 15, 18, 44, 45, 47–50, 54, 55, 57–71). Thirty-three percent focused on Alzheimer’s disease (AD) (n = 15) (46, 51–53, 56, 72–81), and the remaining studies comprised one study on vascular dementia (82), and one on mixed dementia types (83). Participants were from both community-dwelling and institutional care settings. Most of the studies were from European countries [Europe (nine countries) (n = 1) (53), the United Kingdom (n = 7) (15, 18, 50, 57, 64, 70, 82), Finland (n = 6) (12, 72–75, 80), Denmark (n = 5) (48, 68, 71, 78, 81), Norway (n=2) (44, 54), Sweden (n = 2) (10, 13), France (n = 1) (76), Germany (n = 1) (61), Italy (n = 1) (60), the Netherlands (n = 1) (47)], followed by the United States (n = 12) (46, 49, 52, 55, 56, 58, 62, 63, 65–67, 69), and some Asian and the Pacific countries [China (n = 1) (51), Hong Kong (n = 1) (83), Japan (n = 1) (79), Taiwan (n = 2) (45, 77), and Australia (n = 1) (59)]. Sample sizes ranged from 156 to 20,920,523, with follow-up durations between 24 weeks and 22 years. All studies reported criteria for dementia diagnosis.

### 3.2 Results of individual studies

The included studies were grouped by outcome (**Supplementary Table 2**), which included mortality (n = 25), hospitalization (n = 3), long-term care (LTC)/institutionalization and nursing home admission (n = 3), hip fractures (n = 4), cerebrovascular accidents/stroke (n = 7), falls and orthostatic hypotension (n = 2), and head injury and fractures (n = 2), pneumonia (n = 2), myocardial infarction and heart failure (n = 1), venous thromboembolism (n = 2), acute kidney injury (n = 1), cognitive decline (n = 1), quality of life (QoL) (n = 2), and other adverse events (n = 3).

#### 3.2.1 Mortality

##### 3.2.1.1 Any antipsychotic use vs. non-use

Twenty-five studies reported mortality outcomes. Nearly all studies were credible with the overall NOS scores “good quality”, except for the study by Virginie et al. which was rated as “fair quality” based on the outcome domain (76). Still, based on the comparability domain (whether the studies controlled for confounders including comorbidity) which was considered for credibility, the Virginie et al. study gained two stars for comparability. Among 9 studies comparing any APD use with non-use, five studies reported an adjusted hazard ratio (aHR) above 1 (1.14 to 1.20) (10, 12, 15, 59, 71), and four studies showed statistically non-significant aHR ranging from 0.91 to 1.12 (13, 57, 58, 76). In contrast, only one study, a cohort study in Taiwan, reported reduced risk of mortality associated with the use of antipsychotics in AD patients (aHR = 0.66; 95% CI 0.58, 0.75) (77). Arai et al. reported an increased mortality rate of 9.4% and increased odds of mortality associated with antipsychotic use (aOR = 3.92 (95%CI 1.59, 9.66) (79).

##### 3.2.1.2 Typical APD use vs. non-use

Eight studies analyzed typical APD use compared with non-use in terms of mortality (10, 13, 15, 48, 59–61, 77). All studies were deemed credible based on NOS comparability assessment and rated as overall “good quality”. Five reported an increased risk (aHR 1.16 to 3.7) (10, 48, 59–61), one study reported a reduced risk (aHR 0.69) (77), and two studies showed no statistically significant difference (13, 15).

##### 3.2.1.3 Atypical APD use vs. non-use

Nine studies compared atypical APDs with non-use (10, 13, 15, 48, 59–61, 77, 82). All studies except Sultana et al. (rated “fair quality”) were of good quality in NOS scale and are credible in terms of comparability. Sultana et al. study did not control for comorbidity in their analysis but accounted for other factors such as age, gender, ethnicity, and mini-mental state examination (MMSE) scores. Six studies reported an increased risk (aHR 1.13 to 2.5) (10, 15, 48, 59–61), while one study reported a reduced risk (aHR 0.56) (77), and two studies reported non-significant HRs (13, 82). The study by Yin et al. (NOS 4 stars, “fair quality”) was judged less credible as the study did not account for any confounders in their analysis (51). Yin et al. reported no significant differences associated with atypical APDs when compared with non-benzodiazepine hypnotics, melatonin, or no-drug treatment for 5-year mortality rates and individual death causes and a lower tendency in the 5-year mortality (8.7 vs. 12.12 vs. 11.11 vs. 13.7%) among patients treated with atypical antipsychotics, non-benzodiazepine hypnotic, melatonin, or without sleep medicine, respectively in a non-randomized interventional study (51).

##### 3.2.1.4 Typical vs. atypical APDs

Four studies compared typical APDs with atypical APDs (13, 15, 59, 60). Based on our NOS scale assessment, these four studies are credible in methodological quality (NOS scale “good quality”). Only one study found a statistically significant increase in mortality risk of typical APD (aHR = 1.5; 95% CI 1.1, 2.1), while the remaining study found no statistically significant difference (60).

##### 3.2.1.5 Individual drugs

All studies that reported on individual drugs effect were credible based on comparability assessment except for Phiri et al. (overall “good quality” and Sultana et al. overall “fair quality” studies which did not control for comorbidity but controlled age, gender, ethnicity and MMSE scores (64, 82). One study reported that haloperidol had the highest 180-day mortality risk among other APDs compared with risperidone (aHR = 1.54; 95% CI 1.38, 1.73) (62). A German cohort study found a similar result for haloperidol (aHR = 1.56; 95% CI 1.38, 1.75) compared with the non-use of haloperidol (61). Another study also reported an increased mortality risk with haloperidol compared to risperidone within 30 days (aHR = 1.7; 95% CI 1.0, 3.0) (44). Maust et al. reported an increased absolute mortality risk of haloperidol of 3.8% with number needed to harm of 26 (63). Risperidone was found to be associated with an increased risk of mortality in the US (63). Nerius et al. also reported an increased risk of death for risperidone (aHR = 1.28; 95% CI 1.17, 1.40) compared to the use of other APDs (61). Phiri et al. (64) studied individual drugs and found increased mortality risk with both olanzapine (aHR = 1.32; 95% CI 1.08, 1.60) and risperidone (aHR = 1.35; 95% CI 1.18, 1.54) but an insignificant effect with quetiapine (aHR = 1.09; 95% CI 0.90, 1.34) (64). Two additional studies did not find significant risk with quetiapine use (61, 82). However, compared with risperidone, quetiapine had a lower risk of mortality (aHR = 0.73; 95% CI 0.67, 0.80) in one study (62) and an increased risk was found between 730–2400 days of exposure to quetiapine (aHR = 1.4; 95% CI 1.0, 1.9) compared to exposure to risperidone (44).

##### 3.2.1.6 Studies with dose-dependent outcomes and comparison of exposure periods

Four studies examined the impact of treatment duration and are credible based on our comparability assessment and judged as good quality for overall NOS assessment. Short-term use (0–30 days) was associated with higher mortality risk compared to long-term use (>30 days) (44, 65, 78), although Simoni-Wastila et al. found no statistically significant association between mortality and duration of use (66). Nielsen et al. examined trends for mortality risk and showed that antipsychotic use was associated with increased mortality risk in all investigated time periods, the HR being 2.24 (95% CI 2.07, 2.43) in 2000–2002 and declining to 1.24 (95% CI 1.09, 1.41) in 2009–2011 (81).

#### 3.2.2 Hospitalization

All three studies that reported the outcome of hospitalization were of good credibility and rated overall “good quality”. Zakarias et al. found higher hospitalization risk when APDs were combined with benzodiazepines compared to APD monotherapy (68). However, Mueller et al. found no difference for any APD, typical or atypical APDs vs. non-use (15). The number of days of hospitalization was higher (52.5 days) with APD initiators/new users than non-initiators (34.7 days) among Finnish community dwellers (72).

#### 3.2.3 Long-term care and nursing home admission

A German health claims data study with good quality NOS scores found that typical APD use was associated with a two-fold increase in the risk of long-term care and a 50 percent higher likelihood of moving to nursing homes from private living, compared to non-users. Atypical APD use compared to non-use showed no statistically significant association with the same two outcomes. Use of individual APDs versus not using the designated APD significantly increased the hazard of receiving long-term care support by 1.64-fold (quetiapine), 2.08-fold (risperidone), 2.12-fold (haloperidol) and 2.34-fold (melperone), respectively. The hazard of nursing home admission for the same individual APDs was 40% to 74% higher than if not being exposed to them (61). However, an observational study from the US found no statistically significant association between the use of typical and atypical APDs and the risk of nursing home admission (46). Notably, Lopez et al. study was rated fair quality based on domains being less representative as the study sample size was only 957 Alzheimer’s patients who were from a research program and the ascertainment of exposure being absent at the start of the study and the outcome assessment were not by record linkage or independent blind assessment. A study from China by Yin et al., found a lower rate of institutionalization among AD patients with sleep disturbances who were taking atypical APDs compared with AD patients with sleep disturbances without use (51). It is noteworthy that this study was less credible than the others based on their lack of robust control for confounders in the analysis and NOS scores were granted “fair quality” with four stars.

#### 3.2.4 Hip fractures

An increase in the risk of hip fractures was reported in four studies from Denmark, Finland, the US, and Wales, three “good quality studies” and one “fair quality” study by Jalbert et al. (49, 57, 68, 74). The study designs of these studies varied between a nationwide register-based cohort, a nested case-control, and an e-cohort study. The results were reported in different ratios, such as hazard ratio, odds ratio, and the prior event rate ratio. Two studies reported an aHR of 1.26 (95% CI 1.05, 1.52) (49) and 1.54 (95% CI 1.39, 1.7) (74). The latter further showed statistically significantly increased risk when examining different time periods during the follow up. Dennis et al. estimated the prior event rate ratio, which also demonstrated an increased risk of hip fracture within 12 months of APD use versus non-use (PERR 1.62 (95% CI 1.59, 1.65) (57). One study did not find a statistically significant association between APD use and risk of hip fracture (68).

#### 3.2.5 Stroke and cerebrovascular accidents and events

All studies in this outcome group were of good quality according to NOS scale, and only study by DeMercy and Brenner that was rated “fair quality” overall was considered less credible because of the lack of control for comorbidity or dementia severity (69). Mok et al. reported an increased hazard of stroke within 180 days of APD use among adults with dementia diagnoses (aHR = 1.54; 95% CI 1.46, 1.63). The risk was even higher within 7 days of use (aHR = 3.75; 95% CI 3.00, 4.69) (18). A population-based cohort study in Taiwan also reported an increased risk of stroke among PwD who use APDs with an aHR of 1.17 (95% CI 1.01, 1.40) (45). One study from the UK found an increased prior event rate ratio of stroke of 1.41 (95% CI 1.4, 1.46) (57). However, other studies showed no significant association between use of antipsychotics and stroke. A Finnish study among community dwellers with a clinically verified AD diagnosis reported an overall non-significant association (aHR = 1.09; 95% CI 0.98, 1.22) but the hazard of stroke was increased in the first 60 days of use (aHR = 1.73; 95% CI 1.32, 2.28) (73). Quetiapine did not increase the risk of stroke significantly compared to risperidone (aHR = 1.12; 95% CI 0.91, 1.37) (73). The hazard of major adverse cardiac/cerebrovascular events (MACCE) was found to be 1.79 (95% CI 1.53, 2.10) in American individuals aged 50 and above for APD users versus non-users, although apart from Haloperidol only atypical APDs were included (69). Two studies reported statistically non-significant hazard and odds ratios for CVA in cohorts of PwD who use APDs versus who do not (50, 83). The use of atypical APDs was found to decrease the odds of a CVA (aOR = 0.62; 95% CI 0.53, 0.72), typical APDs were associated with higher odds of a CVA when compared with atypical APDs use although the study found no statistically significant association when comparing APD use with non-use in overall ORs (50).

#### 3.2.6 Falls, orthostatic hypotension

Incident falls were increased (incidence rate ratio IRR = 1.72; 95% CI 1.04, 2.85) among mild to moderate AD patients in a clinical trial study in Europe which was rated as “good quality” NOS scores (53). The study also reported increased risk of sit-to-stand OH among the participants (OR = 1.23; 95% CI 1.05, 1.43) (53).

#### 3.2.7 Head injuries and fractures

A Finnish study found an increased risk of head injuries (aHR = 1.29 (95% CI 1.14, 1.47) and traumatic brain injuries (aHR = 1.22 (95% CI 1.03, 1.45) among 43,590 community dwellers with clinically diagnosed AD who used APDs (75). An increased risk of fracture within 180 days was reported as being associated with current use of APD in a population-matched cohort study in England (aHR = 1.43; 95% CI 1.35, 1.52), the risk was highest within a week of use (aHR = 2.22; 95% CI 1.66, 2.98) (18). Both studies were credible and of good quality scores in NOS scale.

#### 3.2.8 Pneumonia

A matched cohort study in Finland reported an increased risk of pneumonia associated with APD use among community dwellers with AD (aHR = 2.01 (95% CI 1.90, 2.13). The risk was even higher among individuals without AD (80). Another study in England similarly reported an increased risk of pneumonia associated within 180 days of current use of APDs (aHR = 2.19; 95% CI 2.10, 2.28) (18). The risk was highest within one week of use (aHR = 9.99; 95% CI 8.78, 11.40) (18). Both of the studies were rigorous in methodology with “good quality” NOS scores.

#### 3.2.9 Myocardial infarction and heart failure

Two studies reported on cardiovascular adverse events and was assessed “good quality” (18, 57). Mok et al. reported an increased risk associated with current use of APD in both myocardial infarction (aHR = 1.28; 95%CI 1.15, 1.42), with the highest risk within one week of use (aHR = 2.33; 95% CI 1.41, 3.83), and heart failure (aHR = 1.27; 95% CI 1.18, 1.37), with the risk even higher within one week of use (aHR = 2.85; 95% CI 2.15, 3.78) (18). Furthermore, a register-based study in Wales showed an increased likelihood of an acute cardiac event (ACE) within 12 months of APD use for persons with AD [PERR 1.65 (95% CI 1.59, 1.86)]. For all dementia types, no statistically significant PERR could be demonstrated, though atypical APD use versus typical APD use was associated with a higher likelihood of acute cardiac event (ACE) [PERR = 3.3 (95% CI 31,0, 5,1] (57).

#### 3.2.10 Venous thromboembolism

Two studies with good quality NOS scores reported on the risk of venous thromboembolism. Increased risk of venous thromboembolism was associated with current use of APD among adults with dementia (aHR = 1.62 (95% CI 1.46, 1.80), and the risk was highest within one week of APD use (aHR = 2.05; 95% CI 1.19, 3.56) (18). The likelihood of a VTE within 12 months of APD use versus non-use was estimated via the PERR at 1.95 (95% CI 1.83, 2.00) for all dementia types and at 1.80 (95% CI 1.67, 1.89) for persons with AD (57).

#### 3.2.11 Acute kidney injury

Current use of APD was associated with an increased risk of acute kidney injury (aHR = 1.72; 95% CI 1.61, 1.84), with the highest risk within one week (aHR = 3.79; 95% CI 2.96, 4.87); the risk remained high for up to two years (18).

#### 3.2.12 Cognitive decline

A randomized trial with a high risk of bias (especially in domain of blinding, detection bias) could not demonstrate a statistically significant change in cognitive function in persons with AD who used APDs compared to non-users (52).

#### 3.2.13 Quality of life

Two “fair quality” studies found no significant association between APD use and quality of life (47, 54). The study by van de Ven-Vakhteeva et al. studied for 2 years longitudinally on the PwD in nursing homes and controlled for age, sex, neuropsychiatric inventory in nursing homes (NPI-NH), severe impairment battery-short version (SIB-s) for cognitive severity, and activities of daily living, minimum data set, short form (ADL-MDS-SF) scores which is a robust measure for physical frailty and functional impairment. Ito et al. conducted a secondary analysis of 431 nursing home patients form COSMOS trial (the multicomponent intervention consisting of communication, systematic pain assessment and treatment, medication review, organization of activities, and safety), and only controlled for NPI-NH total score, age, and gender and was considered less credible.

#### 3.2.14 Adverse events

In a descriptive statistical analysis study, Bangash et al. reported that the rate of adverse events such as muscle stiffness, falls, cognitive rigidity, tremors, and increased agitation was more often seen in risperidone users compared to haloperidol, quetiapine, aripiprazole, olanzapine, and amisulpride users (70). Sepassi and Watanabe reported a higher likelihood of individuals with AD experiencing any APD drug-associated adverse events, compared to individuals without AD, in their retrospective analysis (56). Beeber et al. reported a descriptive analysis of potential side effects of antipsychotic use among assisted living residents with dementia, and 93 (19) percent had more than one (5) side effects (55). The most common side effects reported were neurological/psychological, followed by adverse events such as seizures, transient ischemic attacks, and hip fractures (55).

### 3.3 Effect modifying factors

Age and sex have been reported as important factors modifying the effect of APD on mortality risk among PwD. Langballe et al. found that the use of APD was linked to a higher mortality risk among people with dementia (PwD) as age increased (44). Nielsen et al., on the other hand, reported a lower mortality rate for older age at the time of diagnosis in persons with AD with APD use (aHR = 0.76; 95% CI (0.76, 0.76) (78). Langballe et al. and Arai et al. found that APD use was associated with an increased mortality risk in men compared with woman with dementia (44, 79).

In terms of place of residence, Nerius et al. found an excess mortality rate associated with haloperidol and melperone use among PwD residing in private households compared to nursing home residents (61).

### 3.4 Result of meta-analyses

#### 3.4.1 Risk of mortality

**Figure 2:**
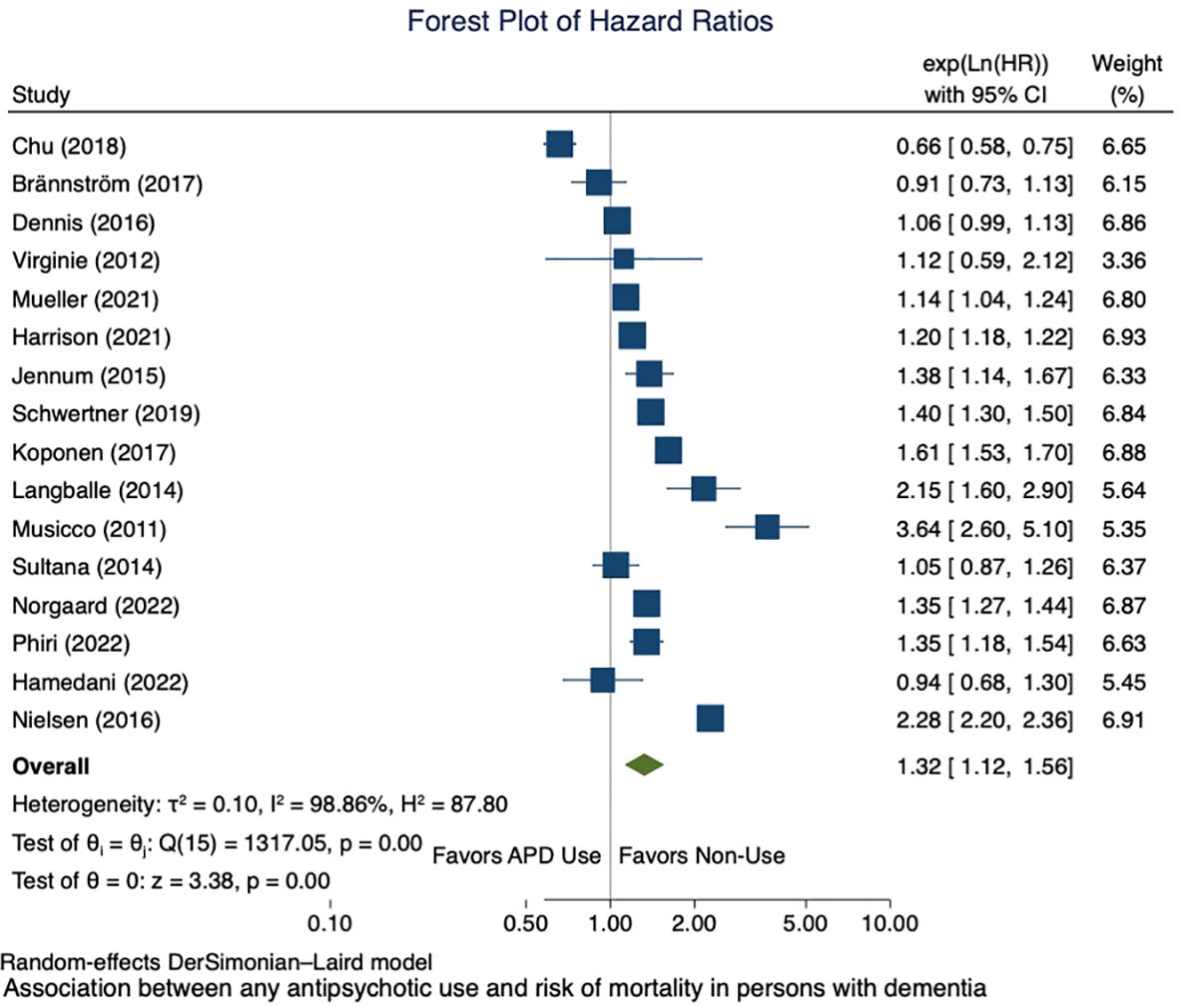
Forest plot of the association between any antipsychotic use and risk of mortality in persons with dementia.

**Figure 3:**
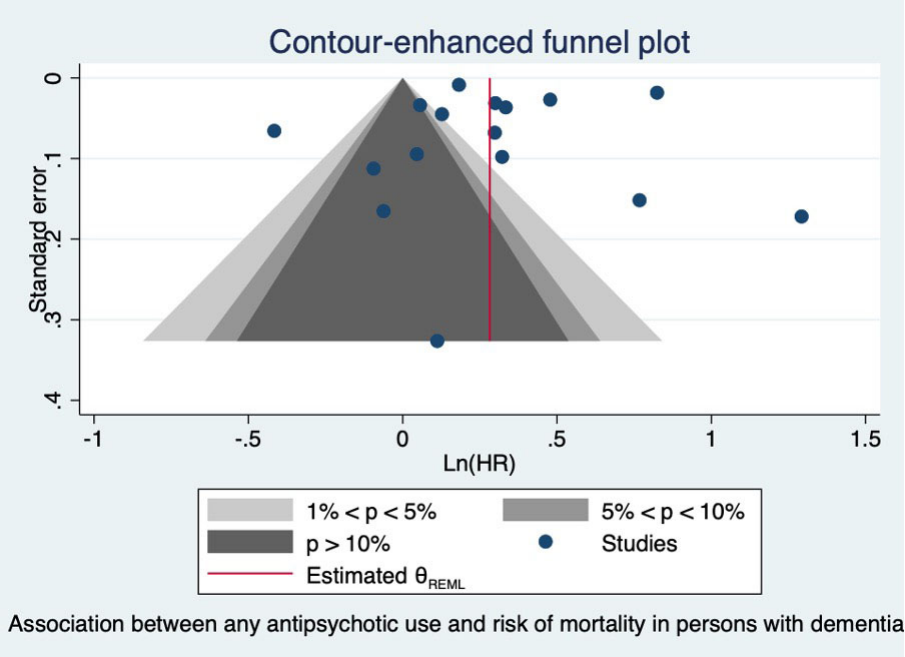
Funnel plot of the association between any antipsychotic use and risk of mortality in persons with dementia.

Meta-analysis for the association between the use of antipsychotics and mortality risk is presented in **Figure 2**. Thirteen studies reported the association between any use of antipsychotics and mortality risk. We included two studies that reported on atypical APD use (48, 64) and one that reported on typical APD use only in the analysis (60). Any APD use was found to be associated with a significantly increased risk in mortality compared to non-users in PwD (pooled HR = 1.32; 95% CI 1.12, 1.56). High statistical heterogeneity was noted (I² = 98.86%), indicating large variations between studies. The funnel plot for any antipsychotic use associated with mortality risk shows asymmetry (**Figure 3**); however, Egger’s test did not indicate the presence of any small study effect (p = 0.7473).

#### 3.4.2 Subgroup analysis on the risk of mortality

Subgroup analysis based on type of antipsychotics showed typical antipsychotic use (number of studies, n = 6) was associated with an increased risk of mortality in PwD compared to non-use of antipsychotics (pooled HR = 1.25; 95% CI 1.01, 1.55) with considerable heterogeneity (I² = 95.29%) (**Supplementary Figure 1**). Atypical antipsychotic use was associated with an increased risk of mortality among individuals with dementia (pooled HR = 1.23; 95% CI 1.05, 1.43) with an I² of 94.15% (**Supplementary Figure 2**). Subgroup analyses based on accommodation type showed that community dwelling PwD had higher HR (1.53; 95% CI 1.18, 1.98) compared to PwD living in clinical, nursing home and nursing plus community dwelling PwD (**Supplementary Figure 3**). Analyses restricted to studies with high-quality NOS scores showed an overall HR of 1.35 (95% CI 1.14, 1.61) (**Supplementary Figure 4**). Subgroup analysis on study design showed that retrospective cohort studies (n = 13) on any antipsychotic use in PwD had even higher HR (1.43; 95% CI 1.21, 1.71) (**Supplementary Figure 5**).

For typical antipsychotic use, the funnel plot shows asymmetry (**Supplementary Figure 6**) and Egger’s test indicates the presence of small study effect (p = 0.0086). For studies on atypical APD use and risk of mortality, the funnel plot is symmetrical, and Egger’s test did not indicate the presence of small study effect (p = 0.5596) (**Supplementary Figure 7**).

#### 3.4.3 Risk of cerebrovascular adverse events

**Figure 4:**
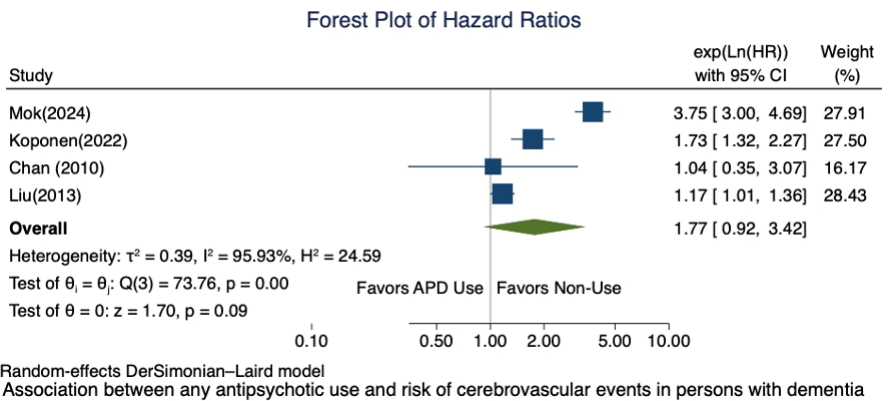
Forest plot of the association between any antipsychotic use and risk of cerebrovascular events in persons with dementia.

**Figure 5:**
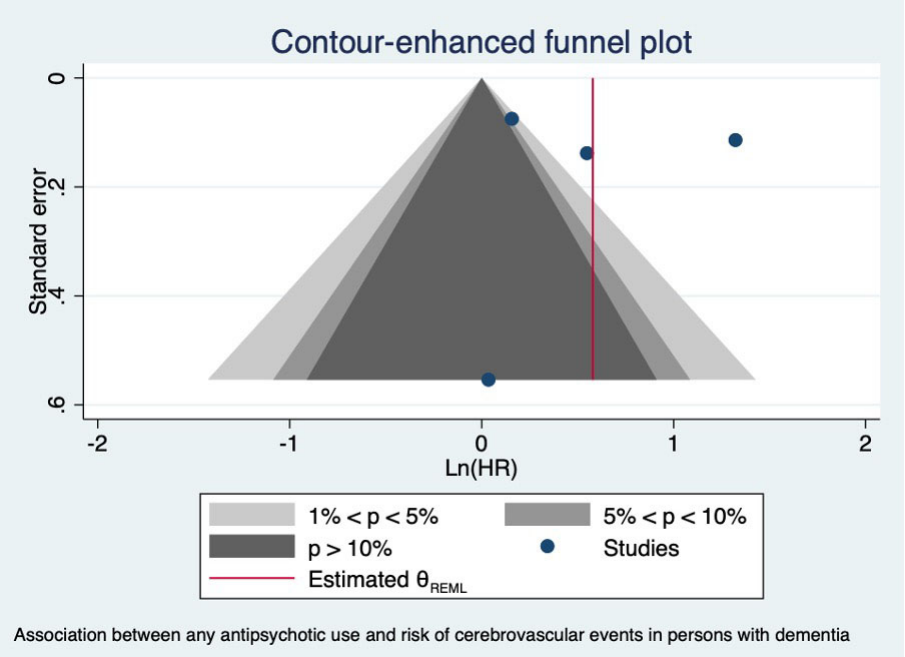
Funnel plot of the association between any antipsychotic use and risk of cerebrovascular events in persons with dementia.

We conducted a meta-analysis of the four studies that reported cerebrovascular adverse events, including stroke, with an adjusted HR (18, 45, 73, 83). We excluded the studies that reported other effect estimates such as PERR or odds ratio. There was an attenuated but not significant risk associated with antipsychotic use and risk of cerebrovascular adverse events (pooled HR = 1.77; 95% CI 0.92, 3.42) with considerable heterogeneity (I² = 95.93%), which indicates study differences between the existing studies (**Figure 4**). The funnel plot shows asymmetry (**Figure 5**), but Egger’s test did not indicate the presence of small-study effects (p = 0.5618).

### 3.5 Risk of bias in studies

Overall, the quality of the observational evidence was high. One nested case-control (77) and seven retrospective cohort studies (10, 45, 48, 58, 60, 65, 78) achieved a rating of “good quality” (9 stars on the NOS scale) demonstrating strengths particularly in selection domain (**Supplementary Table 3**). In contrast, nine studies achieved only a score of 5 or 6, resulting in fair-quality studies (49, 54, 69, 70) (46, 47, 51, 76, 82). All the other included studies were rated of good quality, with a score of 7 or 8 on the NOS scale. The single randomized trial was of high risk of bias assessed by the Cochrane risk of bias tool (**Supplementary Table 4**) (52). The study demonstrated low risk of bias in blinding of participants and personnel, incomplete outcome data, and selective reporting, though there was a high risk of bias in detection bias.

### 3.6 GRADE assessment

The summary of findings table for any APD use on mortality and cerebrovascular adverse events are presented in **Tables 1**, **2**, whereas for typical and atypical APD is presented in **Tables 3**, **4**. The overall certainty of the findings for risk of mortality in this review, assessed by the GRADE approach, is high (**Supplementary Table 5**). The overall certainty of the findings for risk of cerebrovascular adverse events is low due to the high inconsistency (**Supplementary Table 5**). There was a low risk of bias among the studies according to the risk of bias assessment, and the funnel plot and Egger’s test indicate a low risk of publication bias. We assessed the indirectness of the results as low and the precision as high. Grade evidence profiles are presented as tables in the appendix (**Supplementary Tables 5**-**7**).

**Table 1:** Summary of findings for any antipsychotic use on risk of mortality.

Summary of findings:				
Any antipsychotics compared to non-use for persons with dementia				
Patient or population: persons with dementia Setting: Community dwelling, nursing home, mixed Intervention: any antipsychotics Comparison: non-use				
Outcomes	Relative effect (95% CI)	№ of participants (studies)	Certainty of the evidence (GRADE)	Comments
Risk of mortality (Mortality risk) assessed with: hazard ratio follow-up: range 2 years to 12 years	HR 1.32 (1.12 to 1.56) [Risk of mortality]	359270 (16 non-randomized studies)^a^	⊕⊕⊕⊕ High^a^	Any antipsychotics increases risk of mortality.
*The risk in the intervention group (and its 95% confidence interval) is based on the assumed risk in the comparison group and the relative effect of the intervention (and its 95% CI). CI, confidence interval; HR, hazard ratio				
GRADE Working Group grades of evidence High certainty: we are very confident that the true effect lies close to that of the estimate of the effect. Moderate certainty: we are moderately confident in the effect estimate: the true effect is likely to be close to the estimate of the effect, but there is a possibility that it is substantially different. Low certainty: our confidence in the effect estimate is limited: the true effect may be substantially different from the estimate of the effect. Very low certainty: we have very little confidence in the effect estimate: the true effect is likely to be substantially different from the estimate of effect.				
6.1.1.1 Explanations a. Heterogeneity I2 value is high (>90%). However, the difference in results can be linked to the different types of study population; the effect is higher in community-dwelling populations than in other clinical or nursing home populations.				

**Table 2:** Summary of findings table of any antipsychotic use on risk of cerebrovascular adverse events.

Summary of findings:			
Any antipsychotics compared to non-use for persons with dementia			
Patient or population: persons with dementia Setting: Community dwelling, nursing home, mixed Intervention: any antipsychotics Comparison: non-use			
Outcome № of participants (studies)	Relative effect (95% CI)	Certainty of the evidence (GRADE)	Comments
Risk of cerebrovascular adverse events (CVE) assessed with: Hazard ratio follow-up: mean 10.6 years № of participants: 72195 (5 non-randomized studies)	HR 1.77 (0.92 to 3.42) [Risk of cerebrovascular adverse events]	⊕⊕◯◯ Low^a,b^	The result is uncertain about the effect of antipsychotics use on risk of cerebrovascular adverse events.
*The risk in the intervention group (and its 95% confidence interval) is based on the assumed risk in the comparison group and the relative effect of the intervention (and its 95% CI). CI, confidence interval; HR, hazard ratio			
GRADE Working Group grades of evidence High certainty: we are very confident that the true effect lies close to that of the estimate of the effect. Moderate certainty: we are moderately confident in the effect estimate: the true effect is likely to be close to the estimate of the effect, but there is a possibility that it is substantially different. Low certainty: our confidence in the effect estimate is limited: the true effect may be substantially different from the estimate of the effect. Very low certainty: we have very little confidence in the effect estimate: the true effect is likely to be substantially different from the estimate of effect. Explanations a. Heterogeneity is high I2 value >90%. b. Funnel plot shows asymmetry.			

**Table 3:** Summary of findings table for typical antipsychotic use on risk of mortality in persons with dementia.

Summary of findings:				
Typical antipsychotics use compared to non-use for persons with dementia				
Patient or population: persons with dementia Setting: Nursing home, community dwelling Intervention: typical antipsychotics use Comparison: non-use				
Outcomes	Relative effect (95% CI)	№ of participants (studies)	Certainty of the evidence (GRADE)	Comments
Risk of mortality (Mortality risk) assessed with: hazard ratio follow-up: range 2 years to 12 years	HR 1.25 (1.01 to 1.55) [Risk of mortality]	77272 (6 non-randomized studies)	⊕⊕⊕⊕ High^a^	Typical antipsychotics use results in an increase in risk of mortality.
*The risk in the intervention group (and its 95% confidence interval) is based on the assumed risk in the comparison group and the relative effect of the intervention (and its 95% CI). CI, confidence interval; HR, hazard ratio				
GRADE Working Group grades of evidence High certainty: we are very confident that the true effect lies close to that of the estimate of the effect. Moderate certainty: we are moderately confident in the effect estimate: the true effect is likely to be close to the estimate of the effect, but there is a possibility that it is substantially different. Low certainty: our confidence in the effect estimate is limited: the true effect may be substantially different from the estimate of the effect. Very low certainty: we have very little confidence in the effect estimate: the true effect is likely to be substantially different from the estimate of effect.				

**Table 4:** Summary of findings for atypical antipsychotic use on risk of mortality in persons with dementia.

Summary of findings:				
Atypical antipsychotics use compared to non-use for persons with dementia				
Patient or population: persons with dementia Setting: Community dwelling, nursing home, mixed Intervention: atypical antipsychotics use Comparison: non-use				
Outcomes	Relative effect (95% CI)	№ of participants (studies)	Certainty of the evidence (GRADE)	Comments
Risk of mortality (Mortality risk) assessed with: hazard ratio follow-up: range 2 years to 12 years	HR 1.23 (1.05 to 1.43) [Risk of mortality]	83172 (7 non-randomized studies)	⊕⊕⊕⊕ High^a^	The evidence suggests atypical antipsychotics use results in an increase in risk of mortality.
*The risk in the intervention group (and its 95% confidence interval) is based on the assumed risk in the comparison group and the relative effect of the intervention (and its 95% CI). CI, confidence interval; HR, hazard ratio				
GRADE Working Group grades of evidence High certainty: we are very confident that the true effect lies close to that of the estimate of the effect. Moderate certainty: we are moderately confident in the effect estimate: the true effect is likely to be close to the estimate of the effect, but there is a possibility that it is substantially different. Low certainty: our confidence in the effect estimate is limited: the true effect may be substantially different from the estimate of the effect. Very low certainty: we have very little confidence in the effect estimate: the true effect is likely to be substantially different from the estimate of effect.				

## 4 Discussion

This systematic review synthesizes evidence on antipsychotic drug (APD) use in people with dementia (PwD). Across forty-five studies, we observed a consistent association between APD use and increased mortality risk, while evidence for other adverse outcomes was limited and highly heterogeneous. These findings highlight the need for cautious prescribing and regular treatment review in this vulnerable population.

Our meta-analysis reaffirms previous evidence that APD use is associated with elevated mortality risk in PwD. While associations have been consistently observed, causality cannot be inferred from existing evidence alone due to substantial heterogeneity (I^2^>90%), indicating a need for additional studies using designs that robustly address confounding and endogeneity. The heterogeneity underscores variation in study design, population characteristics, and exposure definitions, suggesting that more selective meta-analytic approaches—such as restricting analyses to studies with rigorous control of endogeneity and confounding variables or using higher thresholds for methodological quality—may yield more concise pooled estimates.

Endogeneity and confounding are significant challenges in evaluating the effect of APD use on mortality among PwD. Endogeneity arises because APD prescription is not random: clinicians tend to prescribe antipsychotics to patients with more severe behavioral symptoms, higher comorbidity, or greater frailty—characteristics that are themselves associated with increased mortality (84). This indication bias means that part of the observed association between APD use and mortality may reflect underlying patient characteristics rather than drug effects. Confounding further complicates causal inference: many included studies have not adequately measured or adjusted for factors such as functional status (51, 54, 55, 64, 70, 82), cognitive impairment (51, 55, 63, 69, 70), severity of neuropsychiatric symptoms (51, 52, 55, 63, 64, 70, 82), or coexisting medical conditions (51, 52, 55, 69, 70). For instance, claims or prescription databases rarely capture detailed clinical information, so their analyses may both underestimate and overestimate risk depending on which confounders are omitted—failing to adjust for frailty and cognitive decline tends to underestimate the true drug-mortality association, while ignoring behavioral severity can exaggerate harms (12, 57, 60, 61, 63, 66, 71). Within studies, bias may also result from exposure misclassification—patients may be classified as being exposed to antipsychotics based on prescription records, but actual consumption, adherence, and duration are often unknown (85). Thus, to advance knowledge, future research must use analytic strategies that limit confounding and indication bias, such as propensity score matching, instrumental variable analysis, or restricting to clinical subgroups where endogeneity is less pronounced. Rigorous adjustment is essential for valid estimation of APD effects on mortality in PwD.

However, our subgroup analyses showed increased risk for both typical and atypical APDs, with slightly higher hazard ratios in community-dwelling individuals compared to those in institutional care; however, the difference between subgroup estimates (e.g., hazard ratio 1.35 vs. 1.32) was narrow and unlikely to be clinically meaningful. Demographic and clinical factors also appear to influence mortality risk. Male sex and older age were associated with higher risk in most studies, although some findings were contradictory. Differences in baseline health status, dementia severity, and comorbidities may explain these inconsistencies.

Most studies were conducted in high-income countries and focused on older adults (≥65 years), limiting generalizability to younger PwD and those in low-resource settings. Several U.S. studies involved veteran populations, predominantly male, reducing applicability to non-veterans and females (62, 67).

Compared to randomized controlled trials (RCTs), observational designs offer advantages such as large sample sizes and long follow-up, but lack randomization and remain prone to selection bias, confounding by indication, and residual confounding. Additional residual confounding may arise from unmeasured variables such as BMI, cardiovascular risk factors, and lifestyle behaviors (e.g., smoking and alcohol use). Loss to follow-up was generally low, suggesting minimal attrition bias. Inconsistent outcome reporting limited meta-analysis for hospitalization and hip fracture. Future research should prioritize standardized reporting of time-to event outcomes (e.g., hazard ratios) to enable robust synthesis.

A key strength of this review is the inclusion of many studies, which enables a comprehensive synthesis and robust meta-analysis of mortality risk for both typical and atypical APDs. The application of PRISMA guidelines, GRADE methodology, and the use of dual bias assessment tools (Cochrane for randomized studies and NOS for observational studies) enhances methodological rigor and transparency. Subgroup analyses by drug type, study design, and care setting further improve the interpretability and clinical relevance of the findings. Importantly, this review’s inclusive approach—encompassing all types of dementia, a wide range of clinically important outcomes, and a broad spectrum of study designs—sets it apart from prior reviews that imposed narrower criteria.

However, such inclusiveness also introduces several limitations and limitations of a systematic review comes from the limitations of the primary studies. Including all dementia subtypes except Parkinson’s dementia, rather than focusing on specific forms like Alzheimer’s or Lewy body dementia, increases heterogeneity and may obscure subtype-specific effects, potentially limiting direct clinical applicability to particular patient groups. Similarly, the decision to synthesize a wide array of outcomes means that measures and definitions differ substantially across studies, complicating the interpretation and comparison of effects. Inclusion of diverse study designs also exposes the findings to varying levels of bias and methodological quality, which may limit the strength of causal inferences. Nevertheless, these limitations are balanced by the increased generalizability and breadth of evidence, which provide a more holistic view of APD use across the dementia population. Thus, the comprehensive scope of this review, while associated with interpretative challenges, can also be viewed as a methodological asset that distinguishes it from prior literature and augments its external validity.

Future studies should examine individual APDs with a focus on dose-response and treatment duration. Comprehensive assessment of BPSD severity, dementia stage, neuropsychiatric symptoms, and comorbidities as potential confounders or effect modifiers is essential. Broader outcomes—including hospitalization, long-term care admission, stroke, head injuries, pneumonia, quality of life, and healthcare costs—require further exploration, particularly in low- and middle-income countries where evidence is scarce.

## 5 Conclusion

Antipsychotic drug (APD) use in people with dementia (PwD) is consistently associated with an increased risk of mortality. Evidence for cerebrovascular events was attenuated but not statistically significant, while data on other outcomes remain limited and heterogeneous. These findings reinforce current regulatory warnings, including the FDA boxed warning, and underscore the need for cautious prescribing.

While APDs may provide benefits for managing severe BPSD, such as hallucinations, delusions, and dangerous agitation or psychosis, and can relieve suffering for both patients and caregivers, the cumulative evidence—including findings from this review—highlights that ADP initiation is associated with significant adverse outcomes. Clinical decision-making should therefore carefully balance potential benefits against known risks.

## Data Availability

The original contributions presented in the study are included in the article/**Supplementary Material**. Further inquiries can be directed to the corresponding author.
