# Supplementary material for: The consequences of antipsychotic medication use for people living with dementia: a systematic review and meta-analysis
Source: Front Psychiatry. 2026 May 8;17:1817609. doi: 10.3389/fpsyt.2026.1817609 (PMC13194443; doi:10.3389/fpsyt.2026.1817609)
Supplement: Supplementary file 1 [file SupplementaryFile1.docx]

Supplementary Material

# Supplementary Figures and Tables

## Supplementary Figures


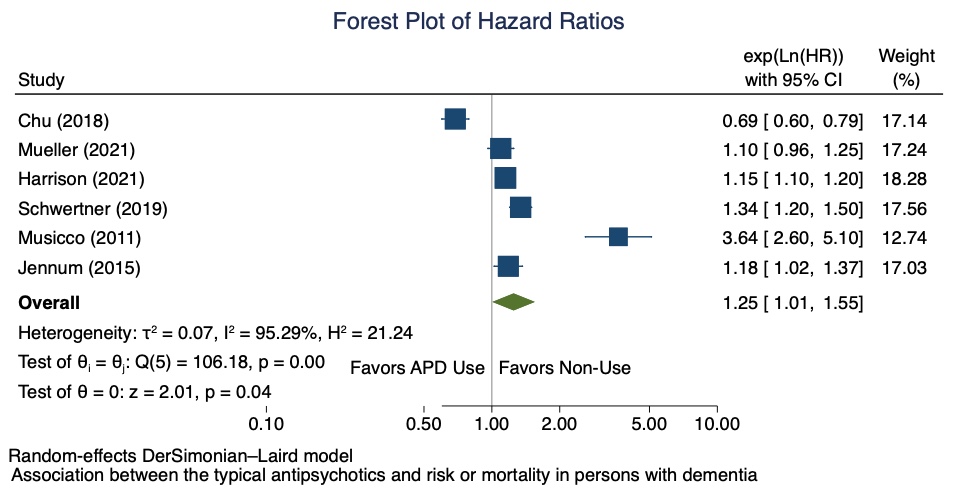


Supplementary Figure 1 Forest plot of the association between typical antipsychotic use and risk of mortality in persons with dementia


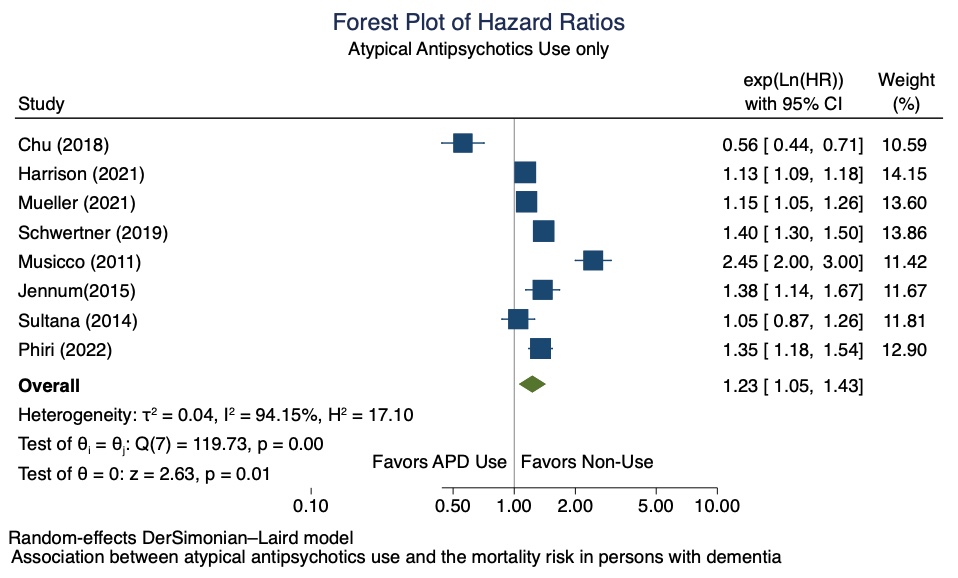


Supplementary Figure 2 Forest plot of the association between atypical antipsychotic use and risk of mortality in persons with dementia


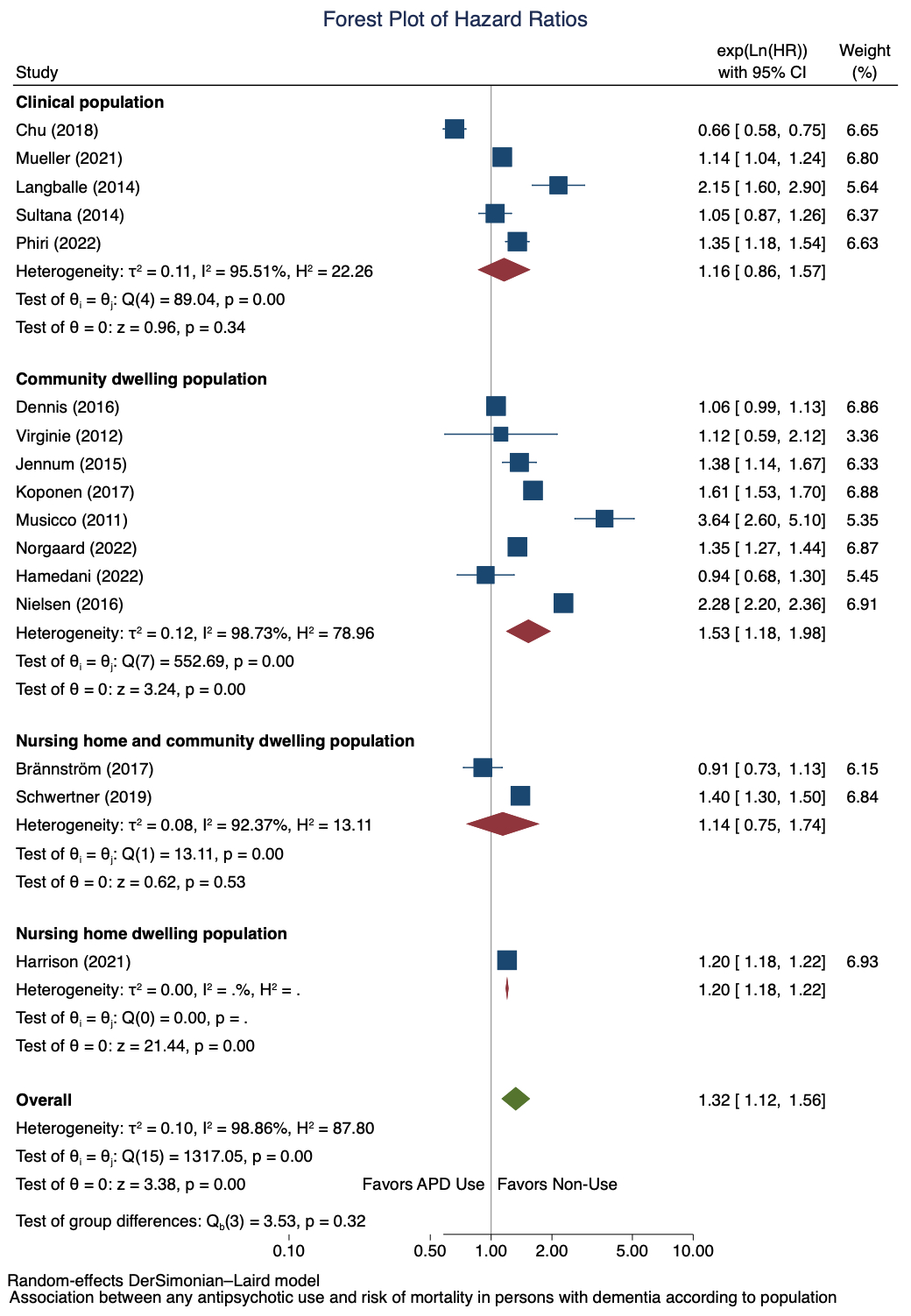


Supplementary Figure 3 Forest plot of the association between any antipsychotic use and risk of mortality among subgroups of different study population


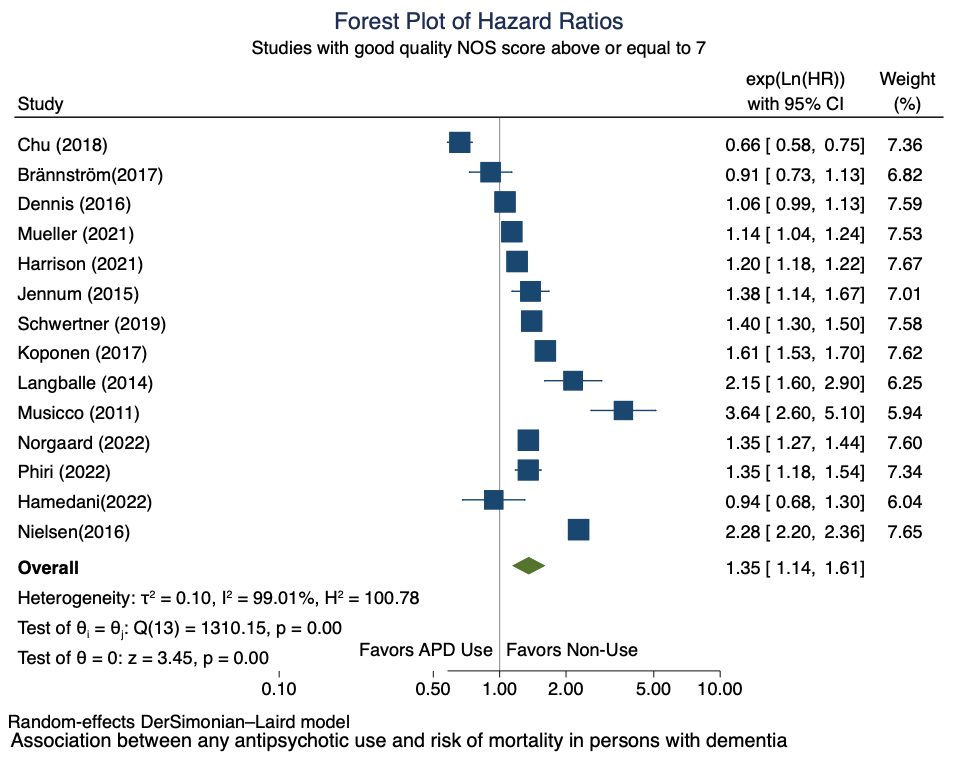


Supplementary Figure 4 Forest plot of the association between any antipsychotic use and risk of mortality in persons with dementia among studies with good quality NOS scores above or equal 7


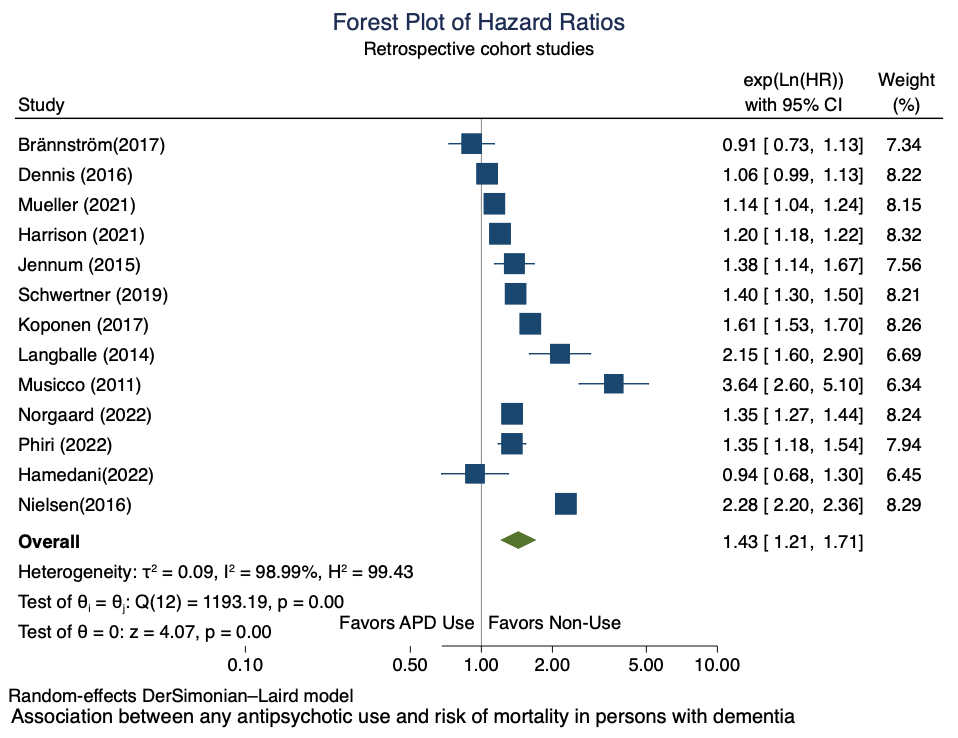


Supplementary Figure 5 Forest plot of the association between any antipsychotic use and risk of mortality in persons with dementia among retrospective cohort studies only


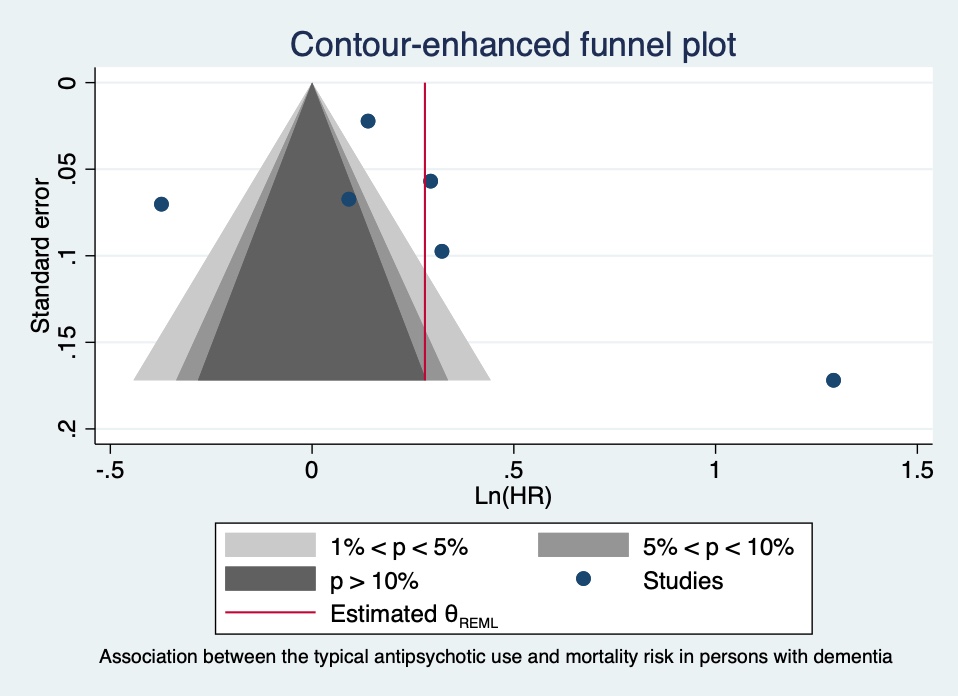


Supplementary Figure 6 Funnel plot of the studies on the association between the typical antipsychotic use and risk of mortality in persons with dementia


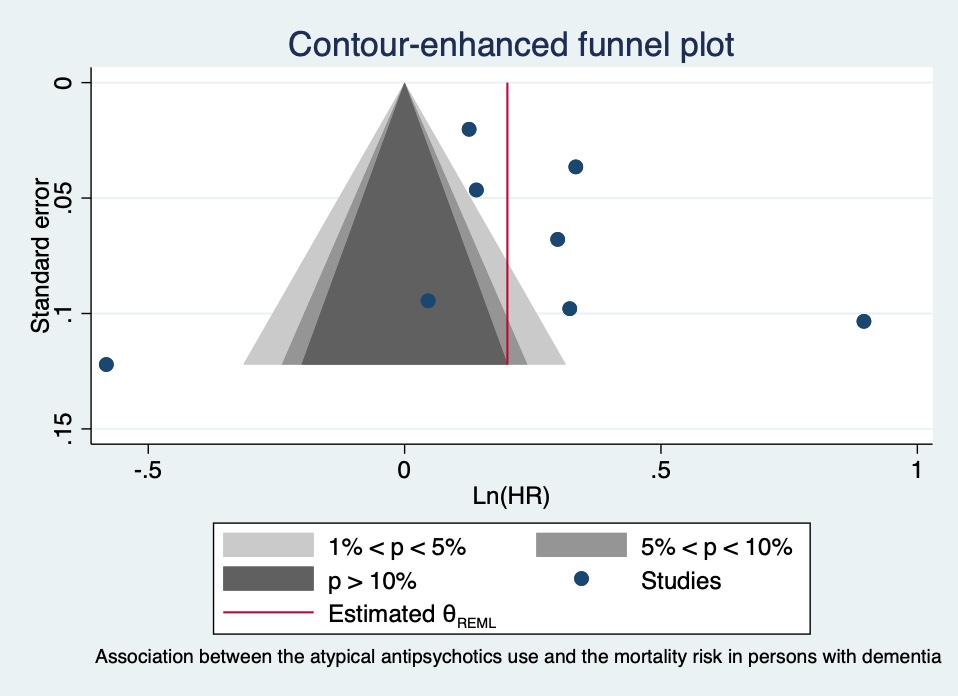


Supplementary Figure 7 Funnel plot of the studies on the association between atypical antipsychotic use and risk of mortality in persons with dementia

**1.2. Supplemenary tables**

Table 1 PECOS criteria

| Criteria | Inclusion | Exclusion |
| --- | --- | --- |
| Participants | People diagnosed with dementia (PwD) | People with Parkinson’s dementia |
| Exposure | Antipsychotics medication | Nothing specified |
| Comparison groups | No use of antipsychotics | Nothing specified |
| Outcomes | Any health outcome including but not limited to mortality and hospitalization | Non-health outcomes |
| Setting | All geographical location | Nothing specified |
| Other  - Study designs | Cohort, case-control, cross-sectional, and randomized controlled trials | Literature review, systematic review, meta-analysis, protocol, case report, and case series |
| Other- Language | English | Language other than English |

Table 2 Main characteristics and results of included studies presented as mentioned in the results section outcomes

| Author, year, and study setting | Target population, sample size | Sex and mean age | Type of Exposure and exposure measure | Dementia Diagnosis Method | Study design and duration | Outcome reported | Statistical analysis methods and confounders adjusted for |
| --- | --- | --- | --- | --- | --- | --- | --- |
| **Mortality** | | | | | | | |
| Schwertner, 2019 (1),  - Sweden | - dementia  - total sample size (n = 56,048)  - APD use (n = 2,526)  - typical APD use (n = 601)  - atypical APD use (n = 1,833)  - non-use (n = 53,522) | - 59% female  - mean age = 79.72 years | - typical: N05AA phenothiazines with aliphatic side-chain, N05AB phenothiazines with piperazine structure, N05AD phenothiazines with piperidine structure, N05AF thioxanthene derivatives;  - atypical: N05AE indole derivatives, N05AH diazepines, oxazepines, thiazepines, and oxepines, and N05AX other APDs. | Diagnosed via ICD-10 by clinicians and registered in SveDem + prescribed APDs | - registry-based cohort study  - 2007 to 2015 | All-cause mortality risk in persons with AD  Any APD use vs. non-use  **aHR = 1.38 (95% CI 1.3, 1.46)**  Typical APD vs non-use  **aHR = 1.35 (95% CI 1.2, 1.51)**  Atypical APD vs. non-use  **aHR = 1.39 (95% CI 1.29, 1.48)**  Typical vs. atypical APD-use  **aHR= 0.97 (95% CI 0.85, 1.11)** | - Cox proportional hazards model  - adjusted for age at dementia diagnosis, sex, Charlson comorbidity index, living arrangement, and MMSE |
| Koponen, 2017 (2),  - Finland | - community-dwellers with a clinically verified AD diagnosis during 2005-2011  - Total sample size (n= 57,755)  - APD monotherapy (n= 13,576)  - APD polypharmacy (n = 2,230)  - non- use (n= 41,949) | - 67.4 % female  - age in APD monotherapy  ≥ 85 years (20.6 %), 75-84 years (56.7%), <75 years (22.7 %)  - age in non- use  ≥ 85 years (24.3 %), 75-84 (55.6 %), <75 years (20.1 %) | - ATC class N05A excluding lithium (N05AN01) and 129 prochlorperazine (N05AB04)  - reimbursed prescription drug purchases during 1995-2012 from the Finnish Prescription register | Clinically verified AD | - register-based MEDALZ (Medication and Alzheimer’s disease) cohort study  - 2005 to 2011 | Non-cancer mortality risk  APD use vs. non-use  **aHR= 1.61 (95% CI 1.53,1.70)**  1-30 days of APD use vs. non-use  **aHR = 1.74 (95% CI 1.45, 2.10)**  31-90 days of APD use vs. non-use  **aHR = 2.11 ( 95% CI 1.85, 2.4)**  91-180 days of APD use vs. non-use  **aHR = 1.83 (95% CI 1.62, 2.08)**  181-365 days of APD use vs. non-use  **aHR = 1.64 (95% CI 1.47, 1.82)**  365-730 days of APD use vs. non-use  **aHR = 1.57 (95% CI 1.42, 1.74)**  >730 days of APD use vs. non-use  **aHR = 1.30 (95% CI 1.16, 1.46)** | - Cox proportional hazards model  - APD use as a time dependent variable  - adjusted for gender, age, Charlson Comorbidity Index score, and for history of stroke, hip fracture and ischemic cardiac events. Use of benzodiazepines, antidepressants and opioids were treated as time-dependent covariates |
| Dennis, 2017 (3),  - Wales | - dementia diagnosis at age ≥ 65y between 2003 and 2011  - total sample size (n = 9,674)  - APD use (n = 3,735)  - non-use (n = 5,939) | Exposed: 66.2% female; mean age at diagnosis of dementia = 82.4 (6.8) years Non-exposed: 67.8 % female; mean age at diagnosis of dementia: 82.5 (6.8) years | - typical and atypical APDs and other psychotrophic medication | SAIL databank, the date of first prescription after dementia diagnosis was recorded from GP computer records using NHS Read codes | - register based retrospective cohort study  - 2003 to 2011 | Mortality risk  All dementia types (n = 9,674)  **aHR 1.06 (95% CI 0.99, 1.13)**  Only Alzheimer’s dementia (n = 6,996)  **aHR 1.06 (95% CI 0.99, 1.14)**  Only known to primary care (n = 1122)  **aHR 1.03 (95% CI 0.96 , 1.10)**  Typical APD vs. atypical APD users (exclusion of double users)  **aHR = 0.94 (95% CI 0.8, 1.03)** | - Cox proportional hazards model  - age, gender, co-morbidities (epilepsy, parkinsonism, atrial fibrillation, venous thromboembolism, diabetes, ischemic heart disease, cerebrovascular disease, hip fracture), use of hypnotics, anxiolytics and benzodiazepines |
| Mueller, 2020 (4),  - the UK | - persons with dementia (excluding Lewy-body dementia)  - total sample size (n = 10,106)  - any APD (n = 1,115)  - non-use (n = 8,991)  - any typical APD (n = 386)  - any atypical APD (n = 889) | - 63.2 % female - mean age at dementia diagnosis= 81.1 years | - any drug listed in chapter 4.2 of the British National Formulary (including 4.2.1 antipsychotic drugs, 4.2.2 antipsychotic depot injections, 4.2.3 drugs used for mania and hypomania (arsenapine, lithium carbonate, lithium citrate, valproic acid) | SLaM Database | - cohort study  - 1 January 2007 to 31st December 2015 | All-cause mortality risk  any APD vs. non- use  **aHR = 1.14 (95% CI 1.04, 1.24)**  any Typical APD vs. non-use  **aHR = 1.10 (95 % CI 0.96, 1.25)**  any Atypical APD vs. non-use  **aHR = 1.15 (95% CI 1.05, 1.26)**  any Typical APD vs. any Atypical APD  **aHR = 0.95 (95 % CI 0.79, 1.14)**  Risperidone (n = 283) vs. any other APD (n=832)  aHR = 1.03 (95 % CI 0.86, 1.22)  Risperidone (n = 283) vs. no APD-use  aHR = 1.16 (95% CI 0.99, 1.36)  Olanzapine (n = 162) vs. any other APD (953)  aHR = 1.02 (95 % CI 0.81, 1.29)  Olanzapine (n = 162) vs. no APD use  aHR = 1.16 (95% CI 0.93, 1.45)  Quetiapine (n = 402) vs. any other APD (n = 713)  aHR = 0.95 (95% CI 0.82,1.10)  Quetiapine (n = 402) vs. no APD use  aHR = 1.11 (95 % CI 0.98, 1.25) | - Cox proportional hazards model  - adjusted for age, gender, marital status, ethnicity, index of deprivation, MMSE score, dementia subtype, HoNOS scores (agitation, psychosis, non-accidental self-injury, problem-drinking or drug taking, depressed mood, physical illness or disability, ADL, living conditions, occupational/recreational activities, social relationships), and hospitalization in the year prior to dementia diagnosis |
| Mueller, 2020 (4),  - the UK | - persons with dementia (excluding Lewy-body dementia)  - total sample size (n = 10,106)  - any APD (n = 1,115)  - non-use (n = 8,991)  - any typical APD (n = 386)  - any atypical APD (n = 889) | - 63.2 % female - mean age at dementia diagnosis= 81.1 years | - any drug listed in chapter 4.2 of the British National Formulary (including 4.2.1 antipsychotic drugs, 4.2.2 antipsychotic depot injections, 4.2.3 drugs used for mania and hypomania (arsenapine, lithium carbonate, lithium citrate, valproic acid) | SLaM Database | - cohort study  - 1 January 2007 to 31st December 2015 | Stroke-specific mortality risk  any APD vs. non- use  **aHR = 1.28 (95% CI 1.01, 1.63)**  any Typical APD vs. non-use  aHR = 1.22 (95 % CI 0.86, 1.73)  any Atypical APD vs. non-use  **aHR = 1.31 (95% CI 1.00, 1.70)**  any Typical APD vs. any Atypical APD  aHR = 0.84 (95 % CI 0.49, 1.44)  Risperidone (n=283) vs. any other APD (n=832)  aHR = 1.07 (95 % CI 0.65, 1.75)  Risperidone (n = 283) vs. no APD-use  aHR = 1.34 (95% CI 0.85, 2.11)  Olanzapine (n = 162) vs. any other APD (953)  aHR = 1.27 (95 % CI 0.68, 2.35)  Olanzapine (n = 162) vs. no APD use  aHR = 1.58 (95% CI 0.88, 2.83)  Quetiapine (n = 402) vs. any other APD (n = 713)  aHR = 0.92 (95% CI 0.60, 1.41)  Quetiapine (n = 402) vs. no APD use  aHR = 1.22 (95 % CI 0.86, 1.73) | - Cox proportional hazards model  - adjusted for age, gender, marital status, ethnicity, index of deprivation, MMSE score, dementia subtype, HoNOS scores (agitation, psychosis, non-accidental self-injury, problem-drinking or drug taking, depressed mood, physical illness or disability, ADL, living conditions, occupational/recreational activities, social relationships), and hospitalization in the year prior to dementia diagnosis |
| Virginie 2012 (5),  - France | - community dwelling Alzheimer's disease patients (AD) looked after by an informal caregiver  - Total sample size (n = 534),  - Typical APD users (n = 58),  - Atypical APD users (n = 44)  - non-use (n = 432) | - 72.3 % female  - mean age = 77.7 (6.8) years | -APDs as the ATC N05A class (excluding lithium)  -risperidone, olanzapine, and aripiprazole were considered as second-generation APs (quetiapine and ziprasidone were not commercialized in France during the study period and no subject was exposed to amisulpride nor clozapine) | DSM-IV/NINCDS-ADRDA, MMSE 10–26 | - multicentric prospective cohort study  - 2000 and 2002 and followed-up for 4 years | 3.5-year all-cause mortality risk  APD use vs. non-use  aHR= 1.12 (95% CI 0.59, 2.12) | - Cox proportional hazards model  - APD use as a time-dependent variable  - adjusted for age, gender and center (non-proportionality stratification), time-fixed variables: history of diabetes, history of cardiovascular disease; and time-dependent variables: MMSE score, ADL score, NPI psychosis sub score, NPI hyperactivity sub score, MNA score (dichotomized using a cutoff of 23.5), medical assistance, and hospitalization) |
| Norgaard, 2022 (6),  - Denmark | - persons aged 65–95 years who received a dementia diagnosis between 2009 and 2014  - total sample size included (n=32,974) (8,244 exposed / 24,730 unexposed) | Both APD users and non-users:  - 56.70% female  - median age = 81.7 (IQR 76.2-86.4) | Antipsychotics (ATC N05A excl. lithium) | Via registry: persons with first-time dementia diagnosis and/or first antidementia drug prescription | - register-based matched cohort study  - 2009 to 2014 | 180-day mortality risk  **aHR = 1.35 (95% CI 1.27, 1.43)** | - 1:3 matched non-APD-users on sex, age at time of dementia diagnosis (+/- 1 year), calendar year of first diagnosis (+/- 1 year)  - Cox proportional hazards model  - age at dementia diagnosis, sex, calendar year, nursing home residency, heart disease, cerebrovascular disease, diabetes mellitus, Charlson Comorbidity Index score (excluding dementia, acute myocardial infarction, congestive heart disease, cerebrovascular disease, diabetes, and diabetes with end-organ damage), psychiatric comorbidity, treatment with benzodiazepines and antidepressants within 180 days before the index date, and number of hospitalizations within 5 years before the index date |
| Brännström, 2017 (7),  - Sweden and Finland | - persons with dementia aged ≥ 65 years  - total sample size (n =1,037)  - APD use (n = 230)  - non-use (n = 807) | - 74% female  - mean age = 89.4 (6.2) years | Any APDs:  N05A (excl. lithium), hydroxyzine (N05BB01), propiomazine (N05CM06), and phenothiazine derivates (R06AD)  - antidepressant drugs: all drugs within ATC code N06A  - benzodiazepines, including benzodiazepine- like Z-drugs (N05BA, N05CD, N05CF, and N03AE) | DSM-IV | - merged prospective cohort from studies in the region of Västerbotten, Sweden and Finnland: GERDA, 2000–2012; FOPANU, 2002; REMANU, 2004); (UMDEX, 2012)  - 2000 to 2012 | 2-years mortality risk  APD use vs. non-use  aHR = 0.91 (95% CI 0.73, 1.14)  Male participants  aHR = 0.79 (95% CI 0.51, 1.24)  Female participants  aHR = 0.90 (95% CI 0.7, 1.15) | - Cox proportional hazards model  - interaction term (sex*antipsychotics)  - sex, age, delirium in the last month, myocardial infarction, chronic lung disease, prescribed drugs (APDs, benzodiazepines, organic nitrates, number of prescribed drugs, Barthel ADL index, GDS-15, MMSE |
| Hamedani, 2022 (8),  - USA | - dementia patients ≥ 65 years  - total sample size (n = 1,703)  - exposed sample (n=284) | - 66.10% female  - age:  ≤ 69- 2.9% 60-69- 23.2% ≥ 80 - 73.9% | Typical and atypical antipsychotics (except pimavaserin) | Dementia defined by NHATS and HRS surveys (based on an algorithms predicting dementia probability via self-reported items) | - Retrospective cohort study (Medicare claims from National Health and Aging Trends Study (NHATS) and Health and Retirement study (HRS)) | Mortality risk  APD-use versus non-use  aHR = 0.94 (95% CI 0.68, 1.28) | - Cox proportional hazards model with time-varying coefficients for hallucinations, antipsychotic use, ADL/iADL limitations, multimorbidity  - adjusted for hallucinations, age, race, gender, smoking, metropolitan residence, marital status, nursing home residence, multimorbidity index, visual impairment, ADL limitations, iADL limitations, and baseline PD/DLB |
| Chu, 2018 (9) ,  - Taiwan | - ≥ 65 years, diagnosed with AD between January 2001 and December 2006  - Total sample size (n = 2,169),  selected with APD use (n = 735), selected with no use of AP (n = 735)  - Typical APD (yes/no, n = 574/896)  - Atypical APD (yes/no, n = 161/1309) | - exposed: 49.3% female, non-exposed: 47.1%  - mean age exposed = 78.3±6.6 years, non-exposed = 78.3±6.8 years | - typical and atypical APDs | ICD-9-CM codes: 290.0–  290.3, 294.1–294.2, 331.0 AND minimum two outpatient visits or inpatient hospital record | - retrospective cohort study  - 1 January 2001 to 31 December 2011 | Mortality risk  APD use vs. non-use  **aHR = 0.66 (95% CI 0.58, 0.75)**  Typical APD use vs. non-use  **aHR = 0.69 (95% CI 0.60, 0.79)**  Atypical APD use vs. non-use  **aHR = 0.56 (95% CI 0.44, 0.71)**  Cumulative APs DDD  No use = reference  <60 **aHR 0.68 (95% CI 0.59, 0.77)**  60-180 **aHR 0.58 (95% CI 0.37, 0.93)**  >180 **aHR 0.37 (95% CI 0.20, 0.68)**  Cumulative APs period  No use = reference  <60 days  **aHR = 0.68 (95% CI 0.58, 0.78)**  60-180 days  **aHR = 0.67 (95% CI 0.53, 0.85)**  >180 days  **aHR = 0.37 (95% CI 0.20, 0.68)** | - Cox proportional hazards model  - 1:1 propensity score matching via age, sex, physical conditions as measured using the Charlson Comorbidity Index (CCI), and entry year  - adjusted for age, gender, cholinesterase inhibitors, diabetes mellitus, hypertension, hyperlipidemia, conjestive heart failure, major depression, stroke, delirium; Model 2 same adjustments (TAP yes/no and AAP yes/no); Model 3 same adjustments (No. of DDD AP used); Model 4 same  - additional analysis with APs as time-dependent covariates, as well as subgroup analyses for TAP and AAP |
| Harrison, 2021 (10),  - Australia | - aged 65 or older OR aged 50 years or older if Aboriginal or Torres Strait Islander in residential aged care facility, new users of APD  - persons with dementia included (n = 119,665)  - APD use (n = 22,408)  - non-use (n = 92,257)  - typical APD use (n = not shown)  - atypical APD use (n = not shown) | - 65% female  - mean age= 84.4years | typical APDs: ATC codes N05AA-N05AD, (excluding N05AB04, and N05AF)  atypical APDs: N05AE, N05AH, N05AL and N05AX  - APDs use within 100 days after moving to a residential aged care facility | Aged care assessments + dispensing of dementia-related medications (e.g., AChE inhibitors, memantine) | - retrospective cohort study  - 1 April 2008 to 30 June 2015 | All-cause mortality risk  APD use vs. non-use  **aHR = 1.20 (95% CI 1.18, 1.22)**  Typical APD vs. non-use  **aHR = 1.14 (95 % CI 1.10, 1.20)**  Atypical APD vs. non-use  **aHR = 1.17 (95 % CI 1.15, 1.17)**  Typical vs. atypical APD use  aHR = 1.02 (95 % CI 0.97, 1.07) | - Cox proportional hazards model  - Adjusted for age, sex, comorbidity score and activity limitations, verbal and physical behavioural symptoms, wandering, depression and cognitive impairment |
| Harrison, 2021 (10),  - Australia | - aged 65 or older OR aged 50 years or older if Aboriginal or Torres Strait Islander in residential aged care facility, new users of APD  - persons with dementia included (n = 119,665)  - APD use (n = 22,408)  - non-use (n = 92,257)  - typical APD use (n = not shown)  - atypical APD use (n = not shown) | - 65% female  - mean age= 84.4years | typical APDs: ATC codes N05AA-N05AD, (excluding N05AB04, and N05AF)  atypical APDs: N05AE, N05AH, N05AL and N05AX  - APDs use within 100 days after moving to a residential aged care facility | Aged care assessments + dispensing of dementia-related medications (e.g., AChE inhibitors, memantine) | - retrospective cohort study  - 1 April 2008 to 30 June 2015 | All-cause mortality risk  APD use vs. non-use  **aHR = 1.15 (95% CI 1.10, 1.20)**  Typical APD vs. non-use  **aHR = 1.16 (95 % CI 1.06, 1.27)**  Atypical APD vs. non-use  **aHR = 1.13 (95 % CI 1.09, 1.18)**  Typical vs. atypical APD use  aHR = 1.06 (95 % CI 0.96, 1.17) | - Cox proportional hazards model  - Adjusted for age, sex, comorbidity score and activity limitations, verbal and physical behavioural symptoms, wandering, depression and cognitive impairment |
| Jennum, 2015 (11),  - Denmark | - diagnosis of dementia between 1997 and 2009  - total sample (n = 71,107)  - PwD (n = 26,821),  - typical APD in PwD (n = 259),  - atypical APD in PwD (n = 832),  - non-use in PwD (n = 8,631) | patients with dementia  - 59.7% female,  - mean age = 79.1 | - first generation APDs (Typical APD) and second generation anti- psychotics (Atypical APD)  - non-users were excluded if they were medicated with selective serotonin reuptake inhibitors (SNRIs), serotonin-noradrenaline re-uptake inhibitors (SSRIs), tricyclic antidepressants (TCAs), benzodiazepines (BZDs), benzodiazepine-like drugs (BZD-like) | ICD‑10 codes F00–F03 in National Patient Registry | - a population-based register study  - 1997 and 2009 | All-cause mortality risk  Typical APD vs. non-use  **HR = 1.183, p = 0.022** (95% CI not reported)  Atypical APD vs. non-use  **HR = 1.380, p < 0.001** (95% CI not reported) | - Cox proportional hazards model  ~~-~~- adjustment with covariates not clearly stated |
| Musicco, 2011, (12)  - Italy | - 60 years or older who were newly prescribed an acetylcholinesterase inhibitor between Jan 2002 and June 2008, living in Milan  - total sample size (n = 4,369)  - typical APD use (n = 136),  - atypical APD use (n = 136),  - typical and atypical APD use (n =103)  - non-use (n = 3,276) | total population (65% female, mean age= 78.5 (6.6) years)  typical users (69.1% female, mean age= 79.2 (6.1) years)  atypical users (63.7% female, mean age= 78.7 (6.4) years)  typical and atypical users (54.4% female, mean age = 76.8 (6.6)  non-users (65.5% female, mean age= 78.5 (6.7) years) | atypical APD  risperidone, olanzapine, quetiapine, and clozapine conventional APD  haloperidol, thioridazine, clotiapine, chlorpromazine, trifluoperazine, levomepromazine, amisulpride, periciazine, fluphenazine, pimozide, tiapride, levosulpiride, bromperidol, dixyrazine, zuclopentixol, sulpiride, pipamperone, and perphenazine | Based on first prescription of acetylcholinesterase inhibitors (donepezil, rivastigmine, galantamine) | - retrospective population cohort study  - follow-up two years after prescription of acetylcholinesterase inhibitor | 2- years risk of death  Typical APD vs. non-use  **aHR = 3.7 (95% CI 2.6, 5.1)**  Atypical APD vs. non-use  **aHR = 2.5 (95% CI 2.0, 3.0)**,  Typical vs. atypical APD use  **aHR = 1.5 (95% CI 1.1, 2.1)** | - Cox proportional hazards model  - APD use as a time-dependent variable  - age, sex, comorbidities (modified Elixhauser comorbidity), and propensity score of being prescribed an APD drug |
| Nerius, 2018 (13),  - Germany | - persons with dementia ≥ 60 years newly-diagnosed between January 2006 and December 2010  - total sample size (n = 27,801)  - total sample size outcome death (n = 10,921) | - 16600 person years (Women), 7911 person years (Men) | - haloperidol, melperone, risperidone, quetiapine, other typical apd (=FGA), other atypical apd (=SGA)  other typical apd include all APD (ATC = N05A) except the APD  mentioned above and other atypical APD which are amisulpride, zotepine, ziprasidone, aripiprazole, sertindole, olanzapine, and clozapine | ICD-10 codes G30, G31.0, G31.82, G23.1, F00, F01, F02, F03, and F05.1 in administrative claims data of AOK (Germany) | - cohort study  - January 2006 and December 2010 | Mortality risk  Haloperidol vs. non-Haloperidol-use  **aHR = 1.56 (95% CI 1.38, 1.75),**  Melperone vs. non-Melperone-use  **aHR = 1.43 (95% CI 1.33, 1.54)**,  Risperidone vs. non- Risperidone-use **aHR = 1.28 (95% CI 1.17, 1.40)**,  Quetiapine vs. non-Quetiapine-use  aHR = 0.91 (95% CI 0.78, 1.08),  Other typical APDs vs non-use  **aHR = 1.26 (95% CI 1.14, 1.39),**  Other atypical APDs vs non-use  aHR = 1.09 (95% CI 0.80, 1.49). | - Cox proportional hazards model  - sex, age, comorbidities, polypharmacy, antidementia drug use, LTC dependency, residency |
| Sultana, 2014 (14),  - the UK | - vascular dementia (VaD) diagnosis between January 2007 and December 2010, ≥ 30 years old  - total sample size (n = 1,531)  - APD use (n = 286)  - non-use (n = 1,245)  - risperidone (n = 81)  - non-exposed to risperidone (n = 1,450)  - quetiapine (n = 21)  - non-exposed to quetiapine (n= 1 ,314) | - 57.7% female  - mean age at diagnosis of vascular dementia = 78.95 years (survivors), 83.38 years (persons deceased during follow-up) | - olanzapine, quetiapine, risperidone, haloperidol | ICD-10 or diagnosis in free text of medical register | - clinical cohort study (SLaM database)  - maximum 5 year follow-up | Mortality risk  Any APD vs. Non-use  **aHR = 1.05 (95% CI 0.87, 1.26),**  Risperidone vs. no Risperidone use  aHR = 0.87 (95% CI 0.60, 1.27),  Quetiapine vs. no quetiapine use  aHR = 1.13 (95% CI 0.92, 1.37). | - Cox proportional hazards model  - age at diagnosis of vascular dementia, gender, ethnicity, cognitive function measured by MMSE |
| Kales, 2012 (15),  - the US | - aged ≥ 65 years old with a dementia diagnosis between 1^st^ October 1998 to 30^th^ September 2008  - total sample size (n = 33,604) | Values for Haloperidol, olanzapine, quetiapine and risperidone users, respectively:  - female: 2.3%, 2.8%, 2.1%, 2.8%  - percent of total sample per age group for  65-69y: 4.8%, 6.1%, 5.2%, 5.7%;  70-74y: 12.9%, 13,7%, 14.9%, 13,7%;  75-79y: 26.2%, 27.6%, 27.1%, 28.1%;  80-84y: 33.2%, 33.3%, 32.7%, 32.5%;  85+y: 22.8%, 19.2%, 20.0%, 20.0% | - risperidone, olanzapine, quetiapine, and haloperidol, valporic acid and derivatives  - exposure-days were the length of time from the first filled prescription until death or 6 months, whichever was earlier | ICD 9 diagnoses 290.0, 290.1x, 290.2x, 290.3, 290.4x, 291.2, 294.10, 294.11, 331.0, 331.1, and 331.82 | - retrospective cohort study  - national Veteran’s association registry  - 1 October 1998 to 30 September 2008 | 180-day mortality risk  Haloperidol (n=2855) vs. risperidone (n=13356) use  **aHR = 1.54 (95% CI 1.38, 1.73),**  Olanzapine (n=4716) vs. risperidone (n=13356) use  aHR = 0.99 (95% CI 0.89, 1.10),  Quetiapine (n=10651) vs. risperidone (n=13,356) use  **aHR = 0.73 (95% CI 0.67, 0.80).** | - Cox proportional hazards model  - gender, age, race, marital status, delirium, depression, schizophrenia, bipolar I, bipolar II, other psychoses, Parkinson’s disease, substance abuse, PTSD, other anxiety, personality disorder, use of benzodiazepine, antidepressant, opioid, days in hospitalization, days in nursing home, fiscal year of index drug use, rurality of facility, facility size, academic affiliation of facility, Charlson’s comorbidity index, myocardial infarction, congestive heart failure, peripheral vascular disease, cerebrovascular disease, COPD, rheumatologic disease, peptic ulcer disease, cirrhosis, hepatic failure, diabetes mellitus, diabetes mellitus with complications, hemiplegia, chronic renal disease, malignant neoplasm, leukemia, lymphomas, metastatic solid tumor, and AIDS |
| Maust, 2015 (16),  - the US | - 65 years or older with a diagnosis of dementia between October 1998 and September 2009  - total sample size (n = 90,786)  - APD use (n = 46,008) | - 1.8-2.9 % female across APD user groups  - age distribution across APD user groups  65-69 years (4.7- 9%)  70-74 years (12.9 - 15.0%)  75-79 years (25.9 - 27.2%)  80-84 years (28.6 - 32.8 %)  ≥85 years (19.5 - 23.8 %) | - APD monotherapy (haloperidol, olanzapine, quetiapine, and risperidone), anticonvulsant valproic acid and its derivates, Antidepressants excl. tricyclic or MAO | ICD-9 codes 290.0, 290.1x, 290.2x, 290.3, 290.4x, 291.2, 294.10, 294.11, 331.0, 331.1, and 331.82 | - retrospective cohort study  - October 1, 1998, through September 30, 2009 | 180-day mortality risk difference  haloperidol (n = 1,958) vs. non-users  **3.8% (95% CI 1.0, 6.6)**  **NNH of 26 (95% CI 15, 99)**  Risperidone (n = 6,471) vs. non-users  **3.7% (95% CI 2.2, 5.3)**  **NNH of 27 (95% CI 19, 46)**  Olanzapine (n = 1,952) vs non-users  **2.5% (95% CI 0.3, 4.7%)**  **NNH of 40 (95% CI 21, 312)**  Quetiapine (n = 4,700) vs. non-users  **2.0% (95% CI 0.7, 3.3)**  **NNH of 50 (95% CI 30, 150)** | - APD users and non-users matched by calendar year of diagnosis, age (±2,5 years), race, delirium diagnosis within preceding 12 months, psychiatric hospitalization within preceding 12 months, 3-Category Comorbidity Index score  - generalized linear model with logit link to fit a logistic regression model for 180-day mortality risk associated with given medication  - pairing was accounted for by a generalized estimating equation and adjusted for x (table 2)  - adjusted for sex, centered age and its quadratic term, marital status, depression, schizophrenia, bipolar I disorder, bipolar II disorder, other psychoses, Parkinson disease, substance abuse, posttraumatic stress disorder, other anxiety disorders, personality disorder, use of benzodiazepines, use of opioids, days of hospitalization, days in nursing home, fiscal year of index drug use, academic affiliation of facility, myocardial infarction, congestive heart failure, peripheral vascular disease, cerebrovascular disease, chronic obstructive pulmonary disease, rheumatologic disease, peptic ulcer disease, cirrhosis, hepatic failure, type 1 or 2 diabetes mellitus, type 1 or 2 diabetes mellitus with complications, hemiplegia, chronic renal disease, malignant neoplasm, leukemia, lymphoma, metastatic solid tumor, human immunodeficiency virus without AIDS, and AIDS. |
| Phiri, 2022 (17),  - UK | - Dementia patients > 30 years  - 1,480 (587 medicated/ 893 controls) | - 51.80% female  - mean age = 82.6 ± 8.1 | Olanzapine, quetiapine, risperidone | Clinical records within Southern Health NHS Foundation Trust (SHFT) (diagnosis codes) | -Retrospective clinical cohort  - 2013 to 2017 | Mortality risk  Olanzapine vs. non-APD use  **aHR = 1.32 (95% CI 1.08, 1.60)**,  Risperidone vs. non-APD use  **aHR = 1.35 (95% CI 1.18, 1.54)**,  Quetiapine vs. non-APD use  **aHR = 1.09 (95% CI 0.90, 1.34).** | - Cox proportional hazards model  - adjusted for age, gender, ethnicity, MMSE score |
| Langballe, 2014 (18),  - Norway | - 65 years of age or older who had received at least one prescription of antidementia drugs (ATC code N06D) memantine, donepezil, rivastigmine, or galantamine at a Norwegian pharmacy  - total sample size (n = 26,940)  - APD use (n = 8,214)  - other psychotropics use (n = 18,726) | - APDs use  - % female- not stated  - mean age= 80.6 years  other psychotropics use  - % female- not stated  - mean age= 79.4 years | - APDs group (ATC code N05A, excluding lithium): chlorpromazine, levomepromazine, chlorprothixen, fluphenazine, perfenazine, prochlorperazine, thioridazine, zuclo- penthixol, haloperidol, flupentixol, sertindol, ziprasidone, clozapine, olanzapine, quetiapine, asenapine, risperidone, aripiprazol, paliperidone, and amisulpride.  - reference group- antide- pressants (N06A), benzodiazepines (N03AE01, N05B, N05C), benzodiazepine-like agents (N05C), lithium (N05AN01), and anticonvulsive drugs (N03A) | Dementia diagnosis via prescriptions of antidementia drugs | - population-based study  - 2004 to 2010 | Mortality risk  APDs vs. other psychotropic drugs  For 0-30 days  **aHR = 2.1 (95% CI 1.6, 2.9),**  30-180 days  **aHR = 1.7 (95% CI 1.4, 2.1),**  180-365 days  **aHR = 2.5 (95% CI 2.0, 3.1),**  365 – 730 days  **aHR = 2.5 (95% CI 2.1, 2.9),**  For 730-2400 days  **aHR = 1.7 (95% CI 1.6, 1.9),**  Individual APDs  For 0-30 days  Haloperidol vs. Risperidone  **aHR =** **1.7 (95% CI 1.0, 3.0),**  Quetiapine vs. Risperidone  aHR = 1**.**3 (95% CI 0.6, 2.9),  For 730-2400 days  Haloperidol vs. Risperidone  **aHR =** **1.4 (95% CI 1.0, 1.9),**  quetiapine vs. risperidone  **aHR =** **1.4 (95% CI 1.0, 1.9).** | - Cox proportional hazards model  - fixed effects: age, gender, mean daily defined dosage (DDD) of psychotropics, and prescription of drugs for treatment of cancer, diabetes, cardiovascular diseases, obstructive lung disease, and musculoskeletal diseases |
| Nielsen, 2016 (19),  - Denmark | - AD  - Total sample size (n = 45,894) | - 63 % females  - age not reported | - APDs were defined as ATC N05A, excluding lithium (ATC N05AN).  - current exposure- prescription for an APD drug.  - the cumulative APD dosages from dementia diagnosis until end of study for each participant were calculated and divided into groups | ICD-10 F00.x or G30.x) or patients treated with antidementia drugs (ATC) code N06D) | - nationwide, population-based, retrospective cohort study design  - 3 803 996 person-years  - ICD-10 F00x (Dementia in AD) from January 1, 2000 to December 31, 2011 | Mortality risk  Current exposure vs. no exposure  **aHR =** **2.28 (95% CI 2.20, 2.35**)  Cumulative APD exposure > 0 Daily Defined Dosage (DDDs) but < 90  **aHR =** **2.20 (95% CI 2.14, 2.27**),  ≥ 90 DDDs but < 365  **aHR =** **1.81 (95% CI 1.74, 1.89),**  ≥ 365 DDDs but < 730  **aHR = 1.38 (95% CI 1.428, 1.49),**  ≥ 730 DDDs  aHR = 1.06 (95% CI 0.95, 1.18). | - Cox proportional hazards model  - severity of AD, psychiatric comorbidity, somatic comorbidity, and cardiovascular risk factors |
| Rossom, 2010 (20),  - the US | - ≥65 years, with a diagnosis of dementia between Oct 1999 and Sept 2005 and no other indication for an APD,  (n = 18,127) | - 2 -3 % female  - mean age 77-78 years | - haloperidol, olanzapine, quetiapine, risperidone  - the initially prescribed daily doses of each APD - multiplying the total number of doses dispensed times their strength in milligrams and dividing by the days’ supply recorded on the electronic prescription re- cord | Dementia diagnosis from veterans healthcare data (ICD-9 codes 290, 290.0, 290.1 to 290.3, 290.4, 290.9, 291.1 to 291.2, 294.1, 331.0, 331.1, 331.82) | -retrospective cohort study  - October 1999 to September 2005 | Mortality risk of first 30 days after initial prescription  Haloperidol vs. non-use  **aHR = 2.2 (95% CI 1.7, 2.9),**  Olanzapine vs. non-use  **aHR =** **1.3 (95% CI 1.0, 1.7)**,  Risperidone vs. non-use  **aHR =** **1.2 (95% CI 1.0, 1.4),**  Quetiapine vs. non-use  aHR = 0.8 (95% CI 0.6, 1.1). | - Cox proportional hazards model of the natural logarithm of HR  - age, sex, type of dementia, and history of cerebrovascular disease, ischemic heart disease, peripheral vascular disease. heart failure, cancer, autoimmune deficiency syndrome, diabetes mellitus, chronic obstructive pulmonary disease, liver disease, renal disease, para- or hemiplegia, rheumatic disease, or peptic ulcer disease, outpatient prescriptions (achetylcholinesterase inhibitors, alpha-adrenergic receptor blockers, angiotensin-converting enzyme inhibitors or angiotension receptor blockers, anti-arrhythmics, antibiotics, anticonvulsants, antidepressants, antiplatelet agents, beta-adrenergic receptor blockers, benzodiazepines, calcium-channel blockers, cancer chemotherapy, digoxin, diuretics, medications for diabetes mellitus, hyperlipidemia, Parkinson's disease, peptic ulcer disease, or pulmonary disease, memantine, nitrates, non-steroidal anti-inflammatory agents, opiates, other analgesics, vitamin E and warfarin), and veterans’ health administration (VHA) healthcare utilization variables |
| Nielsen, 2018 (21),  - Denmark | - patients with AD incident from January 1, 2000, to December 31, 2011;  total n = 32,001  2000-2002  Total n = 4,794 (65% female), exposed n = 2,226 (62.7% female)  2003-2005  Total n = 7,839 (67% female), exposed n = 3,001 (64.6% female)  2006-2008  Total n = 8,890 (65% female), exposed n = 2,226 (61.7% female)  2009-2011  Total n = 10,478 (64% female), exposed n = 1,787 (61.4% female) | - mean age at diagnosis of AD: 80.25±7.99 years (men: 78.94 ±8,30 years; women: 80.95 ±7.73 years) | - APDs were defined as N05A, excluding lithium (ATC N05AN) | AD diagnosis received as ICD-10 code F00xx or G30x in nationwide registers from 01.01.2000 until 12.31.2011 | - register based retrospective cohort study  - 2000 to the end of 2011 | All-cause mortality risk  Current and cumulative APD use vs. non-use. aHRs for current use  2000-2002  **aHR = 2.24 (95% CI 2.07, 2.43,**  2003-2005  **aHR = 2.02 (95% CI 1.88, 2.17),**  2006 – 2008  **aHR = 1.71 (95% CI 1.57, 1.85),**  2009 – 2011  **aHR = 1.24 (95% CI 1.09, 1.41),**  - Increasing cumulative APD use in DDD was associated with decreasing mortality hazard rate, both with and without current use, not all results were statistically significant | - Cox proportional hazards model  - all variables as time-dependent  - severity of NPS (via number of psychiatric bed days, number of psychiatric outpatient contacts), psychiatric comorbidity, somatic comorbidity, and cardiovascular risk factors for each defined time period |
| Simoni-Wastila, 2016 (22),  - the US | - total sample size (n = 8,811)  - severe mental illness SMI (n = 5,621),  - dementia with behavioral symptoms (n = 1,090),  - delirium only (n = 2,100) | - SMI  73.4 % female  mean age = 80.1  - dementia with behavioral symptoms  7.8 % female  mean age = 81.3  - delirium only  74.5 % female  mean age= 83.2 | conventional agents  chlorpromazine, droperidol, fluphenazine, haloperidol, loxapine, molindone, perphenazine, pimozide, prochlorperazine, thioridazine, thiothixene, trifluoperazine and  atypical agents  aripiprazole, clozapine, olanzapine, quetiapine, risperidone, ziprasidone, paliperidone, asenapine, iloperidone, lurasidone | ICD-9-CM | - retrospective cohort study  - 2007 to 2009 | All-cause mortality risk of new ADP users  modified standardized daily dose (mSDD) >1 vs mSDD <= 1  **aHR =** **0.52 (95% CI 0.36, 0.76)**  Duration of therapy (reference group 1-30 days)  31 – 60 days  aHR = 1,05 (95% CI 0.77, 1.43)  61 – 90 days  aHR = 0.90 (95% CI 0.64, 1.27)  91 – 184 days  aHR = 0.80 (95% CI 0.61, 1.05) | - discrete-time survival model with complementary log–log regressions  - age, sex, race, region, low income subsidy, number of comorbid condition, MDS cognition scale score, activity of daily living dependency score, MDS changes in health, end-stage disease, signs, and symptoms scale score, any hospitalization, any skilled nursing home stay, medication use at baseline (any antidepressant use, and any anxiolytic or sedative-hypnotic use), length of nursing home months before AP index date |
| Kheirbek, 2019 (23),  - the US | - community-dwelling veterans ages ≥ 65 years between January 2007 and January 2015  - total sample size (n = 730,226)  - dementia diagnosis (n=13385) | - 98% male  - mean age not stated | atypical APD medications  aripiprazole, clozapine, olanzapine, quetiapine, risperidone, and ziprasidone  typical APD medications  chlorpromazine, fluphenazine, haloperi- dol, perphenazine, thioridazine, and trifluoperazine | ICD-9 codes (290.0-  290.4,294.20,331.1,331.8), from Veterans Affairs (VA) and Informatics and Computing Infrastructure Corporate Data Warehourse (VINCI) | - retrospective cohort study  - 2007 to 2015 | Cumulative risk of death within 10 years  Controls (no APD, no dementia) = **42%**  APD-users without dementia = **48%**  Dementia without APD-use = **73%**  Dementia with APD use = **78%**  Mortality risk  Atypical APD use vs. non-use (dementia and non-dementia population)  **aHR = 1.46 (95% CI 1.41, 1.51),**  Typical APD use vs. non-use (dementia and non-dementia population)  **aHR = 2.12 (95% CI 2, 2.24).** | - Cox proportional hazards model with time-varying variables  - time-dependent variable for comorbidities by calculating the Elexhauser comorbidity index at each age interval  - dementia, conventional APDs, atypical APDs, age at time of APDs exposure |
| Arai, 2016 (24),  - Japan | - aged ≥ 65 years with AD at one of 357 medical sites  - total sample size 10 weeks (n = 9,771); 24-weeks (n = 9199)  - APD use 10 weeks (n = 4,873)  - unexposed 10 weeks (n = 4,898) | Exposed:  - 69.4 % female  - mean age 81.6 (6.8) years  Unexposed:  - 72.0 % female  - mean age 81.9 (6.6) | atypical APDs (71.4% of exposed)  quetiapine, risperidone, olanzapine, aripiprazole and others  typical APDs (21.6% of exposed)  tiapride, sulpiride, levomepromazine, haloperidol, chlorpromazine and others | DSM-IV criteria | - 24-week prospective cohort study  - October 2012 to August 2013 | Odds of death  ADP use vs. non-use  0 to 10-weeks  aOR = 1.32 (95% CI 0.81, 2.14),  11 to 24 weeks  aOR = 1.01 (95% CI 0.70, 1.46),  24-weeks  aOR = 1.11 (95% CI 0.82, 1.50),  Only new users (n = 85)  11 to 24-weeks  **aOR = 3.92 (95% CI 1.59, 9.66),**  0 to 24 weeks  **aOR = 2.53 (1.04, 6.14),**  with 1-30d previous APD use  0 to 10-weeks  aOR = 0.73 (95% CI 0.17, 3.13),  11 to 24 weeks  aOR = 1.17 (95% CI 0.49, 2.82),  24-weeks  aOR = 1.04 (95% CI 0.48, 2.22),  with 31-90d previous APD use  0 to 10-weeks  aOR = 1.63 (95% CI 0.72, 3.69),  11 to 24 weeks  aOR = 1.29 (95% CI 0.67, 2.48),  24-weeks  aOR = 1.43 (95% CI 0.85, 2.41),  with 91-180d previous APD use  0 to 10-weeks  aOR = 0.98 (95% CI 0.37, 2.58),  11 to 24 weeks  aOR = 0.98 (95% CI 0.5, 1.9),  24-weeks  aOR = 0.97 (95% CI 0.55, 1.69),  with >180d previous APD use  0 to 10-weeks  aOR = 1.38 (95% CI 0.8, 2.36),  11 to 24 weeks  aOR = 0.84 (95% CI 0.54, 1.31),  24-weeks  aOR = 1.01 (95% CI 0.72, 1.43), | - logistic regression  - age, sex, body weight, height, in/outpatient, comorbidity, impaired level of dementia, care level |
| Yin, 2015 (25),  - China | - newly diagnosed mild-moderate AD patients between January 2007 and June 2010  - total sample size (n = 156), PwAD Risperidone group (n = 22), PwAD control group (n = 29) | AD with sleep disturbance  - 62.37 % female  - mean age = 76.68 years | - all received donezepil  - 3 treatment groups with each: risperidone, zolpidem tartrate, melatonin  - comparison group no drug treatment | Clinical diagnosis (AD) per DSM-IV criteria | - non-randomized intervention study  - | 5-year mortality rate  no significant differences between the 4 groups medicated with atypical APDs, non-benzodiazepine hypnotics, melatonin, or no-drug treatment (no data shown) | - mean comparison, two- tailed test (not further specified)  - no confounders controlled |
| **Hospitalization** | | | | | | | |
| Zakarias, 2021 (26),  - Denmark | - dementia (≥ 65 years)  - total sample size (n = 28,879) | - 60% female  - mean age at dementia diagnosis = 81.6 years | - APDs (N05A, except lithium), benzodiazepines (N05B, N05C), and/or antidepressants (N06A) | Registry diagnosis | - register-based retrospective cohort study  - 2000 to 2015 | 180-day hazard of hospitalization  APD use plus benzodiazepines vs. APD monotherapy  180d: **aHR =** **1.55 (95% CI 1.29, 1.86)** | - Cox proportional hazards model  - age, sex, calendar year, time since dementia diagnosis at index date, Charlson Comorbidity Index score, prior psychiatric disease, total number of drugs used (other than psychotropic drugs), and total number of days on APD treatment. |
| Mueller, 2020 (4),  - the UK | - persons with dementia (excluding Lewy body dementia)  - total sample size (n = 10,106)  - any APD (n = 1,115)  - any typical APD (n = 386)  - any atypical APD (n = 889),  - non-use (n = 8,991)  any typical APD (n = 223) vs. any atypical APD (n = 762) | - 63.2 % female  - mean age at dementia diagnosis= 81.1 years | - any drug listed in chapter 4.2 of the British National Formulary (including 4.2.1 antipsychotic drugs, 4.2.2 antipsychotic depot injections, 4.2.3 drugs used for mania and hypomania (arsenapine, lithium carbonate, lithium citrate, valproic acid) | SLaM Database | - register-based cohort study  - 1 January 2007 to 31 December 2015 | Risk of any emergency hospitalization  any APD vs. non-use  aHR = 0.99 (95% CI 0.92, 1.08)  any Typical APD vs. non-use  aHR = 0.94 (95% CI 0.82, 1.06)  any Atypical APD vs. non-use  aHR = 1.02 (95% CI 0.93, 1.11)  any Typical APD vs. any Atypical APD use  aHR = 0.89 (95% CI 0.74, 1.07)  Risperidone (n = 283) vs. any other APD use (n=832)  aHR = 1.00 (95% CI 0.84, 1.18)  Risperidone (n = 283) vs. no APD-use  aHR = 0.99 (95% CI 0.85, 1.15)  Olanzapine (n = 162) vs. any other APD use (n=953)  aHR = 0.81 (95% CI 0.65, 1.00)  Olanzapine (n = 162) vs. No APD use  aHR = 0.83 (95% CI 0.67, 1.01)  Quetiapine (n = 402) vs. any other APD use (n=713)  aHR = 1.12 (95% CI 0.97, 1.30)  Quetiapine (n = 402) vs no APD use  aHR = 1.07 (95% CI 0.95, 1.20) | - Cox proportional hazards model  - adjusted for age, gender, marital status, ethnicity, index of deprivation, MMSE score, dementia subtype, HoNOS scores (agitation, psychosis, non-accidental self-injury, problem-drinking or drug taking, depressed mood, physical illness or disability, activities of daily living, living conditions, occupational/recreational activities, social relationships), and hospitalisation in the year prior to dementia diagnosis |
| Mueller, 2020 (4),  - the UK | - persons with dementia (excluding Lewy body dementia)  - total sample size (n = 10,106)  - any APD (n = 1,115)  - any typical APD (n = 386)  - any atypical APD (n = 889),  - non-use (n = 8,991)  any typical APD (n = 223) vs. any atypical APD (n = 762) | - 63.2 % female  - mean age at dementia diagnosis= 81.1 years | - any drug listed in chapter 4.2 of the British National Formulary (including 4.2.1 antipsychotic drugs, 4.2.2 antipsychotic depot injections, 4.2.3 drugs used for mania and hypomania (arsenapine, lithium carbonate, lithium citrate, valproic acid) | SLaM Database | - register-based cohort study  - 1 January 2007 to 31 December 2015 | Risk of hospitalization due to stroke  Any APD vs. non-use  aHR = 1.09 (95% CI 0.84, 1.40)  any Typical APD vs. non-use  aHR = 1.06 (95% CI 0.71, 1.58)  any Atypical APD vs. non-use  aHR = 1.13 (95% CI 0.86, 1.49)  any Typical APD vs. any Atypical APD use  aHR = 0.86 (95% CI 0.48, 1.57)  Risperidone (n = 283) vs. any other APD use (n=832)  aHR = 0.82 (95% CI 0.46, 1.44)  Risperidone (n = 283) vs. no APD-use  aHR = 0.93 (95% CI 0.55, 1.57)  Olanzapine (n = 162) vs. any other APD use (n=953)  aHR = 0.77 (95% CI 0.36, 1.61)  Olanzapine (n = 162) vs. No APD use  aHR = 0.86 (95% CI 0.42, 1.75)  Quetiapine (n = 402) vs. any other APD use (n=713)  aHR = 1.38 (95% CI 0.88, 2.16)  Quetiapine (n = 402) vs no APD use  aHR = 1.30 (95% CI 0.92, 1.84) | - Cox proportional hazards model  - adjusted for age, gender, marital status, ethnicity, index of deprivation, MMSE score, dementia subtype, HoNOS scores (agitation, psychosis, non-accidental self-injury, problem-drinking or drug taking, depressed mood, physical illness or disability, activities of daily living, living conditions, occupational/recreational activities, social relationships), and hospitalisation in the year prior to dementia diagnosis |
| Koponen, 2019 (27),  - Finland | - community-dwellers with a clinically verified AD diagnosis during 2005-2011  - included APD users (n = 19,909)  - included matched non-users (n=19,909) | - 66.6 % female in both groups (APD initiator and non-initiator)  **-** age group (APD initiators, non-initiators):  <65 years (1.9 %, 1.9% )  65-74 years (13.8%, 13.4%)  75-84 years (52.2%, 53.1%)  ≥85 years (32.1%, 31.7%) | - ATC-class N05A, excluding lithium (N05AN01) and prochlorperazine (N05AB04) | Clinical diagnosis | - register-based MEDALZ (Medication and Alzheimer’s disease) cohort study (n=70718),  - 2-year follow-up | Event rates of all-cause hospital days during 2 year follow up  New APD-users vs. non-users  All hospital days  **aIRR = 1.53 (95% CI 1.47, 1.59),**  Days in general healthcare  **aIRR = 1.56 (95% CI 1.49, 1.63),**  Days in specialized healthcare  **aIRR = 1.37 (95% CI 1.29, 1.46).** | - matched cohort 1:1 via incidence density sampling using time since AD diagnosis (±90 days), age (±2 years), sex  - negative binomial model to calculate incident rate ratios  - age, sex, time since diagnosis of AD, log(person-time), use of benzodiazepines, antidepressants, opioids, number of otherthan psychotropic drugs at start of follow-up; prior stroke, hip fracture, cardiovascular disease, asthma/COPD, diabetes, number of in-hospital days within one year prior to the start of follow-up |
| **Long term care and nursing home admission** | | | | | | | |
| Nerius, 2018 (13),  - Germany | - persons with dementia ≥ 60 years newly-diagnosed between January 2006 and December 2010  - total sample size (n = 27,801)  - final study cohort outcome LTC dependency (n = 6,930),  - total sample size outcome NH (n = 9,950). | - LTC dependency  - 4 006 person-years (men)  - 6 605 person-years (women)  - mean age = 78.8±7.4 years  - NH admission  - 6 628 person-years (men)  - 12 501 person-years (women)  - mean age = 80.1 years | - haloperidol, melperone, risperidone, quetiapine, other typical apd (=FGA), other atypical apd (=SGA) | ICD-10 codes G30, G31.0, G31.82, G23.1, F00, F01, F02, F03, and F05.1 in administrative claims data of AOK (Germany) | - cohort study  - January 2006 and December 2010 | Hazard of LTC dependency  Haloperidol vs. non-Haloperidol-use  **aHR = 2.12 (95% 1.78, 2.52)**  Risperidone vs. non- Risperidone-use  **aHR = 2.08 (95% CI 1.87, 2.32)**  Quetiapine vs. non- Quetiapine -use  **aHR = 1.64 (95% CI 1.35, 1.98)**  Melperone vs. non- Melperone -use  **aHR = 2.34 (95% CI 2.13, 2.58)**  Other typical APDs vs non-use  **aHR = 2.13 (95% CI 1.87, 2.42)**  Other atypical APDs vs non-use  **aHR = 0.78 (95% CI 0.54, 1.13)**  Hazard of NH admission  Haloperidol vs. non-Haloperidol-use  **aHR = 1.51 (95% 1.28, 1.80)**  Risperidone vs. non- Risperidone-use  **aHR = 1.56 (95% CI 1.38, 1.75)**  Quetiapine vs. non- Quetiapine -use  **aHR = 1.39 (95% CI 1.15, 1.69)**  Melperone vs. non- Melperone -use  **aHR = 1.74 (95% CI 1.58, 1.93)**  Other typical APDs vs non-use  **aHR = 1.49 (95% CI 1.31, 1.71)**  Other atypical APDs vs non-use  **aHR = 1.40 (95% CI 0.97, 2.02)** | - Cox proportional hazards model  - outcome LTC dependency adjusted for sex, age, comorbidities, polypharmacy, antidementia drug use  - outcome NH admission adjusted for sex, age, comorbidities, polypharmacy, antidementia drug use, LTC dependency, residency |
| Lopez, 2014 (28),  - the US | - probable Alzheimer`s Disease  - total sample size (n = 957) | conventional users  - 66 % female  - mean age= 71.2 years  atypical APD users  - 68% female  -mean age = 73.1 years | - typical or atypical APDs | neuropsychiatric evaluation | - longitudinal observational study  - followed between 1983 and 2005 | Hazard of nursing home admission  Typical APD use  aHR = 1.30 (95% CI 0.95, 1.79),  Atypical APD use  aHR = 1.02 (95% CI 0.61, 1.71) | - Cox proportional hazards model  - age, gender, education level, dementia severity, hypertension, diabetes mellitus, heart disease, extrapyramidal signs, depression, psychosis, aggression, agitation, and dementia medication use |
| Yin, 2015 (25),  - China | - newly diagnosed mild-moderate AD patients between January 2007 and June 2010  - total sample size (n = 156), PwAD Risperidone group (n = 22), PwAD control group (n = 29) | AD with sleep disturbance  - 62.37 % female  - mean age = 76.68 years | - all received donezepil  - 3 treatment groups with each: risperidone, zolpidem tartrate, melatonin  - comparison group no drug treatment | Clinical diagnosis (AD) per DSM-IV criteria | - non-randomized intervention study  - | Percentage of institutionalisation  Significantly lower in PwAD taking Risperidone compared to PwAD in the comparison group (p <0.05) (no data shown) | - mean comparison, two- tailed test (not further specified)  - no confounders controlled |
| **Hip fracture** | | | | | | | |
| Dennis, 2017 (3),  - Wales | - dementia diagnosis at age >= 65y between 2003 and 2011  - total sample size (n = 9,674)  - sample size of outcome hip fracture (n = 9629), APD use (n = 3721), non-use (n = 5908) | Exposed: 66.2% female; mean age at diagnosis of dementia = 82.4 (6.8) years Non-exposed: 67.8 % female; mean age at diagnosis of dementia: 82.5 (6.8) years | - typical and atypical APDs and other psychotrophic medication | SAIL databank, the date of first prescription after dementia diagnosis was recorded from GP computer records using NHS Read codes | - register-based retrospective cohort study  - 2003 to 2011 | Likelihood of hip fracture within 12 months  All dementia types (n = 9,629)  **PERR = 1.62 (CI 95% 1.59, 1.65)**  Only Alzheimer’s dementia (n = 6960)  **PERR = 1.65 (CI 95% 1.61, 1.71)**  Only known to primary care (n = 5061)  **PERR = 1.57 (CI 95% 1.52, 1.65)** | - prior event rate ratio (PERR)  - assumption of constant confounder over 12 months |
| Koponen, 2017 (29),  - Finland | - community-dwellers with a clinically verified AD diagnosis during 2005-2011  - total sample size (n = 57,588),  - new APD users (n = 16,972)  - APD use (n = 16,587)  - no use during follow-up  = 143,671 person-years) | APD users (n= 15 942)  - 64.2 % female  <75 years 24.4 %  75-84 years 56.4 %  ≥ 85 years 19.3 %  non-users during follow-up (n= 41 646)  - 63.7 % female  <75 years 20.3 %  75-84 years 56.1 %  ≥ 85 years 23.5 % | - ATC-class N05A, excluding lithium (N05AN01) and prochlorperazine (N05AB04) | the NINCDS- ADRDA and DSM-IV criteria for AD | - register-based MEDALZ (Medication and Alzheimer’s disease) cohort study  - 2005 to 2011 | Hazard of hip fracture  APD use vs. non use  **aHR = 1.54 (95% CI 1.39, 1.70),**  Duration of APD use (ref group: no use)  1-30 d  **aHR = 1.76 (95% CI 1.28, 2.41)**  31-180 d  **aHR = 1.52 (95 % CI 1.26, 1.82)**  181-365 d  **aHR = 1.40 (95 % CI 1.14, 1.73)**  > 365 d  **aHR =** **1.60 (95 % CI 1.39, 1.85)** | - Cox proportional hazards model  - sex, age, history of stroke, osteroporosis, rheumatoid arthiritis, glaucoma, diabetes, cadiovascular disease, epilepsy, use of other psychotics and opoids at the time of antopsychotic initiation. |
| Jalbert, 2010 (30),  - US | - long- stay Medicaid-eligible NH residents, ≥ 65 years, with a diagnosis of dementia in 2001-2002  - nursing homes in California, Florida, Illinois, New York, Ohio with minimum 20 beds  - Total sample size (n = 4,346) | cases (n = 764)  - 78.4 % female  65-74 years 11.4%  75- 84 years 39.4 %  ≥85 years 49.2 %  controls (n = 3,582)  - 72.9 % female  65-74 years 16.1 %  75- 84 years 42.2 %  ≥85 years 41.8 % | - APD use 30-180 days use before index date (hip fracture) with a maximum 14 days gap before index date; new user = first time user; prevalent user = more than one period ADP use  Atypical APDs  aripiprazole, clozapine, olanzapine, quetiapine, risperidone, and ziprasidone  Typical APDs  chlorpromazine, chlorprothixene, fluphenazine, haloperidol, loxapine, mesoridazine, molindone, perphena- zine, pimozide, prochlorperazine, promazine, thimidazine, thioridazine, thiothixene, and trifluoperazine  or any APD (typical and atypicals) | Clinical diagnosis | - nested case-control study  - 2001 to 2002 | Odds of hip fracture  Any APD  Current (n = 1,463) vs. non-use (n = 2,350)  **aOR = 1.26 (95% CI 1.05, 1.52)**  New (n = 248) vs. non-use (n = 2,350)  **aOR = 1.33 (95% CI 0.95, 1.88)**  Prevalent (n = 1,216) vs. non-use (n=2,350)  **aOR = 1.21 (95% CI 0.99, 1.47)**  Atypical APD  Current (n = 1,369) vs. non-use n = 2,350)  **aOR = 1.27 (95% CI 1.05, 1.54)**  New user (n = 232) vs. non-use (n = 2,350)  **aOR = 1.36 (95% CI 0.95, 1.94)**  Prevalent user (n = 1,137) vs. non-use (n = 2,350)  **aOR = 1.33 (95% CI 1.08, 1.63)**  Typical APD  Current (n = 94) vs. non-use (n = 2,350)  aOR = 1.44 (95% CI 0.84, 2.47)  New user vs. non-user: not reported  Prevalent user (n = 79) vs. non-use (n = 2,350)  aOR = 1.28 (95% CI 0.7, 2.34) | - 5 controls matched for each case via incidence-density sampling (residing in the same facility during the same quarter)  - conditional logistic regression  - atypical APD users adjusted for: age, body mass index, wandering  - typical APD users adjusted for age  - any APD users adjusted for: age, body mass index, wandering, benzodiazepine use |
| Zakarias, 2021 (26),  - Denmark | - dementia (≥ 65 years) initiating APD monotherapy or in combination with benzodiazepines and/or antidespressants between 2000 and 2015  - total sample size (n = 28,879) | - 60% female  - mean age at dementia diagnosis= 81.6 (6.6) years | - APDs (N05A, except lithium), benzodiazepines (N05B, N05C), and/or antidepressants (N06A) | Register-based clinical diagnosis | - register-based retrospective cohort study  - 2000 to 2015 | 180-day hazard of hip fracture  combination of APDs and benzodiazepines vs. APD use  aHR = 1.50 (95% CI 0.99, 2.26) | - Cox proportional hazards model  - age, sex, calendar year, time since dementia diagnosis at index date, Charlson Comorbidity Index score, prior psychiatric disease, total number of drugs used (other than psychotropic drugs), and total number of days on APD treatment. |
| **Stroke and cerebrovascular accidents and events** | | | | | | | |
| Mok, 2024 (31),  - England | - adults ≥ 50 yrs with dementia diagnosis  - sample size (n = 173,910), exposed to APD-use (n = 35,339) | - 63% female,  - mean age = 82.1 ± 7.9 | - Typical and atypical antipsychotics | Linked primary care, hospital, mortality data | - population matched cohort  - between 1 January 1998 and 31 May 2018 | Hazard of stroke  366-2y **aHR =** **1.55 (95% CI 1.38, 1.74),**  181-365d **aHR =** **1.52 (95% CI 1.34, 1.73),**  31-180d **aHR =** **1.54 (95% CI 1.39, 1.70);**  0–7d **aHR = 3.75 (95% CI 3.00, 4.69)**,  8–30d: **aHR = 1.57 (95% CI 1.28, 1.92)** | - propensity score matching based on demographics, comorbidities, smoking, alcohol, polypharmacy including cardiovascular and psychiatric meds  - Cox regression survival analysis  - adjusted for APD use as a time-varying variable, cohort origin, IPTW |
| Koponen, 2022 (32),  Finland | - community-dwellers with a clinically verified AD diagnosis during 2005-2011  - total sample size (n = 70,718)  - initiated APD-use during follow-up (n = 20,467)  - matched controls (n = 20,467) | APD-users  - 68,2 % female  - <65y n=370, 65-74y n=2753, 75-84y n=10492, >=85y n=6852  Non-users (matched)  - 68.2% female  - <65y n=360, 65-74y n=2744, 75-84y n=10550, >=85y 6813 | - Antipsychotics (N05A excl. lithium and prochlorperazine) | - register-based MEDALZ (Medication and Alzheimer’s disease) cohort study (n=70718)  - diagnosis based on NINCDS-ADRDA and DSM-IV | - Register-based matched cohort study  - 2005 to 2011 | Hazard of stroke  APD users vs. non-users  **aHR = 1.09 (95% CI 0.98,1.22);**  1-60 days  **aHR =** **1.73 (95% CI 1.32, 2.28)**;  61-120 days  aHR = 1.30 (95% CI 0.97, 1.74)  121-180 days  aHR = 1.17 (95% CI 0.85, 1.60)  181-365 days  aHR = 0.87 (95% CI 0.7, 1.08)  <365 days  aHR = 0.93 (95% CI 0.77, 1.13)  Quetiapine vs risperidone  **aHR = 1.12 (95% CI 0.91,1.37)** | - matched cohort 1:1 via incidence density sampling using time since AD diagnosis (±90 days), age (±2 years), sex  - inverse probability of treatment-weighted (IPTW) Cox proportional hazards models (stratified by the matched pairs)  - Weights derived using age, sex, time since Alzheimer’s disease diagnosis, use of benzodiazepines and related drugs, antidepressants, antiepileptics, acetylcholinesterase inhibitors, memantine, antithrombotic agents, and nonsteroidal anti-inflammatory drugs, and history of hypertension, ischemic heart disease, chronic heart failure, atrial fibrillation, peripheral vascular disease, diabetes, and head traumas, recent hospitalization due to cardiovascular diseases, and number of hospital days during 6 months preceding the index date |
| Laredo, 2011 (33), | - individuals with dementia aged 65 and older registered in the database between January 1, 1995, and June 22, 2007.  - total sample size (n = 18,762)  - 11,120 APD users  - 7,126 typical APD users  - 1,878 atypical APD users  - 2,116 both typical and atypical APD use | No CVA at end of follow-up  69.7 % female  - mean age= 81.11 years  CVA Cases  68.9% female  -mean age= 80.98 years | - APD defined by ATC code N05A  - Short exposure: <90 days; long exposure: >90 days  - current user: <90 days between APD use and event; noncurrent use: > 90 days between APD use and event | Diagnosis of dementia (ICD-9 codes 290.0-290.4, 331.0, 331.1, 331.82) in the General Practice Research Database (GPRD) | - a population-based case control study  - 1995 to 2007 | Odds of CVA  APD use vs. no APD use  aOR = 0.96 (95% CI 0.89, 1.04)  only Typical APD users vs. no APD use  **aOR =** **1.16 (95% CI 1.07, 1.27),**  only atypical APDs vs. no APD use  **aOR =** **0.62 (95% CI 0.53, 0.72)**  Typical APD use vs. atypical APD use  **aOR =** **1.83 (95% CI 1.57, 2.14).** | - logistic regression models  - sex, age, myocardial infarction, mitral stenosis, atrial fibrillation, heart failure, diabetes mellitus, hyperlipidemia, hypertension, obesity, anticoagulants, platelet inhibitors, lipid-lowering drugs, oral hypoglycemic agents, insulin, and antihypertensive drugs |
| Dennis, 2017 (3),  - Wales | - dementia diagnosis at age ≥65y between 2003 and 2011  - total sample size (n = 9,674)  - sample size of outcome stroke (n = 9,439), APD use (n = 3,652), non-use (n = 5,787) | Exposed: 66.2% female; mean age at diagnosis of dementia = 82.4 (6.8) years Non-exposed: 67.8 % female; mean age at diagnosis of dementia: 82.5 (6.8) years | - typical and atypical APDs and other psychotropic medication | SAIL databank, the date of first prescription after dementia diagnosis was recorded from GP computer records using NHS Read codes | - register-based retrospective cohort study  - 2003 to 2011 | Likelihood of stroke within 12 months  All dementia types (n = 9439)  **PERR = 1.41 (95% CI 1.40, 1.46)**  Only Alzheimer’s dementia (n = 6,860)  **PERR = 2.06 (95% CI 1.97, 2.13)**  Only known to primary care (n = 4,296)  **PERR = 1.73 (95% CI 1.66, 1.75)**  Atypical APD vs. typical APD users (exclusion of double users)  **PERR = 0.82 (95% CI 0.8, 0.9)** | - prior event rate ratio (PERR)  - assumption of constant confounder over 12 months |
| Chan, 2010 (34),  - Hong Kong | - patients aged 65 or above, diagnosed with AD, vascular or mixed dementia, and first attended the psychiatric service of Department of Psychiatry of the Pamela Youde Nethersole Eastern Hospital (PYNEH) between 1st January 2000 to 30th June 2007  - total sample size (n = 1,089)  - typical APD users (n = 654)  - atypical APD users (n = 72)  - non-users (n = 363) | typical APD users  - 66.2 % female  - mean age= 81.48 years  atypical APD users  - 69.4 % female,  - mean age= 79.93years  non-user  - 63.9 % female  - mean age = 80.47 years | - APDs, antidepressants and anxiolytics | Clinical diagnosis | -a retrospective cohort study  - 1st January 2000 to 30th June 2007 | Hazard of cerebrovascular adverse events (CVAEs)  Typical APDs vs. non-use  aHR = 0.964 (95% CI 0.584, 1.591)  Atypical APDs vs. non-use  aHR = 1.036 (95% CI 0.350, 3.066) | - survival analysis and Cox proportional hazards model  - age, sex, marital status, education level, residency, diagnosis, prior stroke, atrial fibrillation, hypertension, diabetes mellitus, ischaemic heart disease, congestive heart disease, rheumatic heart disease, hypercholesterolaemia, smoking, malignant neoplasm, and number of drugs prescribed |
| DeMercy and Brenner, 2024, (35)  - USA | - individuals ≥50 yrs with dementia & BPSD  - total sample size (n =1,126) | - 52.90 % female  - mean age = 78.19 (6.71) years | - Olanzapine, Haloperidol, Quetiapine, Aripiprazole, Risperidone  - low, medium, and high dose defined from relevant publications  - four treatment groups: cognitive enhancers only, APD-use only, cognitive enhancers and APD-use, no treatment | Loma Linda Univ. Med Center database | - Cohort study  - 2008 to 2023 | Risk of Major adverse cardiac/cerebrovascular events (MACCE)  APD use (n = 478) vs. non-use (n = 164)  **aHR = 1.787 (95% CI 1.527, 2.092)**  High dose (n = 13) vs. low dose (n = 187)  **aHR = 3.380 (95% CI 1.880, 6.077)**  Medium dose (n = 39) vs low dose (n = 187)  **aHR = 1.480 (95% CI 1.035, 2.116)** | - Cox proportional hazards model  - age, sex, race, smoking, previous MACCE; no data on comorbidities or dementia severity |
| Liu, 2013  - Taiwan (36) | - patients with dementia aged ≥ 65 years  - total sample (PwD n = 2,243, controls n = 6,714, PwD with antipsychotics use n = 1,450, PwD with non-use n = 793) | - 53.3% female  - mean age = 77.85 | - any APD | ICD-9-Code 290.0 (Senile dementia, uncomplicated), 290.1x (Presenile dementia), 290.2x (Senile dementia with delusional or depressive features), 290.3 (Senile dementia with delirium), 290.4x (Arteriosclerotic dementia), 294.1 (Dementia in conditions classified elsewhere), 331.0 (Alzheimer disease), 331.1 (Pick disease), and 331.2 (Senile degeneration of brain)(36) | - a nationwide 5-year population-based study | 5-year risk of stroke  aHR = **1.17 (95% CI 1.01, 1.40)** | - Cox proportional hazards regression  - age, sex, monthly income, geographic region, hypertension, diabetes. |
| **Falls, orthostatic Hypotension** | | | | | | | |
| Dyer, 2024, (37)  - Europe (9 countries) | - mild-to-moderate AD  - total sample size (n = 509) | - 61.9% female,  - mean age = 72.9 ± 8.3 | - ATC N05A (antipsychotics) | NINCDS-ADRDA criteria; MMSE 12–26 | - clinical trial of blood pressure medication  - 18 months | Sit to stand orthostatic hypotension  **aOR = 1.23 (95% CI 1.05, 1.43)** | - mixed-effects logistic regression, with random effects for study site and individual participant  - Interaction term (antipsychotic use*visits)  - age >75, sex, initial BP category, diabetes mellitus, cholinesterase inhibitors, BPSD, culprit medication use, study group |
| Dyer, 2024, (37)  - Europe (9 countries) | - mild-to-moderate AD  - total sample size (n = 509) | - 61.9% female,  - mean age = 72.9 ± 8.3 | - ATC N05A (antipsychotics) | NINCDS-ADRDA criteria; MMSE 12–26 | - clinical trial of blood pressure medication  - 18 months | Incident falls  IRR = **1.72 (95% CI 1.04, 2.85)** | - mixed-effects poisson regression, with random effects for study site and individual participant  - Interaction term (antipsychotic use*visits)  - age >75, sex, study group, duration since AD diagnosis, baseline cognition, dementia severity, antidepressants, benzodiazepines, Z-drugs, comorbidities, BPSD |
| **Head injuries, fractures** | | | | | | | |
| Tapiainen, 2019 (38),  - Finland | - community dwellers with a clinically verified AD diagnosis during 2005-2011  - total sample size (n = 43,590) | APD users  - 66.3 % female  ≤ 64 years 1.7 %  65-74 years 13.3 %  75-84 years 51.7 %  ≥ 85 years 33.2%  non-users  - 66.3 % female  ≤64 years 1.7 %  65-74 years 13.1 %  75-84 years 52.2 %  ≥ 85 years 33.0 % | - ATC code N05A (APDs), excluding N05AN01 (lithium) and N05AB04 (prochlorperazine) | Register-based clinical diagnosis (NINCDS-ADRDA) and DSM-IV | - register-based MEDALZ (Medication and Alzheimer’s disease) cohort study  - 2005 to 2011 | Hazard of head injuries  APD use vs. non-use  **IPT-weighted HR = 1.29 (95% CI 1.14, 1.47)**  traumatic brain injuries TBIs  APD use vs. non-users  **IPT-weighted HR = 1.22 (95% CI 1.03, 1.45)**  Quetiapine users vs. risperidone users (TBIs)  **IPT-weighted HR =** **1.60 (95% CI 1.15, 2.22)** | - Cox proportional hazards model  - for Inverse probability of treatment IPT weighting and for multivariable adjustments, sociodemographic factors (university hospital catchment area and  occupational socioeconomic position), medicine use (antidepressants, antiepileptic drugs, antithrombotics/anticoagulants, opioids, drugs for Parkinson’s disease, and benzodiazepines or related drugs), and diseases (atrial fibrillation, chronic heart failure, coronary artery disease, hypertensive disease, peripheral arterial disease, stroke, depression or other mood disorder, except mania/bipolar, substance abuse, asthma/chronic obstructive pulmonary disease, cancer, diabetes, epilepsy, hip fracture, and renal failure). Due to a lack of matching in drug-comparison analyses, for IPT-weighted and adjusted drug comparisons, additionally, sex, age group, and time since AD diagnosis are added. |
| Mok, 2024 (31),  - England | - adults ≥ 50 yrs with dementia diagnosis  - sample size (n = 7,714), exposed to APD-use (n = 626) | - 63% female,  - mean age = 82.1 ± 7.9 | - Typical and atypical antipsychotics | Linked primary care, hospital, mortality data | - population matched cohort  - between 1 January 1998 and 31 May 2018 | Hazard of Fractures  180 d **aHR =** **1.43 (95% CI 1.35, 1.52)**  0–7d **aHR =** **2.22 (95% CI 1.66, 2.98)**  8–30d **aHR =** **1.49 (95% CI 1.22, 1.83)**  31–180d **aHR =** **1.37 (95% CI 1.24, 1.52)**,  181–365d **aHR =** **1.29 (95% CI 1.14, 1.46)**,  366d–2y **aHR =** **1.53 (95% CI 1.38, 1.71)** | - Cox proportional hazards model  - propensity score matching based on demographics, comorbidities, smoking, alcohol, polypharmacy including cardiovascular and psychiatric meds |
| **Pneumonia** | | | | | | | |
| Tolppanen, 2016 (39),  - Finland | - Finnish community dwellers who received clinically verified AD diagnosis in 2005 to 2011  - AD cohort Total sample size (n = 60,584)  - users (n = 17,031)  - non-users (n = 43,553)  - matched comparison with out AD (n = 60,584)  - users (n = 2,714)  - non-users (n = 57,870) | AD cohort  user  - 65.2 % female  - mean age= 79.0 (7.1) years  non-user  - 65.1 % female  - mean age = 80.4 (6.9) years  non-AD cohort  user  - 70.8 % female  - mean age= 82.5 (6.3) years  non-user  - 64.9 % female  - mean age= 79.9 (7.0) years | - APD drugs (ATC code N05A, excluding Lithium and prochlorperazine) quetiapine, risperidone, haloperidol) | Clinical diagnosis | - register-based cohort study  - 2005 to 2011 | Hazard of Pneumonia  AD cohort: APD use vs. non-use  aHR = **2.01 (95% CI 1.90, 2.13)**  Non-AD cohort: APD use vs. non-use  aHR = **3.43 (95% CI 2.99, 3.93)** | - Cox regression analysis  - APD use as a time-dependent variable  - propensity score matching for APD-users and non-users via sex, age, calendar year, occupational social class, ongoing cancer treatment at the beginning of follow up, use of cardiovascular drugs, diagnosis and drugs for obstructive airway diseases, ischaemic heart disease, stroke, substance abuse, anaemia, hip fractures, use of analgesucs, antiepileptics and drugs for Parkinson`s disease, mental and behavioral disorders, dementia-related hospital admissions, use of antidepressants, benzodiazepines and related drugs, antineoplastic and immunomodulatory drugs, use of APD before washout period, recent use of proton pump inhibitors, (additionally only AD cohort: congenstive heart failure, recent use of antibiotics), (additionally only non-AD cohort: history of metastatic cancer, arrhytmia, hypertension, renal diseases, fluid and electrolyte disorders) |
| Mok, 2024 (31),  - England | - adults ≥ 50 yrs with dementia diagnosis  - sample size (n = 13,009), exposed to APD-use (n = 1,849) | - 63% female,  - mean age = 82.1 ± 7.9 | - Typical and atypical antipsychotics | Linked primary care, hospital, mortality data | - population matched cohort  - between 1 January 1998 and 31 May 2018 | Hazard of Pneumonia  180 d **aHR =** **2.19 (95% CI 2.10, 2.28),**  0–7d **aHR =** **9.99 (95% CI 8.78, 11.40)**,  8–30d **aHR =** **3.39 (95% CI 3.04, 3.77),**  31–180d **aHR =** **2.03 (95% CI 1.89, 2.17)**,  181–365d **aHR =** **1.79 (95% CI 1.64, 1.95)**,  366d–2y **aHR =** **1.71 (95% CI 1.58, 1.85)** | - Cox proportional hazards model  - propensity score matching based on demographics, comorbidities, smoking, alcohol, polypharmacy including cardiovascular and psychiatric meds |
| **MI and heart failure** | | | | | | | |
| Mok, 2024 (31),  - England | - Adults ≥50 yrs with dementia diagnosis  - sample size (n = 2,626), exposed to APD-use (n = 206) | - 63% female,  - mean age = 82.1 ± 7.9 | - Typical and atypical antipsychotics | Linked primary care, hospital, mortality data | - population matched cohort  - between 1 January 1998 and 31 May 2018 | Hazard of Myocardial infarction  180 d **aHR =** **1.28 (95% CI 1.15, 1.42),**  0–7d **aHR =** **2.33 (95% CI 1.41, 3.83),**  8–30d **aHR =** **1.61 (95% CI 1.15, 2.26),**  31–180d **aHR =** **1.27 (95% CI 1.06, 1.52),**  181–365d **aHR =** **1.39 (95% CI 1.13, 1.70),**  366d–2y **aHR =** **1.02 (95% CI 0.83, 1.27)** | - Cox proportional hazards model  - propensity score matching based on demographics, comorbidities, smoking, alcohol, polypharmacy including cardiovascular and psychiatric meds |
| Mok, 2024 (31),  - England | - adults ≥50 yrs with dementia diagnosis  - sample size (n = 5,751), exposed to APD-use (n = 476) | - 63% female,  - mean age = 82.1 ± 7.9 | - Typical and atypical antipsychotics | Linked primary care, hospital, mortality data | - population matched cohort  - between 1 January 1998 and 31 May 2018 | Hazard of heart failure  180 d **aHR =** **1.27 (95% CI 1.18, 1.37)**,  0–7d **aHR =** **2.85 (95% CI 2.15, 3.78)**,  8–30d **aHR =** **1.95 (95% CI 1.59, 2.40**),  31–180d **aHR =** **1.32 (95% CI 1.17, 1.49)**,  181–365d aHR = 1.12 (95% CI 0.95, 1.31),  366d–2y aHR = 0.97 (95% CI 0.82, 1.14). | - Cox proportional hazards model  - propensity score matching based on demographics, comorbidities, smoking, alcohol, polypharmacy including cardiovascular and psychiatric meds |
| Dennis, 2017 (3),  - Wales | - dementia diagnosis at age ≥ 65y between 2003 and 2011  - total sample size (n = 9,674)  - sample size of outcome acute cardiac event (n = 9,629), APD use (n = 3,721), non-use (n = 5,787) | Exposed: 66.2% female; mean age at diagnosis of dementia = 82.4 (6.8) years Non-exposed: 67.8 % female; mean age at diagnosis of dementia: 82.5 (6.8) years | - typical and atypical APDs and other psychotrophic medication | SAIL databank, the date of first prescription after dementia diagnosis was recorded from GP computer records using NHS Read codes | - register-based retrospective cohort study  - 2003 to 2011 | Likelihood of acute cardiac event within 12 months  All dementia types (n = 9,629)  **PERR = 0.98 (95%CI 1.00, 1.00)**  Only Alzheimer’s dementia (n = 6,960)  **PERR = 1.65 (95% CI 1.59, 1.86)**  Only known to primary care (n= 5,061)  **PERR = 1.68 (95% CI 1.05, 1.78)**  Atypical APD vs. typical APD users (exclusion of double users)  **PERR = 3.3 (95% CI 3.0, 5.1)** | - prior event rate ratio (PERR)  - assumption of constant confounder over 12 months |
| **Venous thromboembolism** | | | | | | | |
| Mok, 2024 (31),  - England | - adults ≥ 50 yrs with dementia diagnosis  - outcome specific sample size (n = 2,168), exposed to APD-use (n = 218) | - 63% female,  - mean age = 82.1 ± 7.9 | - Typical and atypical antipsychotics | Linked primary care, hospital, mortality data | - population matched cohort  - between 1 January 1998 and 31 May 2018 | Hazard of venous thromboembolism  180 d **aHR = 1.62 (95% CI 1.46, 1.80)**,  0–7d **aHR =** **2.05 (95% CI 1.19, 3.56),**  8–30d **aHR =** **1.92 (95% CI 1.36, 2.70),**  31–180d **aHR = 1.67 (95% CI 1.41, 1.99),**  181–365d **aHR = 1.39 (95% CI 1.10, 1.75),**  366d–2y **aHR =** **1.61 (95% CI 1.33, 1.96)** | - Cox proportional hazards model  - propensity score matching based on demographics, comorbidities, smoking, alcohol, polypharmacy including cardiovascular and psychiatric meds |
| Dennis, 2017 (3),  - Wales | - dementia diagnosis at age ≥ 65y between 2003 and 2011  - total sample size (n = 9,674)  - sample size of outcome VTE (n = 9,626), APD use (n = 3,717), non-use (n = 5,909) | Exposed: 66.2% female; mean age at diagnosis of dementia = 82.4 (6.8) years Non-exposed: 67.8 % female; mean age at diagnosis of dementia: 82.5 (6.8) years | - typical and atypical APDs and other psychotrophic medication | SAIL databank, the date of first prescription after dementia diagnosis was recorded from GP computer records using NHS Read codes | - register-based retrospective cohort study  - 2003 to 2011 | Likelihood of venous thromboembolism within 12 months  All dementia types (n = 9,626)  **PERR = 1.95 (95% CI 1.83, 2.0)**  Only Alzheimer’s dementia (n = 6,960)  **PERR = 1.80 (CI 95% CI 1.67, 1.89)**  Only known to primary care (n = 5,055)  **PERR = 2.66 (95% CI 2.41, 2.78)** | - prior event rate ratio (PERR)  - assumption of constant confounder over 12 months |
| **Acute kidney injury** | | | | | | | |
| Mok, 2024 (31),  - England | - adults ≥50 yrs with dementia diagnosis  - outcome specific sample size (n = 6363), exposed to APD-use (n = 657) | - 63% female,  - mean age = 82.1 ± 7.9 | - Typical and atypical antipsychotics | Linked primary care, hospital, mortality data | - population matched cohort  - between 1 January 1998 and 31 May 2018 | Hazard of acute kidney injury (hospital or death record only)  180 d **aHR =** **1.72 (95% CI 1.61, 1.84)**,  0–7d **aHR =** **3.79 (95% CI 2.96, 4.87)**,  8–30d **aHR =** **2.61 (95% CI 2.17, 3.13)**,  31–180d **aHR =** **2.03 (95% CI 1.84, 2.25)**,  181–365d **aHR =** **1.27 (95% CI 1.09, 1.48)**,  366d–2y **aHR =** **1.26 (95% CI 1.10, 1.44).** | - Cox proportional hazards model  - propensity score matching based on demographics, comorbidities, smoking, alcohol, polypharmacy including cardiovascular and psychiatric meds |
| **Cognitive decline** | | | | | | | |
| Vigen, 2011 (40),  - the US | - AD outpatients with psychosis or agitated/ aggressive behavior,  - total sample included (n = 357)  - follow up at 12 weeks (n=342)  - follow up at 24 weeks (n=320)  - follow up at 36 weeks (n=307) | - 54 % female  - mean age= 77.6 (7.4) years | - olanzapine, quetiapine, risperidone (Phase 1), citalopram or an atypical APD (phase 2) | DSM-IV criteria for dementia of the Alzheimer’s type or NINCDS-ADRDA criteria for probable Alzheimer’s disease | - randomized trial (Clinical Antipsychotic Trials of Intervention Effectiveness- Alzheimer’s disease study (CATIE-AD))  - 36 weeks | Cognitive Decline over 36 weeks  Olanzapine, quetiapine or risperidone vs. Placebo  MMSE: -2.67 (-3.62, 1.82) vs. -0.21 (-1.89, 1.46); p-value 0.004  BRPS-Cog: 0.24 (-0.07, 0.54) vs. -0.53 (-1.19, 0.13); p-value 0.05  ADAS-Cog: 5.21 (3.41, 7.01), 2.46 (-0.96, 5.88); p-value 0.11  Cognitive Change per week, mean difference APD vs. placebo  **Olanzapine vs. Placebo**  MMSE -0.080, p-value 0.05  BPRS-Cog 0.014, p-value 0.46  ADAS-Cog 0.073, p-value 0.41  **Quetiapine vs. Placebo**  MMSE -0.045, p-value 0.26  BPRS-Cog 0.036 p-value 0.05  ADAS-Cog 0.073, p-value 0.40  **Risperidone vs. Placebo**  MMSE -0.055, p-value 0.19  BPRS-Cog 0.008, p-value 0,68  ADAS-Cog 0.141, p-value 0.13 | - mixed effects linear regression models to test cognitive change over time; fixed effects age, gender, education and pooled study site; random effects for time on-study in weeks (intercept and slope)  - Generalized Estimating Equations to test correlation of cognitive change (CGIC score) in APD-users and non-users |
| **Quality of Life** | | | | | | | |
| Ven-Vakhteeva, 2012 (41),  - Netherlands | - Persons with dementia living in nursing homes, GDS score < 7  - total sample size (n = 207); exposed n= 65 | patients using APDs  - 64.6 % female  - mean age= 89.3 (7.3) years  patients not using APDs  - 76.1 % female  - mean age = 83.9 (6.5) years | - APDs, anxiolytics, hypnotics, antidepressants (ATC classification) | DSM-IV | - a longitudinal study  - 2 years (every 6 months) | Average change of Quality of life (QUALIDEM)  - no statistical significant change (data not shown) | - Generalized estimating equations (GEE), Model 1 using intervals, Model 2 using time points; Wald's chi-square test  - NPI-NH, SIB-s, and ADL-MDS-SF scores, age, sex, other psychotropic medication prescription patterns  - Model 2 (ADP vs non-use): NPI-NH, SIB-s, and ADL-MDS-SF scores, age, sex |
| Ito, 2020 (42),  - Norway | - aged > 65 years with and without dementia living in nursing homes, included in the COSMOS trial  -total sample size (n = 431)  - APDs use (n = 51) | - 74.9 % female  - mean age = 86.7 years | - N05A APDs | MMSE score | - cross-sectional analysis (secondary analysis of the COSMOS trial)  - 2014 to 2015 | Quality of life via QUALID  MMSE 18-23  QUALID beta = 0.96 (95% CI 0.74, 1.24)  QUALIDEM aOR = 1.06 (95% CI 0.94, 1.21)  MMSE 12-17  QUALID aOR=0.96 (95% CI 0.87, 1.07)  QUALIDEM aOR = 1.01 (95% CI 0.97, 1.07)  MMSE 0-11 | - linear regression  - NPI-NH total score, age, and gender |
| **Adverse events** | | | | | | | |
| Bangash, 2017 (43),  - the UK | - dementia  - total sample size (n = 1,000) | - 59% female  - age  < 65 years 3 %  65- 74 years 17 %  75-84 years 43 %  > 85 years 37 % | - risperidone, haloperidol, quetiapine, aripiprazole, olanzapine, amisulpride | ICD-10 criteria | - A retrospective cohort study  - 2013 to 2015 | Prevalence of any adverse outcome  Any APD-use (n = 205) 24.9%  Risperidone-use (n = 98) 28.6%  Haloperidol-use (n = 10) 10%  Quetiapine-use (n = 61) 21.3%  Aripripazole-use (n = 11) 36.3%  Olanzapine-use (n = 15) 13.3%  Amisulpride-use (n = 10) 0.3% | - prevalence during study period  - no confounders adjustment |
| Sepassi, 2019 (44),  - USA | - patients age 65 years of age or older with AD as a likely comorbidity using *International Classification of Diseases, Common Modification* (ICD- 9-CM) code 331.0 in any of the secondary diagnosis code positions,  - total sample size (n = 20,920,523) | - 57.9 % female  - age  65-74 years 43.7 %  75-84 years 33.8 %  85+ years 22.5 % | - benzodiazepines, APDs, and autonomic nervous system-affecting agents (adrenergic agonists, antimuscarinic agents, anticholinergic agents) | ICD-9 CM code 331.0, identified from the Nationwide Emergency Department Sample (NEDS) | - a retrospective analysis  - 2013 to 2014 | Emergency department visit and adverse events  - there were 427,969 Alzheimer’s emergency department (ED) visits compared with 20,492,554 ED visits without AD  - of the AD cases, 1.04% were associated with at least 1 adverse event  - AD cases more frequently were admitted as inpatients (64.90% vs. 34.92%, P < 0.01)  - common drug classes associated with AD-related ADEs were benzodiazepines, APDs, and autonomic nervous system-affecting agents (adrenergic agonists, antimuscarinic agents, anticholinergic agents)  - there was a significantly higher likelihood for Alzheimer’s cases to experience any APD-related adverse event | - multiple logistic regression  - influential characteristics, including age category, number of Elixhauser comorbidities present, community income level, sex, and geographic hospital region |
| Beeber, 2022, (45)  - USA | - assisted living residents with dementia  - APD users (n = 283) | - 64.3% female  - mean age = 61.4 years | - haloperidole, perphenazine, aripiprazole, olanzapine, quetiapine, risperidone, ziprasidone as dose equivalents of 100mg chloropromazine | Dementia diagnosis via chart data | - cross-sectional descriptive study  - assisted living communities from Arkansas, Luoisiana, New Jersey, New York, Oklahoma, Pennsylvania, Texas | Prevalence of adverse events in previous month  Any APD-use (n = 283):  Any adverse event: 5.7%  Seizure: 2.8%  Heart attack or myocardial infarction: 0.4%  Stroke or transient ischaemic attack: 1.4%  Hip fracture:1.4%  Quetiapine use (n = 153)  Any adverse event: 5.9%  Seizure: 2.6%  Heart attack or myocardial infarction: 0.7%  Stroke or transient ischaemic attack: 1.3%  Hip fracture:1.3%  Risperidone use (n = 64)  Any adverse event: 7.8%  Seizure: 4.7%  Heart attack or myocardial infarction: 0.0%  Stroke or transient ischaemic attack: 1.6%  Hip fracture:1.6% | - Descriptive statistical analysis, Fisher's exact test for comparing potential side effects and adverse event rates |
| *Abbreviations*: aHR= adjusted hazard ratio, AD= Alzheimer's disease, ADL = activities of daily living, ADL-MDS-SF = The activities of daily living-Minimum Database, short form, CI= confidence interval, ESS = Epworth Sleepiness Scale, GDS = geriatric depression scale, HoNOS = the Health of the Nation Outcome Scales, MMSE = mini-mental state examination, NPI-NH= neuropsychiatric inventory nursing home version, OR= odds ratio, PwD= Persons living with dementia, RR= relative risk, SIB-s = Severe impairment Battery-short version ; NINCDS-ADRDA = Register-based clinical diagnosis (the National Institute of Neurological and Communicative Disorders and Stroke and the Alzheimer’s Disease and Related Disorders Association; DSM-IV = Diagnostic and Statistical Manual of Mental Disorders, Fourth Edition  ATC code = anatamoical therapeutic chemical code, N05A = APDs, N05AA = phenothiazines with aliphatic side-chain, N05AB = Phenothiazines with piperazine structure, N05AC = Phenothiazines with piperidine structure, N05AD = Butyrophenone derivatives, N05AE = Indole derivatives, N05AF = Thioxanthene derivatives, N05AH = Diazepines, oxazepines, thiazepines and oxepines, N05AL = benzamides, N05AX = other APDs, N05AB04 = prochlorperazine, N05BB01= hydroxyzine, N05CM06 = propiomazine, R06AD = phenothiazine derivatives,  *Bold means statistically significant* | | | | | | | |

Table 3 Quality assessment using the Newcastle-Ottawa Scale (NOS) for observational and cross-sectional studies

| Author, Year | Selection | Comparability | Outcome | Exposure (For case-control studies) | Total NOS scores |
| --- | --- | --- | --- | --- | --- |
| Nielsen, 2016 (19) | **** | ** | *** |  | 9 good quality study |
| Schwertner, 2019 (1) | **** | ** | *** |  | 9 good quality study |
| Rossom, 2010 (20) | **** | ** | *** |  | 9 good quality study |
| Musicco, 2011 (12) | **** | ** | *** |  | 9 good quality study |
| Jennum, 2015 (11) | **** | ** | *** |  | 9 good quality study |
| Chu, 2018 (9) | **** | ** | *** |  | 9 good quality study |
| Hamedami, 2022 (8) | **** | ** | *** |  | 9 good quality study |
| Liu, 2013 (36) | **** | ** | *** |  | 9 good quality study |
| Nerius, 2017 (13) | *** | ** | *** |  | 8 good quality study |
| Tolppanen, 2016 (39) | **** | ** | ** |  | 8 good quality study |
| Chan, 2010 (34) | *** | ** | *** |  | 8 good quality study |
| Kheirbek, 2019 (23) | *** | ** | *** |  | 8 good quality study |
| Arai, 2016 (24) | **** | ** | ** |  | 8 good quality study |
| Simoni-Wastila, 2016 (22) | *** | ** | *** |  | 8 good quality study |
| Dennis, 2017 (3) | *** | ** | *** |  | 8 good quality study |
| Tapiainen, 2020 (38) | **** | ** | ** |  | 8 good quality study |
| Zakarias, 2021 (26) | *** | ** | *** |  | 8 good quality study |
| Koponen, 2017 (2) | **** | ** | ** |  | 8 good quality study |
| Langballe, 2014 (18) | *** | ** | *** |  | 8 good quality study |
| Koponen, 2017 (29) | **** | ** | ** |  | 8 good quality study |
| Maust, 2015 (16) | *** | ** | *** |  | 8 good quality study |
| Nielsen, 2018 (46) | *** | ** | *** |  | 8 good quality study |
| Koponen, 2022 (32) | **** | ** | ** |  | 8 good quality study |
| Dyer, 2024 (37) | *** | ** | *** |  | 8 good quality study |
| Mok, 2024 (31) | **** | ** | ** |  | 8 good quality study |
| Norgaard, 2022 | **** | ** | ** |  | 8 good quality study |
| Mueller, 2020 (4) | **** | ** | ** |  | 8 good quality study |
| Koponen, 2019 (27) | *** | ** | ** |  | 7 good quality study |
| Harrison, 2021 (10) | *** | ** | ** |  | 7 good quality study |
| Brännström, 2017 (7) | ** | ** | *** |  | 7 good quality study |
| Kales, 2012 (15) | ** | ** | *** |  | 7 good quality study |
| Sepassi, 2019 (44) | *** | ** | ** |  | 7 good quality study |
| Laredo, 2011 (33) | *** | ** |  | ** | 7 good quality study |
| Phiri, 2022 (17) | *** | * | *** |  | 7 good quality study |
| Virginie, 2012 (5) | *** | ** | * |  | 6 fair quality study |
| Sultana, 2014 (14) | *** | * | ** |  | 6 fair quality study |
| Jalbert, 2010 (30) | ** | ** | - | ** | 6 fair quality study |
| DeMercey and Brenner, 2024 (35) | ** | * | *** |  | 6 fair quality study |
| van de Ven-Vakhteeva, 2012 (41) | ** | ** | ** |  | 6 fair quality study |
| Bangash, 2017 (43) | ** |  | *** |  | 5 fair quality study |
| Lopez, 2013 (28) | ** | ** | * |  | 5 fair quality study |
| Ito, 2020 (42) | *** | * | * |  | 5 fair quality study |
| Beeber, 2022 (45) | *** |  | ** |  | 5 fair quality study |
| Yin, 2015 | *** |  | * |  | 4 fair quality study |

Table 4 Quality assessment using the Cochrane Risk of Bias Tool

| Author, year | Random sequence generation (selection bias) | Allocation concealment (selection bias) | Blinding of participants and personnel (performance bias) | Blinding of outcome assessment (detection bias) | Incomplete outcome data (attrition bias) | Selective reporting (reporting bias) | Other bias | Overall |
| --- | --- | --- | --- | --- | --- | --- | --- | --- |
| Vigen, 2011 (40) | 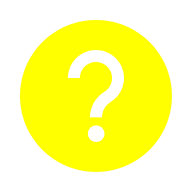 | 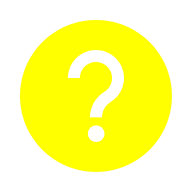 | 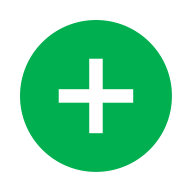 | 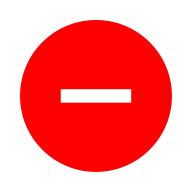 | 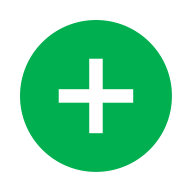 | 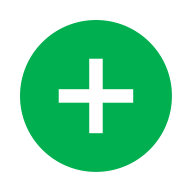 | 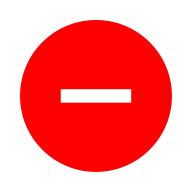 | 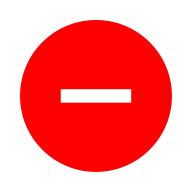 |
| Risk of bias summary: a review of authors’ assessment of each risk of bias item for included studies. + indicates low risk; - high risk; ? unclear risk. | | | | | | | | |

Table 5 GRADE evidence profile of any antipsychotic use in persons with dementia

| **Certainty assessment** | | | | | | | **№ of patients** | | **Effect** | **Certainty** | **Importance** |
| --- | --- | --- | --- | --- | --- | --- | --- | --- | --- | --- | --- |
| **№ of studies** | **Study design** | **Risk of bias** | **Inconsistency** | **Indirectness** | **Imprecision** | **Other considerations** | **any antipsychotics** | **non-use** | **Relative (95% CI)** |  |  |
| Risk of mortality (follow-up: range 2 years to 12 years; assessed with: hazard ratio); **Bibliography:** Chu (2018), Brännström (2017), Dennis (2016), Virginie (2012), Mueller (2021), Harrison (2021), Jennum (2015), Schwertner (2019), Koponen (2017), Langballe (2014), Norgaard (2022), Hamedani (2022), Phiri (2022), Nielsen (2016), Sultana (2014), Musicco (2011) for mortality, Mok(2024), Koponen(2022), Chan (typical)(2010), Chan (atypical)(2010), Liu (2013) for risk of CVE | | | | | | | | | | | |
| 16^a^ | non-randomised studies | not serious | not serious^a^ | not serious | not serious | all plausible residual confounding would reduce the demonstrated effect dose response gradient | 69337 participants | 289933 participants | **HR 1.32** (1.12 to 1.56) [Risk of mortality ] | ⨁⨁⨁⨁ High^a^ | CRITICAL |
| Risk of cerebrovascular adverse events (follow-up: mean 10.6 years; assessed with: Hazard ratio) | | | | | | | | | | | |
| 5 | non-randomised studies | not serious | serious^b^ | not serious | not serious | publication bias strongly suspected all plausible residual confounding would reduce the demonstrated effect dose response gradient^c^ | 24929 participants | 46249 participants | **HR 1.77** (0.92 to 3.42) [Risk of cerebrovascular adverse events] | ⨁⨁◯◯ Low^b,c^ | CRITICAL |
| **CI:** confidence interval; **HR:** hazard ratios  Explanations  a. Heterogeneity I2 value is high (>90%). However, the difference in results can be linked to the different types of study population; the effect is higher in community-dwelling populations than in other clinical or nursing home populations.  b. Heterogeneity is high I2 value >90%.  c. Funnel plot shows asymmetry. | | | | | | | | | | | |

Table 6 GRADE evidence profile of typical antipsychotic use in persons with dementia

| **Certainty assessment** | | | | | | | **№ of patients** | | **Effect** | **Certainty** | **Importance** |
| --- | --- | --- | --- | --- | --- | --- | --- | --- | --- | --- | --- |
| **№ of studies** | **Study design** | **Risk of bias** | **Inconsistency** | **Indirectness** | **Imprecision** | **Other considerations** | **typical antipsychotics use** | **non-use** | **Relative (95% CI)** |  |  |
| Risk of mortality (follow-up: range 2 years to 12 years; assessed with: hazard ratio) ; Bibliography: Chu (2018), Mueller (2021), Harrison (2021), Schwertner (2019), Jennum (2015), Musicco (2011) | | | | | | | | | | | |
| 6 | non-randomised studies | not serious | not serious^a^ | not serious | not serious | all plausible residual confounding would reduce the demonstrated effect dose response gradient | 1956 participants | 75316 participants | **HR 1.25** (1.01 to 1.55) [Risk of mortality] | ⨁⨁⨁⨁ High^a^ | CRITICAL |
|  |  |  |  |  |  |  | - | 0.0% |  |  |  |

Table 7 GRADE evidence profile of atypical antipsychotic use in persons with dementia

| **Certainty assessment** | | | | | | | **№ of patients** | | **Effect** | **Certainty** | **Importance** |
| --- | --- | --- | --- | --- | --- | --- | --- | --- | --- | --- | --- |
| **№ of studies** | **Study design** | **Risk of bias** | **Inconsistency** | **Indirectness** | **Imprecision** | **Other considerations** | **atypical antipsychotics use** | **non-use** | **Relative (95% CI)** |  |  |
| Risk of mortality (follow-up: range 2 years to 12 years; assessed with: hazard ratio) | | | | | | | | | | | |
| 8 | non-randomised studies | not serious | not serious^a^ | not serious | not serious | all plausible residual confounding would reduce the demonstrated effect dose response gradient | 5305 participants | 77867 participants | **HR 1.23** (1.05 to 1.43) [Risk of mortality] | ⨁⨁⨁⨁ High^a^ | CRITICAL |
|  |  |  |  |  |  |  | - | 0.0% |  |  |  |

**Search strategy**

|  | **2021-05-04** |  |
| --- | --- | --- |
| **PubMed** |  |  |
| #1 | (((("Dementia"[Mesh]) OR "Alzheimer Disease"[Mesh]) OR "Huntington Disease"[Mesh]) OR "Frontotemporal Dementia"[Mesh]) OR "Lewy Body Disease"[Mesh] | 173556 |
| #2 | dement*[Title/Abstract] OR huntington*[Title/Abstract] OR alzheimer*[Title/Abstract] OR "frontotemporal diseas*"[Title/Abstract] OR "lewy body*"[Title/Abstract] OR lewy-body*[Title/Abstract] OR lewybody*[Title/Abstract] OR "severe cognitive impairment"[Title/Abstract] | 252016 |
| #3 | #1 OR #2 | 280576 |
| #4 | "drug therap*"[Title/Abstract] OR "drug treatment?"[Title/Abstract] OR "medicament therap*"[Title/Abstract] OR "medicament treatment?"[Title/Abstract] OR medication?[Title/Abstract] OR "medicinal therapy"[Title/Abstract] OR "medicinal treatment"[Title/Abstract] OR "pharmaceutical therap*"[Title/Abstract] OR "pharmaceutical treatment?"[Title/Abstract] OR pharmaco-therap*[Title/Abstract] OR pharmaco-treatment?[Title/Abstract] OR "pharmacological therap*"[Title/Abstract] OR "pharmacological treatment"?[Title/Abstract] OR pharmacotherap*[Title/Abstract] OR pharmacotreatment?[Title/Abstract] OR "multi-drug therap*"[Title/Abstract] OR "multidrug therap*"[Title/Abstract] OR "multiple drug therap*"[Title/abstract] OR "multiple drug treatment?"[Title/Abstract] OR "multiple pharmacotherapy"[Title/Abstract] OR poly-pharma*[Title/Abstract] OR polypharma*[Title/Abstract] OR polypragma*[Title/Abstract] OR "inappropriate drug*"[Title/Abstract] OR "inappropriate medic*"[Title/Abstract] OR inappropriate prescri*[Title/Abstract] OR drug-related[Title/Abstract] OR "drug related"[Title/Abstract] OR drugrelated[Title/Abstract] OR prescript*[Title/Abstract] | 304250 |
| #5 | problem*[Title/Abstract] OR "side effects"[Title/Abstract] OR adverse[Title/Abstract] OR mortality[Title/Abstract] OR hospitali*[Title/Abstract] OR costs[Title/Abstract] | 2879278 |
| #6 | #4 AND #5 | 109173 |
| #7 | #3 AND #6 | **2308 references** |
| #8 | #7 Filters: English, from 2000 – 2021 | **1862 references** |
| #9 | #7 Filters: English, from 2010 – 2021 | 1363 references |
| **Embase** |  |  |
| #1 | 'dementia'/exp OR 'alzheimer disease'/exp OR 'huntington chorea'/exp OR 'frontotemporal dementia'/exp OR 'diffuse lewy body disease'/exp OR dement*:ab,ti OR alzheimer*:ab,ti OR huntington*:ab,ti OR 'frontotemporal disease*':ab,ti OR 'lewy body':ab,ti OR lewybody:ab,ti OR 'severe cognitive impairment':ab,ti | 446326 references |
| #2 | 'drug therap*':ab,ti OR 'drug treatment?':ab,ti OR 'medicament therap*':ab,ti OR 'medicament treatment?':ab,ti OR medication?:ab,ti OR 'medicinal therapy':ab,ti OR 'medicinal treatment':ab,ti OR 'pharmaceutical therap*':ab,ti OR 'pharmaceutical treatment?':ab,ti OR 'pharmaco therap*':ab,ti OR 'pharmaco treatment?':ab,ti OR 'pharmacological therap*':ab,ti OR 'pharmacological treatment?':ab,ti OR pharmacotherap*:ab,ti OR pharmacotreatment?:ab,ti OR 'multi-drug therap*':ab,ti OR 'multidrug therap*':ab,ti OR 'multiple drug therap*':ab,ti OR 'multiple drug treatment?':ab,ti OR 'multiple pharmacotherapy':ab,ti OR 'poly pharma*':ab,ti OR polypharma*:ab,ti OR polypragma*:ab,ti OR 'inappropriate drug*':ab,ti OR 'inappropriate medic*':ab,ti OR 'inappropriate prescri*':ab,ti OR 'drug related':ab,ti OR drugrelated:ab,ti OR prescript*:ab,ti | 569705 |
| #3 | problem*:ab,ti OR 'side effects':ab,ti OR adverse:ab,ti OR mortality:ab,ti OR hospitali*:ab,ti OR costs:ab,ti | 4029979 |
| #4 | #2 AND #3 | 203075 |
| #5 | #1 AND #4 | **5989 references** |
| #6 | #5 AND (2000:py OR 2001:py OR 2002:py OR 2003:py OR 2004:py OR 2005:py OR 2006:py OR 2007:py OR 2008:py OR 2009:py OR 2010:py OR 2011:py OR 2012:py OR 2013:py OR 2014:py OR 2015:py OR 2016:py OR 2017:py OR 2018:py OR 2019:py OR 2020:py OR 2021:py) AND [embase]/lim NOT ([embase]/lim AND [medline]/lim) | 2894 |
| #7 | 'conference abstract' | 4082452 |
| #8 | #6 NOT #7 | **821 references** |
| #9 | #8 AND AND (2010:py OR 2011:py OR 2012:py OR 2013:py OR 2014:py OR 2015:py OR 2016:py OR 2017:py OR 2018:py OR 2019:py OR 2020:py OR 2021:py) | 569 references |
| **Web of science** | **LANGUAGE:(English) AND DOCUMENT TYPES:(Article) Timespan=2000-2021** |  |
| #1 | (TI=(dement* OR alzheimer* OR huntington* OR "frontotemporal diseas*" OR "lewy body*" OR lewy-body* OR lewybody*)) | 70553 |
| #2 | (AB=(dement* OR alzheimer* OR huntington* OR "frontotemporal diseas*" OR "lewy body*" OR lewy-body* OR lewybody*)) | 142387 |
| #3 | (TS=(Dementia OR Alzheimer disease OR huntington disease OR frontotemporal disease OR frontotemporal dementia OR lewy body disease) | 173490 |
| #4 | #1 OR #2 OR #3 | 176123 |
| #5 | (TI=("drug therap*" OR "drug treatment?" OR "medicament therap*" OR "medicament treatment?"OR medication? OR "medicinal therapy" OR "medicinal treatment" OR "pharmaceutical therap*" OR "pharmaceutical treatment?" OR "pharmaco therap*" OR "pharmaco treatment?" OR "pharmacological therap*" OR "pharmacological treatment?" OR pharmacotherap* OR pharmacotreatment? OR "multi-drug therap*" OR "multidrug therap*" OR "multiple drug therap*" OR "multiple drug treatment?" OR "multiple pharmacotherapy" OR "poly pharma*" OR polypharma* OR polypragma* OR "inappropriate drug*" OR "inappropriate medic*" OR "inappropriate prescri*" OR "drug related" OR drugrelated OR prescript*)) | 25187 |
| #6 | (AB=("drug therap*" OR "drug treatment?" OR "medicament therap*" OR "medicament treatment?"OR medication? OR "medicinal therapy" OR "medicinal treatment" OR "pharmaceutical therap*" OR "pharmaceutical treatment?" OR "pharmaco therap*" OR "pharmaco treatment?" OR "pharmacological therap*" OR "pharmacological treatment?" OR pharmacotherap* OR pharmacotreatment? OR "multi-drug therap*" OR "multidrug therap*" OR "multiple drug therap*" OR "multiple drug treatment?" OR "multiple pharmacotherapy" OR "poly pharma*" OR polypharma* OR polypragma* OR "inappropriate drug*" OR "inappropriate medic*" OR "inappropriate prescri*" OR "drug related" OR drugrelated OR prescript*)) | 197550 |
| #7 | (TS=(pharmacotherapy)) | 17244 |
| #8 | #5 OR #6 OR #7 | 200866 |
| #9  #10 | (TI=(problem* OR "side effects" OR adverse OR mortality OR hospitali* OR costs) )  (AB=(problem* OR "side effects" OR adverse OR mortality OR hospitali* OR costs)) | 471857  3557801 |
| #11 | #9 OR #10 | 3624494 |
| #12 | #8 AND #11 | 70346 |
| #13 | #4 AND #12 | **1779 references** |
| #14 | #13  Refined by: PUBLICATION YEARS: ( 2021 OR 2015 OR 2020 OR 2014 OR 2019 OR 2013 OR 2018 OR 2012 OR 2017 OR 2011 OR 2016 OR 2010 ) | 1310 |
| **CINAHL** |  |  |
| #1 | (MH "Dementia+") OR (MH "Frontotemporal Lobar Degeneration") OR (MM "Alzheimer's Disease") OR (MM "Lewy Body Disease") OR (MH "Frontotemporal Dementia+") OR (MH Huntington's disease) | 77950 |
| #2 | TI ( dement* OR huntington* OR alzheimer* OR "frontotemporal disease* OR "lewy-body disease" OR "lewy body disease" OR "lewybody disease" ) OR AB ( dement* OR huntington* OR alzheimer* OR "frontotemporal disease* OR "lewy-body disease" OR "lewy body disease" OR "lewybody disease" ) | 81466 |
| #3 | #1 OR #2 | 100185 |
| #4 | TI ( "drug therap*" OR "drug treatment?" OR "medicament therap*" OR "medicament treatment?"OR medication? OR "medicinal therapy" OR "medicinal treatment" OR "pharmaceutical therap*" OR "pharmaceutical treatment?" OR "pharmaco therap*" OR "pharmaco treatment?" OR "pharmacological therap*" OR "pharmacological treatment?" OR pharmacotherap* OR pharmacotreatment? OR "multi-drug therap*" OR "multidrug therap*" OR "multiple drug therap*" OR "multiple drug treatment?" OR "multiple pharmacotherapy" OR "poly pharma*" OR polypharma* OR polypragma* OR "inappropriate drug*" OR "inappropriate medic*" OR "inappropriate prescri*" OR "drug related" OR drugrelated OR prescript* ) OR AB ( "drug therap*" OR "drug treatment?" OR "medicament therap*" OR "medicament treatment?"OR medication? OR "medicinal therapy" OR "medicinal treatment" OR "pharmaceutical therap*" OR "pharmaceutical treatment?" OR "pharmaco therap*" OR "pharmaco treatment?" OR "pharmacological therap*" OR "pharmacological treatment?" OR pharmacotherap* OR pharmacotreatment? OR "multi-drug therap*" OR "multidrug therap*" OR "multiple drug therap*" OR "multiple drug treatment?" OR "multiple pharmacotherapy" OR "poly pharma*" OR polypharma* OR polypragma* OR "inappropriate drug*" OR "inappropriate medic*" OR "inappropriate prescri*" OR "drug related" OR drugrelated OR prescript* ) | 193636 |
| #5 | (MH "Polypharmacy+") | 4806 |
| #6 | #4 OR #5 | 195103 |
| #7 | TI ( problem* OR "side effects" OR adverse OR mortality OR hospitali* OR costs ) OR AB ( problem* OR "side effects" OR adverse OR mortality OR hospitali* OR costs ) | 816477 |
| #8 | #6 AND #7 | 64694 |
| #9 | #3 AND #8 | **1879 references** |
| #10 | #9 Limiters - Published Date: 20000101-20211231 | **1797 references** |
| #11 | #9 Limiters - Published Date: 20100101-20211231 | 1289 references |

| **2021-05-07** |  |  |
| --- | --- | --- |
| **Cochrane** |  |  |
| #1 | (dement* OR alzheimer* OR huntington* OR "frontotemporal diseas*" OR "lewy body*" OR lewy-body* OR lewybody*):ti,ab,kw | 21013 |
| #2 | "drug therap*" OR "drug treatment" OR "medicament therap*" OR "medicament treatment" OR medication OR "medicinal therap" OR "medicinal treatment" OR "pharmaceutical therap*" OR "pharmaceutical treatment" OR "pharmaco therap*" OR "pharmaco treatment" OR "pharmacological therap*" OR "Pharmacological treatment" OR pharmacotherap* OR pharmacotreatment OR "multi-drug therap*" OR "multiple drug therap*" OR "multiple drug treatment" OR "multiple pharmacotherap*" OR "poly pharma*" OR polypharma* OR poly-pharma* OR polypragma* OR inappropriate drug* OR inappropriate medic* OR prescri* OR "drug related" OR drugrelated OR drug-related | 136459 |
| #3 | (problem* OR "side effects" OR adverse OR mortality OR hospitali* OR costs):ti,ab,kw | 543489 |
| #4 | #2 AND #3 | 69501 |
| #5 | #1 AND #4 | 1340 references |
| #6 | #5 with Cochrane Library publication date from Jan 2000 to Dec 2021 | **1281 references**  **(170 reviews, 1 protocol,1110 trials)** |
| #7 | #5 with Cochrane Library publication date from Jan 2010 to Dec 2021 | 1132 references (123 reviews, 1 protocol, 1008 trials) |
| **Duplicates**  **2000-**  **Before duplicate control: 7540 After duplicate control: 4643**  **Duplicates 2010-**  **Before duplicate control: 5100**  **After duplicate control: 3066** |  |  |
| **PubMed** |  |  |
| #1 | "Dementia"[MeSH Terms] OR "Alzheimer Disease"[MeSH Terms] OR "Huntington Disease"[MeSH Terms] OR "Frontotemporal Dementia"[MeSH Terms] OR "Lewy Body Disease"[MeSH Terms] | 217,746 |
| #2 | "dement*"[Title/Abstract] OR "huntington*"[Title/Abstract] OR "alzheimer*"[Title/Abstract] OR "frontotemporal diseas*"[Title/Abstract] OR "lewy body*"[Title/Abstract] OR "lewy body*"[Title/Abstract] OR "lewybody*"[Title/Abstract] OR "severe cognitive impairment"[Title/Abstract] | 321,955 |
| #3 | "Dementia"[MeSH Terms] OR "Alzheimer Disease"[MeSH Terms] OR "Huntington Disease"[MeSH Terms] OR "Frontotemporal Dementia"[MeSH Terms] OR "Lewy Body Disease"[MeSH Terms] OR "dement*"[Title/Abstract] OR "huntington*"[Title/Abstract] OR "alzheimer*"[Title/Abstract] OR "frontotemporal diseas*"[Title/Abstract] OR "lewy body*"[Title/Abstract] OR "lewy body*"[Title/Abstract] OR "lewybody*"[Title/Abstract] OR "severe cognitive impairment"[Title/Abstract] | 352,322 |
| #4 | "drug therap*"[Title/Abstract] OR "drug treatment"[Title/Abstract] OR "medicament therap*"[Title/Abstract] OR "medicament treatment"[Title/Abstract] OR "medication"[Title/Abstract] OR "medicinal therapy"[Title/Abstract] OR "medicinal treatment"[Title/Abstract] OR "pharmaceutical therap*"[Title/Abstract] OR "pharmaceutical treatment"[Title/Abstract] OR "pharmaco therap*"[Title/Abstract] OR "pharmaco-treatment"[Title/Abstract] OR "pharmacological therap*"[Title/Abstract] OR "pharmacological treatment"[Title/Abstract] OR "pharmacotherap*"[Title/Abstract] OR "pharmacotreatment"[Title/Abstract] OR "multi drug therap*"[Title/Abstract] OR "multidrug therap*"[Title/Abstract] OR "multiple drug therap*"[Title/Abstract] OR "multiple drug treatment"[Title/Abstract] OR "multiple pharmacotherapy"[Title/Abstract] OR "poly pharma*"[Title/Abstract] OR "polypharma*"[Title/Abstract] OR "polypragma*"[Title/Abstract] OR "inappropriate drug*"[Title/Abstract] OR "inappropriate medic*"[Title/Abstract] OR "inappropriate prescri*"[Title/Abstract] OR "drug-related"[Title/Abstract] OR "drug-related"[Title/Abstract] OR "drugrelated"[Title/Abstract] OR "prescript*"[Title/Abstract] | 590,070 |
| #5 | "problem*"[Title/Abstract] OR "side effects"[Title/Abstract] OR "adverse"[Title/Abstract] OR "mortality"[Title/Abstract] OR "hospitali*"[Title/Abstract] OR "costs"[Title/Abstract] | 3,625,811 |
| #6 | ("drug therap*"[Title/Abstract] OR "drug treatment"[Title/Abstract] OR "medicament therap*"[Title/Abstract] OR "medicament treatment"[Title/Abstract] OR "medication"[Title/Abstract] OR "medicinal therapy"[Title/Abstract] OR "medicinal treatment"[Title/Abstract] OR "pharmaceutical therap*"[Title/Abstract] OR "pharmaceutical treatment"[Title/Abstract] OR "pharmaco therap*"[Title/Abstract] OR "pharmaco-treatment"[Title/Abstract] OR "pharmacological therap*"[Title/Abstract] OR "pharmacological treatment"[Title/Abstract] OR "pharmacotherap*"[Title/Abstract] OR "pharmacotreatment"[Title/Abstract] OR "multi drug therap*"[Title/Abstract] OR "multidrug therap*"[Title/Abstract] OR "multiple drug therap*"[Title/Abstract] OR "multiple drug treatment"[Title/Abstract] OR "multiple pharmacotherapy"[Title/Abstract] OR "poly pharma*"[Title/Abstract] OR "polypharma*"[Title/Abstract] OR "polypragma*"[Title/Abstract] OR "inappropriate drug*"[Title/Abstract] OR "inappropriate medic*"[Title/Abstract] OR "inappropriate prescri*"[Title/Abstract] OR "drug-related"[Title/Abstract] OR "drug-related"[Title/Abstract] OR "drugrelated"[Title/Abstract] OR "prescript*"[Title/Abstract]) AND ("problem*"[Title/Abstract] OR "side effects"[Title/Abstract] OR "adverse"[Title/Abstract] OR "mortality"[Title/Abstract] OR "hospitali*"[Title/Abstract] OR "costs"[Title/Abstract]) | 187,627 |
| #7 | ("drug therap*"[Title/Abstract] OR "drug treatment"[Title/Abstract] OR "medicament therap*"[Title/Abstract] OR "medicament treatment"[Title/Abstract] OR "medication"[Title/Abstract] OR "medicinal therapy"[Title/Abstract] OR "medicinal treatment"[Title/Abstract] OR "pharmaceutical therap*"[Title/Abstract] OR "pharmaceutical treatment"[Title/Abstract] OR "pharmaco therap*"[Title/Abstract] OR "pharmaco-treatment"[Title/Abstract] OR "pharmacological therap*"[Title/Abstract] OR "pharmacological treatment"[Title/Abstract] OR "pharmacotherap*"[Title/Abstract] OR "pharmacotreatment"[Title/Abstract] OR "multi drug therap*"[Title/Abstract] OR "multidrug therap*"[Title/Abstract] OR "multiple drug therap*"[Title/Abstract] OR "multiple drug treatment"[Title/Abstract] OR "multiple pharmacotherapy"[Title/Abstract] OR "poly pharma*"[Title/Abstract] OR "polypharma*"[Title/Abstract] OR "polypragma*"[Title/Abstract] OR "inappropriate drug*"[Title/Abstract] OR "inappropriate medic*"[Title/Abstract] OR "inappropriate prescri*"[Title/Abstract] OR "drug-related"[Title/Abstract] OR "drug-related"[Title/Abstract] OR "drugrelated"[Title/Abstract] OR "prescript*"[Title/Abstract]) AND ("problem*"[Title/Abstract] OR "side effects"[Title/Abstract] OR "adverse"[Title/Abstract] OR "mortality"[Title/Abstract] OR "hospitali*"[Title/Abstract] OR "costs"[Title/Abstract]) | 187,627 |
| #8 | ("Dementia"[MeSH Terms] OR "Alzheimer Disease"[MeSH Terms] OR "Huntington Disease"[MeSH Terms] OR "Frontotemporal Dementia"[MeSH Terms] OR "Lewy Body Disease"[MeSH Terms] OR ("dement*"[Title/Abstract] OR "huntington*"[Title/Abstract] OR "alzheimer*"[Title/Abstract] OR "frontotemporal diseas*"[Title/Abstract] OR "lewy body*"[Title/Abstract] OR "lewy body*"[Title/Abstract] OR "lewybody*"[Title/Abstract] OR "severe cognitive impairment"[Title/Abstract])) AND (("drug therap*"[Title/Abstract] OR "drug treatment"[Title/Abstract] OR "medicament therap*"[Title/Abstract] OR "medicament treatment"[Title/Abstract] OR "medication"[Title/Abstract] OR "medicinal therapy"[Title/Abstract] OR "medicinal treatment"[Title/Abstract] OR "pharmaceutical therap*"[Title/Abstract] OR "pharmaceutical treatment"[Title/Abstract] OR "pharmaco therap*"[Title/Abstract] OR "pharmaco-treatment"[Title/Abstract] OR "pharmacological therap*"[Title/Abstract] OR "pharmacological treatment"[Title/Abstract] OR "pharmacotherap*"[Title/Abstract] OR "pharmacotreatment"[Title/Abstract] OR "multi drug therap*"[Title/Abstract] OR "multidrug therap*"[Title/Abstract] OR "multiple drug therap*"[Title/Abstract] OR "multiple drug treatment"[Title/Abstract] OR "multiple pharmacotherapy"[Title/Abstract] OR "poly pharma*"[Title/Abstract] OR "polypharma*"[Title/Abstract] OR "polypragma*"[Title/Abstract] OR "inappropriate drug*"[Title/Abstract] OR "inappropriate medic*"[Title/Abstract] OR "inappropriate prescri*"[Title/Abstract] OR "drug-related"[Title/Abstract] OR "drug-related"[Title/Abstract] OR "drugrelated"[Title/Abstract] OR "prescript*"[Title/Abstract]) AND ("problem*"[Title/Abstract] OR "side effects"[Title/Abstract] OR "adverse"[Title/Abstract] OR "mortality"[Title/Abstract] OR "hospitali*"[Title/Abstract] OR "costs"[Title/Abstract])) | 4,077 |
| #9 | (("Dementia"[MeSH Terms] OR "Alzheimer Disease"[MeSH Terms] OR "Huntington Disease"[MeSH Terms] OR "Frontotemporal Dementia"[MeSH Terms] OR "Lewy Body Disease"[MeSH Terms] OR ("dement*"[Title/Abstract] OR "huntington*"[Title/Abstract] OR "alzheimer*"[Title/Abstract] OR "frontotemporal diseas*"[Title/Abstract] OR "lewy body*"[Title/Abstract] OR "lewy body*"[Title/Abstract] OR "lewybody*"[Title/Abstract] OR "severe cognitive impairment"[Title/Abstract])) AND (("drug therap*"[Title/Abstract] OR "drug treatment"[Title/Abstract] OR "medicament therap*"[Title/Abstract] OR "medicament treatment"[Title/Abstract] OR "medication"[Title/Abstract] OR "medicinal therapy"[Title/Abstract] OR "medicinal treatment"[Title/Abstract] OR "pharmaceutical therap*"[Title/Abstract] OR "pharmaceutical treatment"[Title/Abstract] OR "pharmaco therap*"[Title/Abstract] OR "pharmaco-treatment"[Title/Abstract] OR "pharmacological therap*"[Title/Abstract] OR "pharmacological treatment"[Title/Abstract] OR "pharmacotherap*"[Title/Abstract] OR "pharmacotreatment"[Title/Abstract] OR "multi drug therap*"[Title/Abstract] OR "multidrug therap*"[Title/Abstract] OR "multiple drug therap*"[Title/Abstract] OR "multiple drug treatment"[Title/Abstract] OR "multiple pharmacotherapy"[Title/Abstract] OR "poly pharma*"[Title/Abstract] OR "polypharma*"[Title/Abstract] OR "polypragma*"[Title/Abstract] OR "inappropriate drug*"[Title/Abstract] OR "inappropriate medic*"[Title/Abstract] OR "inappropriate prescri*"[Title/Abstract] OR "drug-related"[Title/Abstract] OR "drug-related"[Title/Abstract] OR "drugrelated"[Title/Abstract] OR "prescript*"[Title/Abstract]) AND ("problem*"[Title/Abstract] OR "side effects"[Title/Abstract] OR "adverse"[Title/Abstract] OR "mortality"[Title/Abstract] OR "hospitali*"[Title/Abstract] OR "costs"[Title/Abstract]))) AND (2023/6/30:2024/8/14[pdat]) | 364 |
| #10 | (("Dementia"[MeSH Terms] OR "Alzheimer Disease"[MeSH Terms] OR "Huntington Disease"[MeSH Terms] OR "Frontotemporal Dementia"[MeSH Terms] OR "Lewy Body Disease"[MeSH Terms] OR ("dement*"[Title/Abstract] OR "huntington*"[Title/Abstract] OR "alzheimer*"[Title/Abstract] OR "frontotemporal diseas*"[Title/Abstract] OR "lewy body*"[Title/Abstract] OR "lewy body*"[Title/Abstract] OR "lewybody*"[Title/Abstract] OR "severe cognitive impairment"[Title/Abstract])) AND (("drug therap*"[Title/Abstract] OR "drug treatment"[Title/Abstract] OR "medicament therap*"[Title/Abstract] OR "medicament treatment"[Title/Abstract] OR "medication"[Title/Abstract] OR "medicinal therapy"[Title/Abstract] OR "medicinal treatment"[Title/Abstract] OR "pharmaceutical therap*"[Title/Abstract] OR "pharmaceutical treatment"[Title/Abstract] OR "pharmaco therap*"[Title/Abstract] OR "pharmaco-treatment"[Title/Abstract] OR "pharmacological therap*"[Title/Abstract] OR "pharmacological treatment"[Title/Abstract] OR "pharmacotherap*"[Title/Abstract] OR "pharmacotreatment"[Title/Abstract] OR "multi drug therap*"[Title/Abstract] OR "multidrug therap*"[Title/Abstract] OR "multiple drug therap*"[Title/Abstract] OR "multiple drug treatment"[Title/Abstract] OR "multiple pharmacotherapy"[Title/Abstract] OR "poly pharma*"[Title/Abstract] OR "polypharma*"[Title/Abstract] OR "polypragma*"[Title/Abstract] OR "inappropriate drug*"[Title/Abstract] OR "inappropriate medic*"[Title/Abstract] OR "inappropriate prescri*"[Title/Abstract] OR "drug-related"[Title/Abstract] OR "drug-related"[Title/Abstract] OR "drugrelated"[Title/Abstract] OR "prescript*"[Title/Abstract]) AND ("problem*"[Title/Abstract] OR "side effects"[Title/Abstract] OR "adverse"[Title/Abstract] OR "mortality"[Title/Abstract] OR "hospitali*"[Title/Abstract] OR "costs"[Title/Abstract]))) AND ((2023/6/30:2024/8/14[pdat]) AND (english[Filter])) | 356 |
| Embase |  |  |
| #1 | 'dementia'/exp OR 'dementia' OR 'alzheimer disease'/exp OR 'alzheimer disease' OR 'huntington chorea'/exp OR 'huntington chorea' OR 'frontotemporal dementia'/exp OR 'frontotemporal dementia' OR 'diffuse lewy body disease'/exp OR 'diffuse lewy body disease' OR dement*:ab,ti OR alzheimer*:ab,ti OR huntington*:ab,ti OR 'frontotemporal disease*':ab,ti OR 'lewy body':ab,ti OR lewybody:ab,ti OR 'severe cognitive impairment':ab,ti | 572923 |
| #2 | 'drug therap*':ab,ti OR 'drug treatment?':ab,ti OR 'medicament therap*':ab,ti OR 'medicament treatment?':ab,ti OR medication?:ab,ti OR 'medicinal therapy':ab,ti OR 'medicinal treatment':ab,ti OR 'pharmaceutical therap*':ab,ti OR 'pharmaceutical treatment?':ab,ti OR 'pharmaco therap*':ab,ti OR 'pharmaco treatment?':ab,ti OR 'pharmacological therap*':ab,ti OR 'pharmacological treatment?':ab,ti OR pharmacotherap*:ab,ti OR pharmacotreatment?:ab,ti OR 'multi-drug therap*':ab,ti OR 'multidrug therap*':ab,ti OR 'multiple drug therap*':ab,ti OR 'multiple drug treatment?':ab,ti OR 'multiple pharmacotherapy':ab,ti OR 'poly pharma*':ab,ti OR polypharma*:ab,ti OR polypragma*:ab,ti OR 'inappropriate drug*':ab,ti OR 'inappropriate medic*':ab,ti OR 'inappropriate prescri*':ab,ti OR 'drug related':ab,ti OR drugrelated:ab,ti OR prescript*:ab,ti | 731626 |
| #3 | problem*:ab,ti OR 'side effects':ab,ti OR adverse:ab,ti OR mortality:ab,ti OR hospitali*:ab,ti OR costs:ab,ti | 5082440 |
| #4  #5 | #2 AND #3  #1 AND #4 | 263295  7914 |
| #6 | #1 AND #4 AND [2010-2024]/py | 6331 |
| #7 | #1 AND #4 AND [2010-2024]/py AND [01-07-2023]/sd NOT [15-08-2024]/sd | 731 |
| #8 | 'dementia'/exp OR 'dementia' OR 'alzheimer disease'/exp OR 'alzheimer disease' OR 'huntington chorea'/exp OR 'huntington chorea' OR 'frontotemporal dementia'/exp OR 'frontotemporal dementia' OR 'diffuse lewy body disease'/exp OR 'diffuse lewy body disease' OR dement*:ab,ti OR alzheimer*:ab,ti OR huntington*:ab,ti OR 'frontotemporal disease*':ab,ti OR 'lewy body':ab,ti OR lewybody:ab,ti OR 'severe cognitive impairment':ab,ti | 572923 |
| #9 | 'drug therap*':ab,ti OR 'drug treatment?':ab,ti OR 'medicament therap*':ab,ti OR 'medicament treatment?':ab,ti OR medication?:ab,ti OR 'medicinal therapy':ab,ti OR 'medicinal treatment':ab,ti OR 'pharmaceutical therap*':ab,ti OR 'pharmaceutical treatment?':ab,ti OR 'pharmaco therap*':ab,ti OR 'pharmaco treatment?':ab,ti OR 'pharmacological therap*':ab,ti OR 'pharmacological treatment?':ab,ti OR pharmacotherap*:ab,ti OR pharmacotreatment?:ab,ti OR 'multi-drug therap*':ab,ti OR 'multidrug therap*':ab,ti OR 'multiple drug therap*':ab,ti OR 'multiple drug treatment?':ab,ti OR 'multiple pharmacotherapy':ab,ti OR 'poly pharma*':ab,ti OR polypharma*:ab,ti OR polypragma*:ab,ti OR 'inappropriate drug*':ab,ti OR 'inappropriate medic*':ab,ti OR 'inappropriate prescri*':ab,ti OR 'drug related':ab,ti OR drugrelated:ab,ti OR prescript*:ab,ti | 731626 |
| #10 | problem*:ab,ti OR 'side effects':ab,ti OR adverse:ab,ti OR mortality:ab,ti OR hospitali*:ab,ti OR costs:ab,ti | 5082440 |
| #11 | #9 AND #10 | 263295 |
| #12 | #8 AND #11 | 7914 |
| #13 | #8 AND #11 AND [2010-2024]/py | 6331 |
| #14 | #8 AND #11 AND [2010-2024]/py AND [01-07-2023]/sd NOT [15-08-2024]/sd | 731 |
| #15 | 'conference abstract' | 5209886 |
| #16 | #14 NOT #15 | 559 |
| Web of Science |  |  |
| #1 | "TI=((dement* OR alzheimer* OR huntington* OR ""frontotemporal diseas*"" OR ""lewy body*"" OR lewy-body* OR lewybody*) )Timespan: 2023-07-01 to 2024-08-15 " | 12952 |
| #2 | "AB=(dement* OR alzheimer* OR huntington* OR ""frontotemporal diseas*"" OR ""lewy body*"" OR lewy-body* OR lewybody*)Timespan: 2023-07-01 to 2024-08-15 " | 20089 |
| #3 | "(TS=(Dementia OR Alzheimer disease OR huntington disease OR frontotemporal disease OR frontotemporal dementia OR lewy body disease) ) Timespan: 2023-07-01 to 2024-08-15 " | 26293 |
| #4 | "#1 OR #2 OR #3 Timespan: 2023-07-01 to 2024-08-15 " | 26671 |
| #5 | "(TI=(""drug therap*"" OR ""drug treatment?"" OR ""medicament therap*"" OR ""medicament treatment?""OR medication? OR ""medicinal therapy"" OR ""medicinal treatment"" OR ""pharmaceutical therap*"" OR ""pharmaceutical treatment?"" OR ""pharmaco therap*"" OR ""pharmaco treatment?"" OR ""pharmacological therap*"" OR ""pharmacological treatment?"" OR pharmacotherap* OR pharmacotreatment? OR ""multi-drug therap*"" OR ""multidrug therap*"" OR ""multiple drug therap*"" OR ""multiple drug treatment?"" OR ""multiple pharmacotherapy"" OR ""poly pharma*"" OR polypharma* OR polypragma* OR ""inappropriate drug*"" OR ""inappropriate medic*"" OR ""inappropriate prescri*"" OR ""drug related"" OR drugrelated OR prescript*))Timespan: 2023-07-01 to 2024-08-15 " | 5188 |
| #6 | "(AB=(""drug therap*"" OR ""drug treatment?"" OR ""medicament therap*"" OR ""medicament treatment?""OR medication? OR ""medicinal therapy"" OR ""medicinal treatment"" OR ""pharmaceutical therap*"" OR ""pharmaceutical treatment?"" OR ""pharmaco therap*"" OR ""pharmaco treatment?"" OR ""pharmacological therap*"" OR ""pharmacological treatment?"" OR pharmacotherap* OR pharmacotreatment? OR ""multi-drug therap*"" OR ""multidrug therap*"" OR ""multiple drug therap*"" OR ""multiple drug treatment?"" OR ""multiple pharmacotherapy"" OR ""poly pharma*"" OR polypharma* OR polypragma* OR ""inappropriate drug*"" OR ""inappropriate medic*"" OR ""inappropriate prescri*"" OR ""drug related"" OR drugrelated OR prescript*)) Timespan: 2023-07-01 to 2024-08-15 " | 31977 |
| #7 | "(TS=(pharmacotherapy)) Timespan: 2023-07-01 to 2024-08-15 " | 2645 |
| #8 | "#5 OR #6 OR #7 Timespan: 2023-07-01 to 2024-08-15 " | 34213 |
| #9 | "(TI=(problem* OR ""side effects"" OR adverse OR mortality OR hospitali* OR costs)) Timespan: 2023-07-01 to 2024-08-15 " | 61302 |
| #10 | "(AB=(problem* OR ""side effects"" OR adverse OR mortality OR hospitali* OR costs)) Timespan: 2023-07-01 to 2024-08-15 " | 499493 |
| #11 | "#9 OR #10 Timespan: 2023-07-01 to 2024-08-15 " | 514414 |
| #12 | "#8 AND #11 Timespan: 2023-07-01 to 2024-08-15 " | 12207 |
| #13 | "#4 AND #12 Timespan: 2023-07-01 to 2024-08-15 " | 340 |
| #14 | "#4 AND #12 and Article (Document Types) and English (Languages) Timespan: 2023-07-01 to 2024-08-15 " | 229 |
| #15 | "#4 AND #12 and Article (Document Types) and English (Languages) and Article (Document Types) Timespan: 2023-07-01 to 2024-08-15 " | 229 |
| CINAHL |  |  |
| #1 | (MH "Dementia+") OR (MH "Frontotemporal Lobar Degeneration") OR (MM "Alzheimer's Disease") OR (MM "Lewy Body Disease") OR (MH "Frontotemporal Dementia+") OR (MH Huntington's disease) | 88,524 |
| #2 | TI ( dement* OR huntington* OR alzheimer* OR "frontotemporal disease* OR "lewy-body disease" OR "lewy body disease" OR "lewybody disease" ) OR AB ( dement* OR huntington* OR alzheimer* OR "frontotemporal disease* OR "lewy-body disease" OR "lewy body disease" OR "lewybody disease" ) | 69,860 |
| #3 | (TI ( dement* OR huntington* OR alzheimer* OR "frontotemporal disease* OR "lewy-body disease" OR "lewy body disease" OR "lewybody disease" ) OR AB ( dement* OR huntington* OR alzheimer* OR "frontotemporal disease* OR "lewy-body disease" OR "lewy body disease" OR "lewybody disease" )) AND (S1 OR S2) | 69,860 |
| #4 | TI ( "drug therap*" OR "drug treatment?" OR "medicament therap*" OR "medicament treatment?"OR medication? OR "medicinal therapy" OR "medicinal treatment" OR "pharmaceutical therap*" OR "pharmaceutical treatment?" OR "pharmaco therap*" OR "pharmaco treatment?" OR "pharmacological therap*" OR "pharmacological treatment?" OR pharmacotherap* OR pharmacotreatment? OR "multi-drug therap*" OR "multidrug therap*" OR "multiple drug therap*" OR "multiple drug treatment?" OR "multiple pharmacotherapy" OR "poly pharma*" OR polypharma* OR polypragma* OR "inappropriate drug*" OR "inappropriate medic*" OR "inappropriate prescri*" OR "drug related" OR drugrelated OR prescript* ) OR AB ( "drug therap*" OR "drug treatment?" OR "medicament therap*" OR "medicament treatment?"OR medication? OR "medicinal therapy" OR "medicinal treatment" OR "pharmaceutical therap*" OR "pharmaceutical treatment?" OR "pharmaco therap*" OR "pharmaco treatment?" OR "pharmacological therap*" OR "pharmacological treatment?" OR pharmacotherap* OR pharmacotreatment? OR "multi-drug therap*" OR "multidrug therap*" OR "multiple drug therap*" OR "multiple drug treatment?" OR "multiple pharmacotherapy" OR "poly pharma*" OR polypharma* OR polypragma* OR "inappropriate drug*" OR "inappropriate medic*" OR "inappropriate prescri*" OR "drug related" OR drugrelated OR prescript* ) | 229,140 |
| #5 | (MH "Polypharmacy+") | 6,254 |
| #6 | S4 OR S5 | 230,892 |
| #7 | TI ( problem* OR "side effects" OR adverse OR mortality OR hospitali* OR costs ) OR AB ( problem* OR "side effects" OR adverse OR mortality OR hospitali* OR costs ) | 982,842 |
| #8 | S6 AND S7 | 76,880 |
| #9 | S3 AND S8 | 1,158 |
| #10 | S3 AND S8 | 49 |
| Cochrane |  |  |
| #1 | (dement* OR alzheimer* OR huntington* OR "frontotemporal diseas*" OR "lewy body*" OR lewy-body* OR lewybody*):ti,ab,kw (Word variations have been searched) | 27377 |
| #2 | "drug therap*" OR "drug treatment" OR "medicament therap*" OR "medicament treatment" OR medication OR "medicinal therap" OR "medicinal treatment" OR "pharmaceutical therap*" OR "pharmaceutical treatment" OR "pharmaco therap*" OR "pharmaco treatment" OR "pharmacological therap*" OR "Pharmacological treatment" OR pharmacotherap* OR pharmacotreatment OR "multi-drug therap*" OR "multiple drug therap*" OR "multiple drug treatment" OR "multiple pharmacotherap*" OR "poly pharma*" OR polypharma* OR poly-pharma* OR polypragma* OR inappropriate drug* OR inappropriate medic* OR prescri* OR "drug related" OR drugrelated OR drug-related | 173966 |
| #3 | ((problem* OR "side effects" OR adverse OR mortality OR hospitali* OR costs)):ti,ab,kw (Word variations have been searched) | 700734 |
| #4 | #2 AND #3 | 241534 |
| #5 | #1 AND #4 with Cochrane Library publication from 01 Jul 2023 to 14 Aug 2024 | 3617 (204 reviews, 3408 trials, 5 editorials)  2588 (137 reviews, 2446 trials, 5 editorials) (11 Jul 2023)  (8 reviews, 270 trials, 5 editorials) |

Table of Contents

[1 Supplementary Figures and Tables 1](#_Toc226219838)

[1.1 Supplementary Figures 1](#_Toc226219839)

Reference:

1. Schwertner E, Secnik J, Garcia-Ptacek S, Johansson B, Nagga K, Eriksdotter M, et al. Antipsychotic treatment associated with increased mortality risk in patients with dementia. A registry-based observational cohort study. Journal of the American Medical Directors Association. 2019;20(3):323-9. e2.

2. Koponen M, Taipale H, Lavikainen P, Tanskanen A, Tiihonen J, Tolppanen A-M, et al. Risk of mortality associated with antipsychotic monotherapy and polypharmacy among community-dwelling persons with Alzheimer’s disease. Journal of Alzheimer's Disease. 2017;56(1):107-18.

3. Dennis M, Shine L, John A, Marchant A, McGregor J, Lyons RA, et al. Risk of adverse outcomes for older people with dementia prescribed antipsychotic medication: a population based e-cohort study. Neurology and therapy. 2017;6(1):57-77.

4. Mueller C, John C, Perera G, Aarsland D, Ballard C, Stewart R. Antipsychotic use in dementia: the relationship between neuropsychiatric symptom profiles and adverse outcomes. European journal of epidemiology. 2021;36(1):89-101.

5. Virginie G, Maryse L-M, Nicola C, Christelle C, Jean-Louis M, Bruno V, et al. Antipsychotic Use and Mortality Risk in Community-Dwelling Alzheimer’s Disease Patients: Evidence for a Role of Dementia Severity. Current Alzheimer Research. 2012;9(9):1106-16.

6. Nørgaard A, Jensen-Dahm C, Wimberley T, Svendsen JH, Ishtiak-Ahmed K, Laursen TM, et al. Effect of antipsychotics on mortality risk in patients with dementia with and without comorbidities. J Am Geriatr Soc. 2022;70(4):1169-79.

7. Brännström J, Boström G, Rosendahl E, Nordström P, Littbrand H, Lövheim H, et al. Psychotropic drug use and mortality in old people with dementia: investigating sex differences. BMC Pharmacology and Toxicology. 2017;18(1):1-11.

8. Hamedani AG, Weintraub D, Willis AW. Hallucinations, Antipsychotic Use, and Mortality in Older Adults with Dementia: Retrospective Cohort Study of Two Medicare-Linked National Health Surveys. Drugs Aging. 2022;39(12):967-74.

9. Chu C-S, Li W-R, Huang K-L, Su P-Y, Lin C-H, Lan T-H. The use of antipsychotics is associated with lower mortality in patients with Alzheimer’s disease: A nationwide population-based nested case-control study in Taiwan. Journal of Psychopharmacology. 2018;32(11):1182-90.

10. Harrison SL, Sluggett JK, Lang C, Whitehead C, Crotty M, Corlis M, et al. Initiation of antipsychotics after moving to residential aged care facilities and mortality: a national cohort study. Aging clinical and experimental research. 2021;33(1):95.

11. Jennum P, Baandrup L, Ibsen R, Kjellberg J. Increased all-cause mortality with use of psychotropic medication in dementia patients and controls: a population-based register study. European neuropsychopharmacology. 2015;25(11):1906-13.

12. Musicco M, Palmer K, Russo A, Caltagirone C, Adorni F, Pettenati C, et al. Association between prescription of conventional or atypical antipsychotic drugs and mortality in older persons with Alzheimer’s disease. Dementia and geriatric cognitive disorders. 2011;31(3):218-24.

13. Nerius M, Johnell K, Garcia-Ptacek S, Eriksdotter M, Haenisch B, Doblhammer G. The Impact of Antipsychotic Drugs on Long-term Care, Nursing Home Admission, and Death in Dementia Patients. J Gerontol A Biol Sci Med Sci. 2018;73(10):1396-402.

14. Sultana J, Chang C-K, Hayes R, Broadbent M, Stewart R, Corbett A, et al. Associations between risk of mortality and atypical antipsychotic use in vascular dementia: a clinical cohort study. International journal of geriatric psychiatry. 2014;29(12):1249-54.

15. Kales HC, Kim HM, Zivin K, Valenstein M, Seyfried LS, Chiang C, et al. Risk of mortality among individual antipsychotics in patients with dementia. American Journal of Psychiatry. 2012;169(1):71-9.

16. Maust DT, Kim HM, Seyfried LS, Chiang C, Kavanagh J, Schneider LS, et al. Antipsychotics, other psychotropics, and the risk of death in patients with dementia: number needed to harm. JAMA psychiatry. 2015;72(5):438-45.

17. Phiri P, Engelthaler T, Carr H, Delanerolle G, Holmes C, Rathod S. Associated mortality risk of atypical antipsychotic medication in individuals with dementia. WORLD JOURNAL OF PSYCHIATRY. 2022;12(2):298-307.

18. Langballe EM, Engdahl B, Nordeng H, Ballard C, Aarsland D, Selbæk G. Short-and long-term mortality risk associated with the use of antipsychotics among 26,940 dementia outpatients: a population-based study. The American Journal of Geriatric Psychiatry. 2014;22(4):321-31.

19. Nielsen RE, Lolk A, Valentin JB, Andersen K. Cumulative dosages of antipsychotic drugs are associated with increased mortality rate in patients with Alzheimer's dementia. Acta Psychiatrica Scandinavica. 2016;134(4):314-20.

20. Rossom RC, Rector TS, Lederle FA, Dysken MW. Are all commonly prescribed antipsychotics associated with greater mortality in elderly male veterans with dementia? Journal of the American Geriatrics Society. 2010;58(6):1027-34.

21. Nielsen, Valentin, Lolk, Andersen. Effects of Antipsychotics on Secular Mortality Trends in Patients With Alzheimer's Disease. J Clin Psychiatry. 2018;79(3).

22. Simoni‐Wastila L, Wei YJ, Lucas JA, Brandt N, Moyo P, Huang TYJ, et al. Mortality risk of antipsychotic dose and duration in nursing home residents with chronic or acute indications. Journal of the American Geriatrics Society. 2016;64(5):973-80.

23. Kheirbek RE, Fokar A, Little JT, Balish M, Shara NM, Boustani MA, et al. Association between antipsychotics and all-cause mortality among community-dwelling older adults. The Journals of Gerontology: Series A. 2019;74(12):1916-21.

24. Arai H, Nakamura Y, Taguchi M, Kobayashi H, Yamauchi K, Schneider LS, et al. Mortality risk in current and new antipsychotic Alzheimer's disease users: large scale Japanese study. Alzheimer's & Dementia. 2016;12(7):823-30.

25. Yin Y, Liu Y, Zhuang J, Pan X, Li P, Yang Y, et al. Low-dose atypical antipsychotic risperidone improves the 5-year outcome in Alzheimer's disease patients with sleep disturbances. Pharmacology. 2015;96(3-4):155-62.

26. Zakarias JK, Nørgaard A, Jensen‐Dahm C, Gasse C, Laursen TM, Palm H, et al. Risk of hospitalization and hip fracture associated with psychotropic polypharmacy in patients with dementia: a nationwide register‐based study. International Journal of Geriatric Psychiatry. 2021.

27. Koponen M, Lavikainen P, Taipale H, Tanskanen A, Tiihonen J, Hartikainen S, et al. Accumulation of hospital days among antipsychotic initiators with Alzheimer's disease. Journal of the American Medical Directors Association. 2019;20(12):1488-94. e3.

28. Lopez OL, Becker JT, Chang Y-F, Sweet RA, Aizenstein H, Snitz B, et al. The Long-Term Effects of Conventional and Atypical Antipsychotics in Patients With Probable Alzheimer’s Disease. American Journal of Psychiatry. 2013;170(9):1051-8.

29. Koponen M, Taipale H, Lavikainen P, Tanskanen A, Tiihonen J, Tolppanen A-M, et al. Antipsychotic use and the risk of hip fracture among community-dwelling persons with Alzheimer's disease. The Journal of clinical psychiatry. 2017;78(3):0-.

30. Jalbert JJ, Eaton CB, Miller SC, Lapane KL. Antipsychotic use and the risk of hip fracture among older adults afflicted with dementia. Journal of the American Medical Directors Association. 2010;11(2):120-7.

31. Mok PLH, Carr MJ, Guthrie B, Morales DR, Sheikh A, Elliott RA, et al. Multiple adverse outcomes associated with antipsychotic use in people with dementia: population based matched cohort study. BMJ. 2024;385:e076268.

32. Koponen M, Rajamaki B, Lavikainen P, Bell JS, Taipale H, Tanskanen A, et al. Antipsychotic Use and Risk of Stroke Among Community-Dwelling People With Alzheimer's Disease. J Am Med Dir Assoc. 2022;23(6):1059-65.e4.

33. Laredo L, Vargas E, Blasco AJ, Aguilar MD, Moreno A, Portolés A. Risk of cerebrovascular accident associated with use of antipsychotics: Population‐based case–control study. Journal of the American Geriatrics Society. 2011;59(7):1182-7.

34. Chan Mc, Chong CSy, Wu AYk, Wong Kc, Dunn ELw, Tang OWn, et al. Antipsychotics and risk of cerebrovascular events in treatment of behavioural and psychological symptoms of dementia in Hong Kong: a hospital‐based, retrospective, cohort study. International Journal of Geriatric Psychiatry: A journal of the psychiatry of late life and allied sciences. 2010;25(4):362-70.

35. DeMercy HM, Brenner CA. The Relationship Between Antipsychotics, Cognitive Enhancers, and Major Adverse Cardiovascular/Cerebrovascular Events (MACCE) in Older Adults with Behavioral and Psychological Symptoms of Dementia. Drugs Aging. 2024;41(10):847-58.

36. Liu M-E, Tsai S-J, Chang W-C, Hsu C-H, Lu T, Hung K-S, et al. Population-Based 5-Year Follow-Up Study in Taiwan of Dementia and Risk of Stroke. PLOS ONE. 2013;8(4):e61771.

37. Dyer AH, Murphy C, Dolphin H, Morrison L, Briggs R, Lawlor B, et al. Long-term antipsychotic use, orthostatic hypotension and falls in older adults with Alzheimer's disease. Eur Geriatr Med. 2024;15(2):527-37.

38. Tapiainen V, Lavikainen P, Koponen M, Taipale H, Tanskanen A, Tiihonen J, et al. The risk of head injuries associated with antipsychotic use among persons with Alzheimer's disease. Journal of the American Geriatrics Society. 2020;68(3):595-602.

39. Tolppanen A-M, Koponen M, Tanskanen A, Lavikainen P, Sund R, Tiihonen J, et al. Antipsychotic use and risk of hospitalization or death due to pneumonia in persons with and those without Alzheimer disease. Chest. 2016;150(6):1233-41.

40. Vigen CL, Mack WJ, Keefe RS, Sano M, Sultzer DL, Stroup TS, et al. Cognitive effects of atypical antipsychotic medications in patients with Alzheimer's disease: outcomes from CATIE-AD. American Journal of Psychiatry. 2011;168(8):831-9.

41. van de Ven‐Vakhteeva J, Bor H, Wetzels RB, Koopmans RT, Zuidema SU. The impact of antipsychotics and neuropsychiatric symptoms on the quality of life of people with dementia living in nursing homes. International journal of geriatric psychiatry. 2013;28(5):530-8.

42. Ito E, Berge LI, Husebo BS, Nouchi R, Sandvik RK. The negative impact of psychotropic drug use on quality of life in nursing home patients at different stages of dementia: Cross-sectional analyses from the COSMOS Trial. Journal of the American Medical Directors Association. 2020;21(11):1623-8.

43. Bangash A, Stubbs R, Khan F, Samnani S, Aziz H, Mitra M. Association between antipsychotics and adverse outcomes in dementia. Progress in Neurology and Psychiatry. 2017;21(4):20-6.

44. Sepassi A, Watanabe JH. Emergency department visits for psychotropic-related adverse drug events in older adults with Alzheimer disease, 2013-2014. Annals of Pharmacotherapy. 2019;53(12):1173-83.

45. Beeber AS, Zimmerman S, Wretman CJ, Palmertree S, Patel K, Sloane PD. Potential Side Effects and Adverse Events of Antipsychotic Use for Residents With Dementia in Assisted Living: Implications for Prescribers, Staff, and Families. J Appl Gerontol. 2022;41(3):798-805.

46. Nielsen RE, Valentin JB, Lolk A, Andersen K. Effects of Antipsychotics on Secular in Patients With Alzheimer's Disease. Journal of Clinical Psychiatry. 2018;79(3).
